# RNA profiles in extracellular vesicles from severe sepsis and meningitis patients reveal pathogen-specific immune signatures in meningococcal versus pneumococcal infections

**DOI:** 10.3389/fcimb.2026.1730302

**Published:** 2026-04-24

**Authors:** Berit Sletbakk Brusletto, Kari Bente Foss Haug, Iselin Sandnes Olsen, Mari Kaarbø, Ole Kristoffer Olstad, Trude Aspelin, Erik Koldberg Amundsen, Petter Brandtzaeg, Reidun Øvstebø

**Affiliations:** 1 Department of Medical Biochemistry, Oslo University Hospital, Oslo, Norway; 2 Department of Life Sciences and Health, Oslo Metropolitan University, Oslo, Norway; 3 Department of Microbiology, Oslo University Hospital, Oslo, Norway; 4 Department of Pediatrics, Oslo University Hospital, Oslo, Norway; 5 Institute of Clinical Medicine, Faculty of Medicine, University of Oslo, Oslo, Norway

**Keywords:** calprotectin (S100A8/S100A9), extracellular vesicles (EVs) in plasma, host-pathogen interactions, *Neisseria meningitidis* (meningococcus), post-mortem tissue samples, RNA profiles, septic shock (MeSH), *Streptococcus pneumoniae* (pneumococcus)

## Abstract

**Background:**

This study investigates host–pathogen interactions by comparing RNA profiles in plasma extracellular vesicles (EVs) from patients with severe sepsis or meningitis caused by *Neisseria meningitidis* with profiles from patients with systemic *Streptococcus pneumoniae* infections. At hospital admission both bacteria may present with similar symptoms, making early differentiation difficult. We have focused on EVs, as they function as active mediators of intercellular communication in the circulation.

**Methods:**

Plasma samples from patients with meningococcal septic shock, meningococcal meningitis, pneumococcal infection, and healthy controls were analyzed. EVs were isolated by size exclusion chromatography, and EV derived RNA isolated by ExoRNeasy for examination by microarray. Ingenuity Pathway Analysis searched for pathogen specific EV-RNA signatures and predicted effects on biofunctions and canonical pathways. Additionally, the plasma EV-RNA profiles from the meningococcal sepsis patients were compared with previously published transcriptomic data from post-mortem organ tissue from patients who died from *N. meningitidis* sepsis.

**Results:**

Transcriptomic profiling detected 14,909 EV-RNAs, enriched for small RNAs and canonical markers, and distinct molecular signatures across the disease groups. Patients with meningococcal septic shock displayed the most pronounced transcriptomic dysregulation, followed by milder changes in meningococcal meningitis, whereas pneumococcal disease exhibited broad pathway inhibition. S100A12, S100A8/A9 and AQP9 and plasma calprotectin were consistently elevated in all patient groups. Pathway analysis demonstrated strong activation of inflammatory and immune signaling in meningococcal septic shock, weaker activation in meningitis, and inhibition of multiple pathways in pneumococcal infection. Importantly, EV-RNA plasma profiles from meningococcal septic shock patients closely mirrored transcriptomic patterns in postmortem organ tissues detected in a previous study of lethal meningococcal shock patients.

**Conclusions:**

This study is the first to identify specific EV-RNA profiles in plasma from patients infected with *N. meningitidis* or *S. pneumoniae*. The profiles showed marked differences between the two species. In meningococcal sepsis, EV-RNA signatures in plasma were aligned with transcriptomic patterns observed in major organs suggesting that circulating EV-RNA may reflect both systemic and tissue level host responses. These findings support EV profiling as a non-invasive approach for identifying pathogen specific responses, with potential to enhance early diagnostics and clinical management of sepsis.

## Introduction

Sepsis patients meeting the criteria of *life-threatening organ dysfunction due to a dysregulated host response to infection* (Seymour et al., 2016; Singer et al., 2016) represent a highly heterogeneous group. They vary greatly in terms of infection sites, pathogens involved, and the level of bacteria in the circulation (Ovstebo et al., 2004; Loonen et al., 2017). Understanding the complex pathogenesis of this heterogeneous disease is crucial for identifying clinical scores and laboratory biomarkers that facilitate early diagnosis, classification, and management of the disease.

In this study, we focused on extracellular vesicles isolated from plasma in patients infected by *Neisseria meningitidis (N. meningitidis)* or *Streptococcus pneumoniae (S. pneumoniae)*, both bacteria that colonize the mucosal surfaces. These patients may present with hemorrhagic skin lesions, which occurs commonly in meningococcal infection and more rarely in severe pneumococcal bacteremia, at hospital admission. The clinical presentations make it challenging to differentiate between the two.

*N. meningitidis* is a capsulated Gram-negative diplococcus with lipopolysaccharide (LPS) in the outer leaflet of the outer membrane (Devoe and Gilchrist, 1973; DeVoe, 1982; Brandtzaeg et al., 2001a). During infection, *N. meningitidis* may release a substantial amount of LPS into the blood stream in the form of outer membrane vesicles (Devoe and Gilchrist, 1973; Namork and Brandtzaeg, 2002). This can trigger an excessive immune response. The concentration of meningococcal LPS in plasma correlates closely with the bacterial load and is recognized as a key driver of septic shock and multiple organ failure. Moreover, LPS levels are predictive of disease severity and case fatality rates (CFR) (Brandtzaeg et al., 1989; Brandtzaeg et al., 1992; Brandtzaeg et al., 2001a; Ovstebo et al., 2004; Hellerud et al., 2010; Brandtzaeg and van Deuren, 2012; Hellerud et al., 2015; Gopinathan et al., 2017; Gopinathan et al., 2018).

*S. pneumoniae* is a Gram-positive capsulated diplococcus characterized by a thick polysaccharide capsule, including important virulence factors. Its cell wall is primarily composed of peptidoglycans and teichoic acids, both of which contribute to its structural integrity and pathogenicity (van der Poll and Opal, 2009; Weiser et al., 2018). A key virulence factor is pneumolysin (PLY), a cytoplasmic toxin capable of being released via membrane-derived vesicles. PLY can activate immune responses by regulating numerous genes involved in the recruitment of inflammatory cells. Excessive production of early proinflammatory cytokines has been linked to organ dysfunction and increased mortality (Cima Cabal et al., 2023).

Extracellular vesicles (EVs) are nanosized particles that are secreted into body fluids from tissue and circulating cells, carrying an array of biomolecules, including proteins, RNA, DNA and lipids (Schwab et al., 2015). EVs released from the cell surface through activation and/or apoptosis usually reflect the membrane composition and content from the source cell at its physiological state. Acting as shuttles, EVs stably transfer bioactive molecules between various cell types, facilitating intercellular communication either due to signal transduction across the cell membrane or by means of component exchange (Tkach and Théry, 2016). Cellular uptake of extracellular vesicles by endocytosis, including delivery of the EV cargo, may contribute to regulation of expression and altered cellular phenotype. As such, EV uptake may lead to active reprogramming and change of function in different types of recipient cells. Whereas circulating EVs represent a heterogeneous collection of vesicles originating from potentially all cells in the body, blood cells and cells residing in the vessel and surroundings are among the most common EV secreting cells in whole blood. Platelet-derived EVs are the dominant vesicles in plasma, but leucocytes (including neutrophile granulocytes, monocytes, dendritic cells, and natural killer) and endothelial cells also contribute to the vast collection of circulating EVs (Nieuwland et al., 2000; Zafrani et al., 2013). The concentration of EVs represents a dynamic balance between formation and elimination, while the net effect of the present EVs will depend on both concentration and the EV-components carried. Endothelial- and leukocyte-derived EVs play critical roles in inflammation by promoting the synthesis of various mediators in recipient cells (Wu et al., 2013; Schorey et al., 2015).

In sepsis, both immune cells and non-immune cells such as endothelial cells and platelets produce EVs with changes in their cargo that regulate inflammatory responses, vascular endothelial function, and antigen presentation, contributing to multiple organ failure (Yáñez-Mó et al., 2015; Tian et al., 2022). Following infections, EVs can carry proteins and RNAs that affect gene expression in recipient cells (O'Brien et al., 2020). EVs may, for instance, exhibit procoagulant properties by containing tissue factor (TF) from activated monocytes, acting as mediators in promoting a procoagulant phenotype in meningococcal sepsis. These EVs play a role in the pathophysiology of disseminated intravascular coagulation (DIC) (Nieuwland et al., 2000; Hellum et al., 2014). Additionally, RNA transcript profiles in EVs from sepsis patients suggest that specific microRNAs are linked to disease progression (Gong et al., 2024), while Y-RNA subtypes may differentiate septic plasma from that of healthy individuals (Driedonks and Nolte-'t Hoen, 2018). Long non-coding RNAs (lncRNAs) may also have regulatory roles in the pathogenesis of sepsis (Lu et al., 2021).

Additionally, bacteria responsible for septic infections can produce and secrete various types of vesicles (Devoe and Gilchrist, 1973; Jan, 2017; Briaud and Carroll, 2020). Outer membrane vesicles (OMV) from Gram-negative bacteria have been shown to contain lipids including lipopolysaccharides (LPS), proteins, nucleic acids, and various virulence factors (Devoe and Gilchrist, 1973; Jan, 2017). Studies have demonstrated that OMVs released by *N. meningitidis* can induce procoagulant, profibrinolytic and antifibrinolytic factors in purified human monocytes. Such an imbalance favors fibrin deposition in the monocyte microenvironment, likely playing a crucial role in the development of DIC, micro- and macro thrombosis, hemorrhagic skin lesions and organ dysfunction in patients with *N. meningitidis* sepsis (Mirlashari et al., 2001).

Meanwhile, vesicles released from Gram-positive bacteria, such as streptococci, may carry factors related to pathogenicity and antibiotic resistance (Codemo et al., 2018; Briaud and Carroll, 2020). The small size of bacterial EVs enables them to traverse the blood-brain barrier, which is typically challenging for pathogens to penetrate (Qin et al., 2022). This ability allows bacterial EVs to deliver pathogenic molecules to host cells, where they can be internalized by recipient cells, thereby modifying their inflammatory profiles and contributing to systemic inflammation and organ dysfunction (Effah et al., 2024; Gong et al., 2024).

Research on plasma EVs in blood from sepsis patients infected with specific bacteria is, to our knowledge, limited. In the present study, patients who were critically ill and suspected of having an infectious disease were investigated for pathogens using blood culture on hospital admission. EVs were isolated from EDTA plasma and further the EV-RNA profiles were compared between patients with *N. meningitidis* or *S. pneumoniae*. The RNA profiles from the *N. meningitidis* EVs were also compared with cellular RNA profiles in tissues from large organs published previously (Brusletto et al., 2020). In this study we analyzed the RNA content in post-mortem formalin/fixed, paraffin/embedded in tissue samples from heart, lungs, kidneys, liver, and spleen from the same group of patients with meningococcal septic shock.

Analyzing RNA content in tissue samples as well as in plasma-EVs from septic shock patients may provide a new perspective for understanding the molecular mechanisms of this serious syndrome (Brusletto et al., 2020). The results could potentially lead to identification of new biomarkers released from organs into the circulation, reflecting the dysfunction of key large organs (Brusletto et al., 2020; Lu et al., 2021; Weber et al., 2023).

This study aimed to address several key questions regarding the RNA content in EVs from patients with severe infections caused by *N. meningitidis* (meningococcal infection) and *S. pneumoniae* (pneumococcal infection), both of which can lead to acute, life-threatening sepsis or meningitis. First, we investigated the differences and similarities in the RNA content of plasma EVs from patients infected with one of the two bacteria. Next, we examined whether the RNA content in circulating EVs from patients with meningococcal meningitis (characterized by low levels of bacteria and LPS in plasma) reflects the EV-RNA content in patients with meningococcal septic shock (with high bacterial and LPS levels in plasma). Furthermore, we compared the RNA content in plasma EVs from meningococcal septic shock patients with previously studied RNA content in tissues (lungs, heart, kidneys, liver, and spleen) of the same patient group (Brusletto et al., 2020). Finally, we investigated whether the RNA content in EVs reflects the production of key proteins involved in the body’s antibacterial immune response.

Our study is the first to identify pathogen-specific EV-RNA profiles in plasma collected at hospital admission from patients infected with *Neisseria meningitidis* or *Streptococcus pneumoniae*. In addition, in patients infected with *N. meningitidis*, we were able to compare pathogen-specific EV-RNA profiles in plasma to autopsy-derived transcriptomic data from major organs.

## Materials and methods

### Clinical definitions of septic shock and meningitis

Septic shock was present in a patient with hypoperfusion and an initial systolic blood pressure of < 85 mmHg requiring fluid and vasoactive drug treatment (dopamine or dopamine and epinephrine) for at least 24 h or until death.

Meningitis was present in a patient with clinical signs of neck or back rigidity, positive signs of Kernig and/or Brudzinski and pleocytosis (leukocytes >10^8^/L) of the cerebrospinal fluid (CSF).

Systemic pneumococcal disease was diagnosed if either septic shock, meningitis or both were present on hospital admission.

The bacteria were diagnosed by positive culture of *N. meningitidi*s or *S. pneumoniae* in the blood and/or CSF.

### Study participants

**Figure 1:**
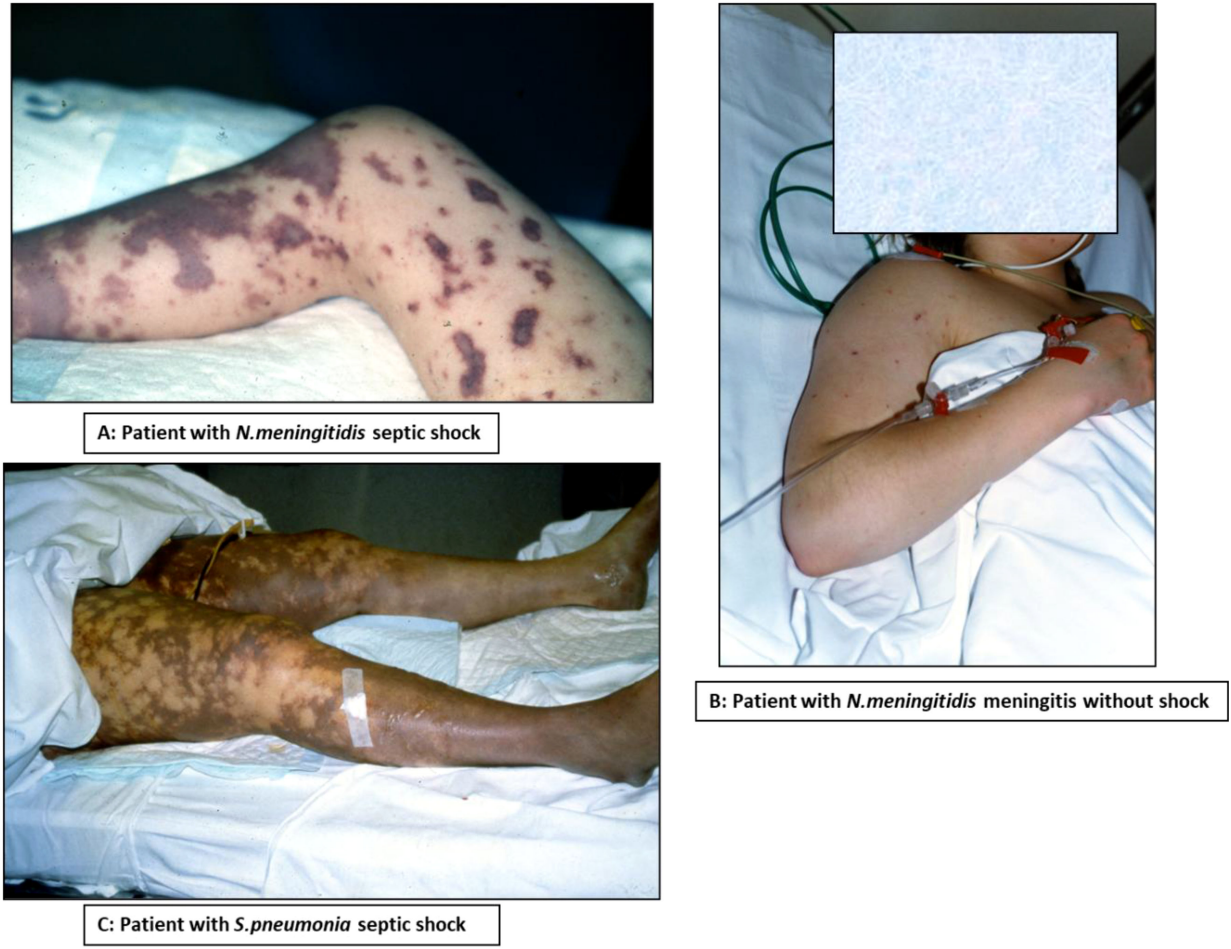
Clinical symptoms in patients with: **(A)***N.meningitidis* septic shock, **(B)***N.meningitidis* meningitis, **(C)***S. pneumoniae* septic shock. The patient or parents/relatives to the child/patient consented to be photographed. Reproduced with permission from Petter Brandtzag; MD; PhD.

The Blood Cell Research group at Oslo University Hospital has been conducting studies on meningococcal disease for decades. In these studies, the majority of the patients presented with a hemorrhagic skin rash or ecchymosis and clinical and laboratory signs of a life-threatening infection at the Department of Infectious Diseases or Department of Pediatrics at Ullevål, Oslo University Hospital between 1985 and 2001, during the meningococcal epidemic in Norway. Occasionally pneumococcal infections may mimic meningococcal infections (**Figure 1**). Blood cultures and EDTA blood samples were collected at hospital admission. EDTA plasma was used for analysis of RNA, quantification of *S. pneumoniae*, and calprotectin. Samples from patient Nr. 5 and Nr. 6 were collected 20 and 8 hours, respectively, after admission to hospital (**Table 1**). Patients with confirmed *N. meningitidis* were divided into meningococcal septic shock or meningitis groups based on their clinical definitions (Brandtzaeg et al., 1989; Brandtzaeg and van Deuren, 2012). The FFPE tissues (n=6) from lungs, heart, kidneys, liver, and spleen from a previously autopsy study, used for comparing RNA profiles in tissues and in EV-plasma, were from the meningococcal septic group, though they came from different patients (Brusletto et al., 2020). Four of the patients died within 12 h of hospital admission. The samples were collected during the routine post mortem examination within 24 h after the patient died. Tissue samples from different organs were fixed in 4% buffered–neutral formalin at room temperature for 6–48 h, dehydrated, cleared, embedded in paraffin and cut in 4µm thick sections.

**Table 1:** Clinical characteristics of patients at admission to hospital with meningococcal septic shock, meningococcal meningitis, systemic pneumococcal disease and healthy controls.

Sample No	Sample group	Age at admission to hospital (years)	Mortality	Sero-group	Blood/CSF/Throatculture	LPS EU/mL	Neisserial DNA: copy number of *N.meningitidis*/mL plasma	Pneumococcus DNA: copy number of *S.pneumoniae/mL plasma*
13	Meningococcal septic shock	< 1	Yes	C	Blood Positiv	125	4.1x10^7^	Not performed
14	Meningococcal septic shock	14	No	B	Blood Positiv	2.1*	2.9x10^6^	Not performed
15	Meningococcal septic shock	12	Yes	B	Blood Positiv	105	2.0x10^7^	Not performed
16	Meningococcal septic shock	14	No	C	Blood Positiv	38	1.3x10^7^	Not performed
17	Meningococcal septic shock	20	Yes	B	Blood Positiv	1,700	Not performed	0
18	Meningococcal septic shock	< 1	Yes	B	Blood Positiv	1,505	5.4x10^8^	Not performed
7	Meningococcal meningitis	49	No	A	CSF Positiv	<0.2	1.1x10^4^	Not performed
8	Meningococcal meningitis	25	No	B	Blood Positiv	<0.003	Not performed	Not performed
9	Meningococcal meningitis	22	No	B	Throat Positiv	<0.2	<10^3^	Not performed
10	Meningococcal meningitis	17	No	C	CSF Positiv	<0.003	<10^3^	Not performed
11	Meningococcal meningitis	20	No	B	Blood Positiv	<0.003	Not performed	0
12	Meningococcal meningitis	16	No	B	Blood Positiv	<0.2	1.1x10^3^	Not performed
1	Systemic pneumococcal disease	57	Yes	–	Blood Positiv	–	–	49,689
2	Systemic pneumococcal disease	15	Not available	–	Blood Positiv	–	–	0
3	Systemic pneumococcal disease	26	Not available	–	Blood Positiv	–	–	179
4	Systemic pneumococcal disease	55	Not available	–	Blood Positiv	–	–	13,787
5	Systemic pneumococcal disease	6	Not available	–	Blood Positiv	–	–	27
6	Systemic pneumococcal disease	34	Not available	–	Blood Positiv	–	–	2, 900
19	Healthy control	Not available	–	–	Not applicable	–	–	0
20	Healthy control	Not available	–	–	Not applicable	–	–	0
21	Healthy control	Not available	–	–	Not applicable	–	–	0

*First plasma sample collected 18 hours after antibiotic treatment was started. The median half-life of circulating *N. meningitidis* LPS in shock patients is 2 hours (range 1–3 hours) Brandtzaeg 1989 doi. doi.org/10.1093/infdis/159.2.195. Red shade represents Meningococcal septic shock, blue shade represents Meningococcal meningitis, green shade represents Systemic pneumococcal disease, yellow shade represents Healthy control.

Patients with confirmed *S. pneumoniae* infections included three patients with primarily sepsis symptoms (P1, P3, P6, **Table 1**) and three patients (P2, P4, P5, **Table 1**) with primarily meningitis signs and symptoms. Patient 3 was splenectomized and unvaccinated. After the initial examination and treatment and the confirmed bacterial cause was established as *S. pneumoniae*, they were not included in further studies of meningococcal pathophysiology, and the outcome is presently not known except for P1 who died few hours after admission to the Intensive Care Department. Since several of the patients had mixed symptoms of sepsis and meningitis, the six patients have been analyzed as one group. The plasma samples were stored in our biobank at -80 °C and analyzed with permission from the Regional Committee for Medical and Health Research Ethics (REC). Blood samples were centrifuged within 2 hours at 1,400 x g for 10 minutes before plasma aliquoting and storage at -80 °C. Samples were collected from patients diagnosed with meningococcal septic shock (n=6), meningococcal meningitis (n=6), and systemic pneumococcal disease (n=6) for inclusion in this project.

In addition, healthy controls from blood donors (n=3), collected in 2013, were included in this project (**Table 1**). Blood samples were centrifuged within 2 hours at 1,400 x g for 10 minutes before plasma aliquoting and storage at -80 °C. After thawing, patient samples and healthy controls tubes were centrifuged at 10,000 x g for 10 minutes before supernatants were further analyzed.

### Detection of DNA and LPS of *N. meningitidis* in plasma

Blood samples were previously measured for circulating *N. meningitidis* DNA in plasma via quantitative PCR (qPCR) (Ovstebo et al., 2004; Gopinathan et al., 2012). Measurement of lipopolysaccharide (LPS) levels in heparin plasma was initially done using an in-house Limulus Amebocyte Lysate (LAL) assay with a detection limit of 0.2 EU/mL, and later with Chromo-LAL kit (Associates of Cape Cod, USA) with a detection limit of 0.003 EU/mL (Brandtzaeg et al., 1989; Brandtzaeg et al., 2001b).

### Quantification of *Streptococcus pneumoniae* in plasma using digital droplet PCR

EDTA plasma samples were from patients with systemic pneumococcal disease (n=6, sample 1-6, **Table 1**), *N. meningitidis* (Nm) meningitis (n=1, sample 11, **Table 1**), Nm septic shock (n=1, sample 17, **Table 1**), and healthy controls (n=3, sample 19-21, **Table 1**). A volume of 150 µL of plasma from each sample was extracted using a modified protocol of the NAxtra™ Total Nucleic Acid Extraction Kit (Lybe Scientific, Trondheim, Norway). The protocol included a 10-minute boiling step after mixing the plasma with the lysis buffer. The samples were then eluted in 50 µL of molecular-grade H_2_O for subsequent ddPCR analysis.

*S. pneumoniae* was quantified by ddPCR using the QX200 system (Bio-Rad, California, USA) following the manufacturer’s instructions. The reaction mixture comprised 5 µL of the extracted template, Bio-Rad’s Supermix for Probes without dUTP (# 1863024), and a final concentration of 900 nM for both forward and reverse primers and 250 nM for the FAM-labeled probe. Primer and probe sequences targeting lytA are detailed below (Sheppard et al., 2004). The annealing temperature for the reactions was 59 °C (Ziegler et al., 2019). Each sample was analyzed in at least triplicates. The number of *Streptococcus pneumoniae* was expressed as copy number/mL of heparin plasma.

Primer and Probe Sequences (Sheppard et al., 2004):

lytA Forward Primer: CAGCGGTTGAACTGATTGA,

lytA Reverse Primer: TGGTTGGTTATTCGTGCAA,

lytA Probe (FAM): AGCTGGAATTAAAACGCACGAG.

### EV isolation and EV-RNA isolation

EVs were isolated from the plasma samples using size-exclusion chromatography (SEC) with a qEV original 70 nm column and Izon Automatic Fraction Collector (AFC) (Izon Science, Oxford, UK). For each sample, 500 μL of plasma was applied to the column, and EVs were eluted using 0.1 µm filtered PBS. Fractions of 500 μL were collected, and fractions 2, 3 and 4 containing EVs (Böing et al., 2014), were merged together to one “EV joint fraction” and stored at -80 °C for further EV characterization and analysis.

The ExoRNeasy Serum/Plasma Midi Kit (Qiagen), designed for the combined isolation of vesicles and total RNA from EVs, was used to isolate total RNA from the EVs. A volume of 1,000 μL of “EV joint fractions” was added to 1,000 μL Buffer XBP, mixed, and applied to an exoRNeasy spin column according to the manufacturer’s instructions. The RNA eluate was kept on ice for further quantification and characterization using a Qubit Fluorometer and an Agilent 2100 Bioanalyzer and further stored at -80 °C until Affymetrix analysis.

### Characterization of EVs

The characterizations of EVs were performed in accordance with the Minimal Information for Studies of Extracellular Vesicles (MISEV) 2018 guidelines (Théry et al., 2018). To accommodate all required characterization methods and ensure data quality, a small volume of all samples in each group were pooled to obtain sufficient material for the selected analyzes. The plasma available from each patient was only 500 μL, which had to be used for all analyzes included in this study.

### Nanoparticle tracking analysis

Size distribution and concentration of the plasma EVs from all patients were determined in the “SEC joint EV fractions” by nanoparticle tracking analysis (NanoSight NS500, Malvern Instruments Ltd., Amesbury, UK). The samples were diluted with 0.02 μm filtered PBS (Whatman™ Anotop™ 25, GE Healthcare Life Science, Buckinghamshire, UK) to achieve the recommended vesicle concentration levels for analysis. The camera level was set to 13, the detection threshold was set to 3. Triplicate videos of 60 seconds (s) each of a continuous flow of sample were recorded. Data analysis was performed using NTA 3.1 software. The analysis demonstrated an intra-assay coefficient of variance (CV) below 7% and an inter-assay CV (day-to-day) of less than 20% (Vestad et al., 2017).

### Transmission electron microscopy

Transmission electron microscopy (TEM) of EVs was conducted on pooled SEC “EV joint fractions” from each patient group were analyzed for EV morphology using a Technai G2 Spirit transmission electron microscope (FEI, Hillsboro, OR, USA). Images were captured using a Morada digital camera and RADIUS imaging software (http://emsisasia.com/radius/) and further processed using Adobe Photoshop (Aas et al., 2023).

### Western blotting

Western blotting was used for detection of CD9, Hsc70/Hsp70, syntenin-1, and calnexin in 20 µl “EV joint fractions”, and an in-house SW480 cell lysate (lysed in RIPA buffer) (Bjornetro et al., 2021), as well as recombinant EV standard (Merck Life Science, Oslo, Norway).

### EV-RNA quantification and quality check

RNA was isolated from “EV joint fractions” by ExoRNeasy serum/plasma Midi Kit (Qiagen) and the eluate was quality checked using an Agilent 2100 Bioanalyzer and the Agilent RNA 6000 Pico Assay Kit (Agilent Technologies, Santa Clara, CA, USA) in accordance with the manufacturer’s protocol. The concentration of RNA was also checked by Qubit^®^ (Qubit™ microRNA assay kit) and a Nanodrop™ One Spectrophotometer (Thermo Fisher Scientific, Oslo, Norway). RNA from EVs were found to be fragmented and small sized (as expected).

### Extracellular vesicle RNA analysis

EV-RNA profiling was carried out using the Affymetrix GeneChip Human Transcriptome 2.0 Array (HTA 2.0) platform (Thermo Fisher Scientific, Oslo, Norway). Experience from EV-RNA isolation by combined SEC and ExoRNeasy serum/plasma Midi Kit from the same volume of plasma samples showed low and varying RNA concentrations from all patients and controls. Due to this, we decided to use the same volumes of isolated RNA into microarray analysis. The GeneChip WT Pico Reagent Kit, in accordance with the manufacturer’s instructions for whole-transcriptome analysis, was used and enabled us to analyze fragmented RNA samples in low concentrations. The input of RNA for single-stranded cDNA synthesis was then based on equal volumes (3 μL) instead of concentration.

The analysis was performed with twelve PCR cycles for pre- *in vitro* transcription (IVT) amplification for all samples, which is recommended for RNA inputs between 100 pg and 500 pg. A HeLa cell sample was used as a positive control with a total cellular RNA input of 0.5 ng and nuclease-free water served as the negative control. To ensure quality and process consistency, external controls—such as commercially poly-A RNA spikes and hybridization controls—were added to each sample. After labeling and fragmentation, the same amount of single-stranded cDNAs from all the samples were hybridized to the arrays. Washing and staining were completed using a FS-450 fluidics workstation (protocol FS450_0001, Affymetrix). Signal detection was performed with a Hewlett Packard 30007G scanner (Palo Alto, CA, USA), and raw data files were processed via Affymetrix GeneChip Command Console software. For downstream data analysis, the results were imported into Partek^®^ Genomics Suite™ (Partek Inc., St. Louis, MO, USA). Signal intensities were normalized using the Robust Multichip Analysis (RMA) algorithm. The HTA 2.0 array allowed detection of up to 44,699 protein-coding genes and 22,829 non-coding RNAs, including immature microRNAs.

### Bioinformatic processing and statistical analysis

Following quality control and pre‐processing, transcripts with maximal signal values < 6 (log2) across all arrays were removed to filter for low-abundance or undetected transcripts. A one-way ANOVA was applied to identify different transcript levels between patient and control groups, with significance thresholds set at fold change (FC) ≥ |1.5| and P < 0.05. Further exploratory data analysis included principal component analysis (PCA) to assess sample clustering, and Venn diagrams to visualize sharing and unique EV-RNA transcripts among groups.

### Functional interpretation using ingenuity pathway analysis

The annotated expression data, including gene IDs and statistical metrics, were uploaded into Ingenuity^®^ Pathway Analysis (IPA) (IPA; QIAGEN Inc. https://www.digitalinsights.qiagen.com). The tool mapped transcripts to known biological entities within the Ingenuity Knowledge Base, allowing for interpretation of gene function, upstream regulators, and pathway involvement. Fisher’s exact test was used to evaluate the likelihood that observed associations were due to random chance, with Benjamini–Hochberg correction applied to adjust for multiple comparisons. Z-scores were calculated to predict activation or inhibition of biological functions and signaling pathways, with absolute *z*‐score ≥ 2 or ≤-2 considered significant. Enrichment analysis was performed using IPA’s ≪core analysis≫ for each patient group and a ≪comparison analysis≫ between the three patient groups. These functions can identify and predict significantly activated biological functions and pathways, molecular functions, and relationships in our dataset of transcripts eligible for IPA analysis.

The Regulator Effects algorithm connects upstream regulators, dataset molecules and downstream functions or diseases affected in the dataset to generate a hypothesis that can explain how the activation or inhibition of an upstream regulator affects the downstream target molecule expression and the impact of the molecular expression on functions and diseases. Networks were only integrated when target overlap met statistical relevance (P < 0.05), and were ranked by a Consistency Score, which reflects the coherence of predicted cause–effect relationships based on known biological interactions. Higher scoring hypotheses are those with more consistent causal paths represented by a high Consistency Score (Krämer et al., 2014). A consistent relationship is one in which the direction of node activity/expression that is observed or predicted is consistent with the direction one would expect based on the findings from the Ingenuity Knowledge Base. The Consistency Score is not itself a measure of statistical significance, but a heuristic to rank a set of already statistically significant networks within the same analysis and settings (QIAGEN).

### Validation of affymetrix microarray results

To confirm the differential RNA levels observed in the microarray dataset, a subset of transcripts showing the highest fold changes between groups was selected for Reverse Transcription (RT)- quantitative PCR (qPCR) validation. TaqMan^®^ gene expression assays were used in combination with the Applied Biosystems ViiA™ 7 Real-Time PCR System to assess transcript levels across all sample groups. For validation of mRNA transcript expression, total EV-RNA (0.2ng) was reverse transcribed using SuperScript IV VILO cDNA Syntheisis Kit (ThermoFisher Scientific). The resulting cDNA was subjected to qPCR in 20 µL reactions performed in duplicate, using TaqMan^®^ Fast Advanced Master Mix (Applied Biosystems, Foster City, CA, USA). The specific primer-probe sets used were: S100A12 (Hs00942835_g1), S100A9 (Hs00610058_m1), S100A8 (Hs00374264_g1) and AQP9 (Hs01033361_m1). Relative expression levels were calculated using the ΔΔCt method (Livak and Schmittgen, 2001), with MXRA7 (Hs00895155_m1) and LMNA (Hs00153462_m1) serving as internal reference genes. Data analysis was conducted using ViiA™ 7 Software version 1.2.

### Measurement of calprotectin (S100A8/A9)

Due to highly expressed S100A8/A9 transcripts in all three patient groups in our study, a validation check for expression of the protein calprotectin in plasmas were performed. EDTA plasma samples were thawed and measured in parallel for p-calprotectin with a Roche Cobas c501 ^®^ (Roche, Basel, Switzerland) analyzer, using the particle enhanced turbidimetric assay (CGAL) (Gentian Diagnostics AS; Moss, Norway). Three extra samples were included in the healthy control group.

### Comparison of transcript profiles of plasma EVs in meningococcal septic shock patients and in tissue samples from meningococcal septic shock patients

RNA profiles in plasma EVs from meningococcal septic shock patients in the present study and RNA profiles in tissue cells from a previously published human meningococcal septic shock autopsy study where FFPE (n = 5) tissues from lungs, heart, kidneys, liver, and spleen were compared (Brusletto et al., 2020). The meningococcal septic shock patients were included with the same clinical definitions and criteria (Brandtzaeg et al., 1989; Brandtzaeg and van Deuren, 2012). Both studies are compliant with the minimum information about microarray experiment (MIAME) guidelines (Brazma et al., 2001) regarding GeneChip Human Transcriptome Array (HTA) 2.0 microarrays and protocols used. The IPA comparison of the RNA profiles of plasma EVs and of tissue cells in the study were based on FC ≥| ± 1.5| and p-values < 0.05.

### Statistical analysis

GraphPad Prism, version 10 was used for the box and whiskers graphs with median values (GraphPad Software lnc),.

## Results

### Quantification of *Neisseria meningitidis*, LPS and *Streptococcus pneumonia* in plasma or CSF samples

Fifteen out of the 18 patients had positive blood cultures. Three patients with meningococcal meningitis had negative blood cultures, but two had a positive cerebrospinal fluid (CSF) culture and one was throat-swab positive (**Table 1**).

In our previous publication (Ovstebo et al., 2004) *N. meningitidis* DNA was detected in five septic shock patients, while the assay was not performed for one patient. DNA copy numbers varied between 2.9 x 10^6^ and 5.4 x 10^8^ copies per milliliter of plasma. Among the meningitis patients, one individual exhibited 1.1 x 10^4^ copies/mL in plasma, despite having a negative blood culture and a positive cerebrospinal fluid (CSF) culture. In five other plasma samples, DNA levels fell below the detection limit (<10^3^/mL plasma) (**Table 1**).

LPS concentrations in plasma from patients with meningococcal sepsis ranged from 38 to 1,700 EU/mL, whereas all meningitis patients had LPS levels below the detection threshold of 0.2 EU/mL (**Table 1**).

*Streptococcus pneumoniae (S. pneumoniae)* was quantified using ddPCR across different patient groups and healthy controls (**Table 1**). In the severe systemic pneumococcal disease group, *S. pneumonia* was detected in five out of six patient samples, with copy numbers ranging from 27 to 49,689 copies of *S. pneumoniae*/mL plasma. *S. pneumoniae was* not detected in the *N. meningitidis* sepsis patients, the *N. meningitidis* meningitis patients, nor in healthy controls (**Table 1**).

### Characterization of EVs isolated from EDTA plasma by size exclusion chromatography

**Figure 2:**
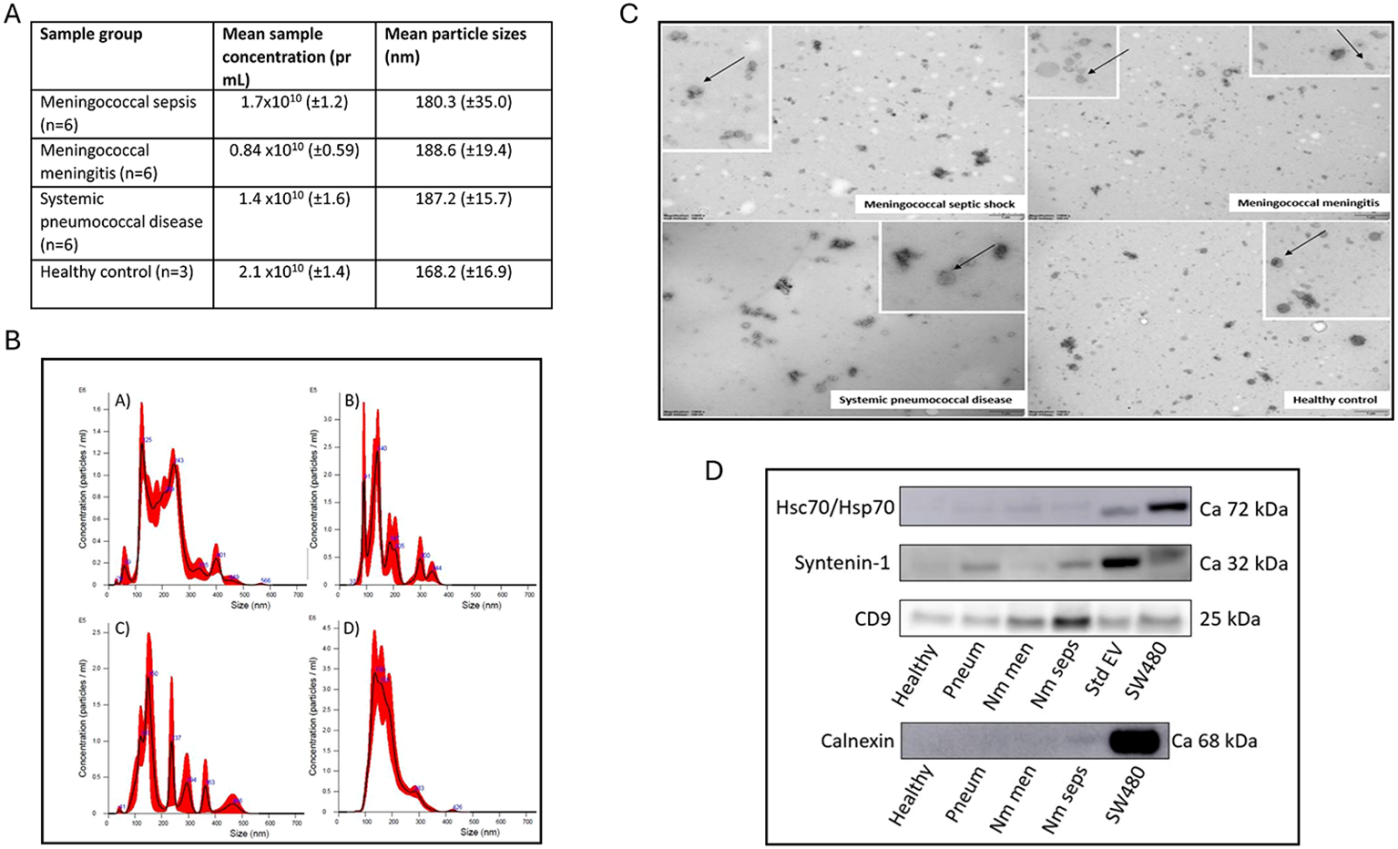
Characterizations of extracellular vesicles (EVs) **(A)** Mean size and particle concentration of plasma EVs isolated by SEC joint fractions for patient group and healthy controls measured by NTA. **(B)** Graphs of particle concentration (particles pr mL) plotted against particle size (nm) measured with NTA in SEC joint fractions from representative samples from each group: **(A)** Meningococcal septic shock, **(B)** Meningococcal meningitis, **(C)** Systemic pneumococcal disease, **(D)** Healthy control. All samples were diluted 1/100 in diluent (filtered PBS). **(C)** Transmission Electron Microscopy (TEM) images of uranyl acetate stained EVs from pooled SEC joint fractions from each patient group and healthy controls. Images are magnified 13,000 x, and all scale bars in the figure represent 1 μm. The arrows indicate representative free vesicles. **(D)** Western Blotting analysis of EV proteins from SEC joint fractions isolated from patient group, healthy controls, a recombinant standard EV solution and a SW480 cell lysate, with generic markers Hsc70/Hsp70, Syntenin-1, CD9 and the non-EV marker calnexin. Nm seps = *Neisseria meningitidis (N. meningitidis)* septic shock patients. Nm men = *Neisseria meningitidis (N. meningitidis)* meningitis patients. Pneum = *Streptococcus pneumoniae (S. pneumoniae)* Systemic pneumococcal disease patients. adapted and modified with permission from the Master thesis:” RNA profiles of circulating extracellular vesicles in patients with meningococcal sepsis” written by Iselin Sandnes Olsen, May 2023, Faculty of Health Sciences, Oslo Metropolitan University (Olsen, 2023).

All EV samples, distributed in the meningococcal septic shock (n=6), meningococcal meningitis (n=6), systemic pneumococcal disease (n=6) and the healthy control (n=3) groups were analyzed using Nanoparticle Tracking Analysis (NTA). The mean particle size and concentrations are presented in **Figures 2A, B**.

Transmission Electron Microscopy (TEM) images of isolated EVs were obtained from pooled SEC joint fraction samples from each patient group and the healthy control. The images showed that EV material was abundant in all patient groups; double membrane vesicles with a cup-shaped morphology (**Figure 2C**). Free vesicles and some aggregates were observed in all patient groups and controls. Variable presence of lipoprotein particles and proteins aggregates co-eluted with the EVs were identified by non-collapsed very round shapes (Vestad et al., 2021). In our study, EV-RNA was isolated from SEC joint fractions using the ExoRNeasy serum/plasma Midi kit, which may have contributed to further reduced the presence of lipoprotein contamination.

Western blotting analysis demonstrated heat-shock protein Hsc70/Hsp70 in all patient samples, but not in the healthy control (**Figure 2D**). Syntenin-1 and CD9 were detected in all patient samples, in the healthy control, recombinant EV solution, and in the SW 480 lysate control, as expected. Calnexin was detected in the SW480 lysate but showed no or very weak bands for each of the pooled patient samples as expected (**Figure 2D**). Characterization of SEC isolated EVs from plasma fulfilled the MISEV requirements.

### Characterization of RNA from EVs

RNA was successfully isolated from all EV samples and showed substantially small sized fragments in the electropherograms of the Bioanalyzer using the Agilent RNA 6000 Pico Assay Kit (data not shown).

### Microarray analysis of plasma EV-RNA

#### Descriptive analysis

**Figure 3:**
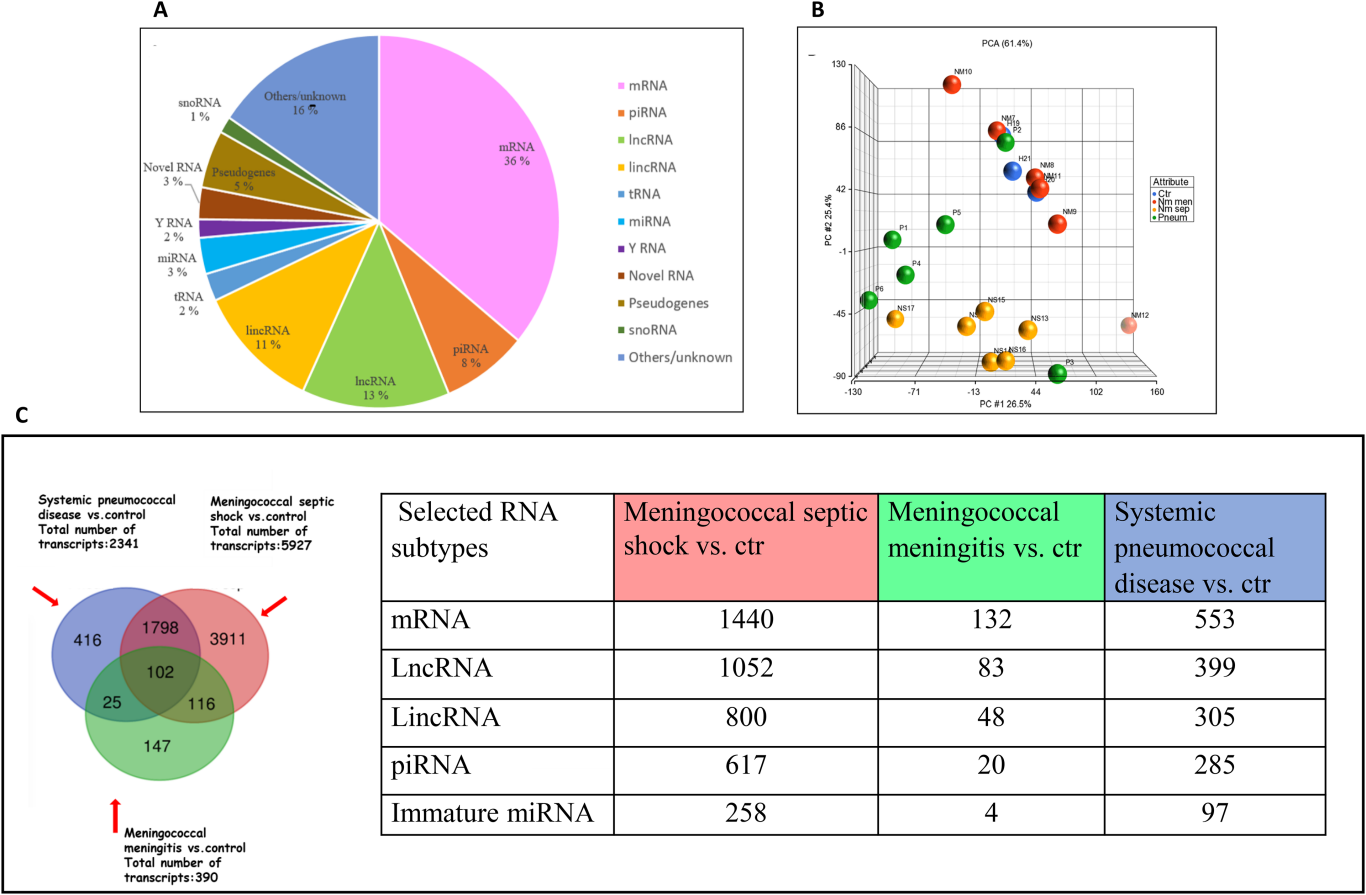
Distribution of RNA subtypes in patients and healthy controls. **(A)** Distribution of RNA subtypes detected in isolated EV-RNA of all sample groups with Affymetrix log2 signal values ≥ 6. **(B)** Principal Component Analysis (PCA) plot generated from detected signal values of EV-transcripts from healthy control group (n=3) in blue, meningococcal septic shock patients (n=6) in yellow, meningococcal meningitis patients (n=6) in red and systemic pneumococcal disease patients (n=6) in green. Filtering criteria: signal values < 6 (log2). Each circle represents individual samples, and the distance between them represent differences in the transcript expression patterns. The percentage value describes the proportion of the total variance described by each principal component (PC) axis (PC1, PC2 and PC3). The three PCs explain 61.4% of the variance in the data. **(C)** Venn diagram of EV-transcripts presents in plasma from the meningococcal sepsis, meningococcal meningitis and systemic pneumococcal disease patient groups compared to the healthy control group. Filtering criteria: signal values of <6 log2. For expression comparisons of different groups: one-way ANOVA model, P-value <0.05, FDR 5%. Created in http://bioinformatics.psb.ugent.be/webtools/Venn/. The table display number of selected subtypes of RNA between patient group and healthy control. Filtering criteria: signal values < 6 (log2), p-value 0.05, FDR of 5% adapted and modified with permission from the Master thesis:” RNA profiles of circulating extracellular vesicles in patients with meningococcal sepsis” written by Iselin Sandnes Olsen, May 2023, Faculty of Health Sciences, Oslo Metropolitan University (Olsen, 2023).

The GeneChip Human Transcriptome Array 2.0 was used for transcriptomic analysis of EV-RNAs. After filtering for signal values ≥ 6, 14,909 transcripts were detected. The RNA transcripts were categorized according to their RNA subtypes, based on the transcript information in the CEL-files and are presented in a sector diagram (**Figure 3A**). The most abundant RNA types (**Figure 3A**) were mRNA (5,366 transcripts), lncRNA (1,847 transcripts), long intergenic non-coding RNA (lincRNA) 1,661 transcripts, Havana chromosome (1,167 transcripts) and piRNA (1,147 transcripts).

Based on the results from the microarray data, a principal component analysis (PCA) was used to visualize variations within and differences between the EV-RNA profiles in the different groups of patients and controls (**Figure 3B**). The PCA analysis showed that the overall amount of RNA profiles was different across three of the groups, where the systemic pneumococcal disease group was more heterogeneous than the meningococcal septic shock group. The meningococcal meningitis patients clustered almost similar with the control group.

Based on different signal value levels between the transcripts detected in the patient groups and the healthy control group, the Venn diagram in **Figure 3C** illustrates that 5,927 EV-RNAs had significantly different signal levels in the meningococcal septic shock group, 390 in the meningitis group and 2,341 in the systemic pneumococcal disease group. Both mRNAs and a range of different ncRNAs were represented, including lncRNAs, lincRNAs, piRNAs, and immature miRNAs as some of the most abundant RNA subtypes detected. The meningococcal septic shock group demonstrated the highest number of RNAs with significantly different levels compared to the control group, followed by the systemic pneumococcal disease group, where the meningococcal meningitis group had the least number of significant transcripts (**Figure 3C**).

The top 50 EV-RNAs with significantly different signal levels (up-and down), based on fold change of each patient group as compared to the healthy control group, showed that piRNA transcripts (several similar) were on top in both the meningococcal septic shock group and the systemic pneumococcal disease group (**Supplementary File 1**). lncRNA, snoRNA, MIR4461 were most pronounced in the meningococcal septic shock group, second highest in the systemic pneumococcal disease group, while a snoRNA transcript and a lncRNA transcript were on top in the meningitis and systemic pneumococcal disease group, respectively. Of the top 50 transcripts, 14 transcripts were similar in the meningococcal septic shock group, and 41 transcripts were similar in the systemic pneumococcal disease group, respectively; however, they were present with different levels. In both groups, mRNAs for PPBP, PF4, MTRNR2L8, MTRNR2L2, and TUBB1 were detected at lower levels compared to the healthy controls. An interesting result is the highly abundant transcripts S100A12, S100A9/A8, and AQP9, which were consistently featured among the top listed in all the patient groups (**Supplementary File 1**, **Table 2**).

**Table 2:** List of the top 10 transcripts with fold change (FC) values in plasma EVs.

Meningococcal septic shock patients vs. healthy control group			
Transcripts	Fold change (FC) increased values	Transcripts	Fold change (FC) reduced values
SNORD93	13.9	MT-CYB	-25.3
MCEMP1	12.2	PPBP	-10.6
OR2J2*	12.1	PF4	-8.7
ZZEF1	11.2	MTRNR2L8	-8.7
DUX4*	10.7	TUBB1	-8.0
OR9A1P	9.8	MTRNR2L2	-7.6
S100A12	9.7	RGS18	-7.6
MMP8	9.7	SH3BRL2	-6.1
ERVK-21	8.6	NRGN	-5.9
AQP9	8.5	B2M*	-5.8
Meningococcal meningitis patients vs. healthy control group			
Transcripts	Fold change (FC) increased values	Transcripts	Fold change (FC) reduced values
CXCR2	7.0	MBNL3*	-2.2
S100A12	5.4	BLZF1	-2.2
S100A9	4.2	GAB3	-2.1
IFITM2*	4.0	TLE5*	-2.0
AQP9	3.9	GSPT1*	-1.9
CREB5	3.8	MPDU1*	-1.8
MNDA	3.6	LINC01063	-1.7
SAMSN1	3.5	PRKCSH*	-1.7
RGS2	3.0	TCF3*	-1.7
RNASE2CP*	2.8	CCNT2-AS1	-1.6
Systemic pneumococcal disease patients vs. healthy control group			
Transcripts	Fold change (FC) increased values	Transcripts	Fold change (FC) reduced values
OR9A1P	6.9	MT-CYB	-35.9
DUX4*	6.8	MTRNR2L8	-12.9
S100A12	5.4	MTRNR2L2	-10.2
CLEC4E	5.3	PPBP	-7.1
ZZEF1	5.3	TUBB1	-7.0
USP17L23	5.2	PF4	-6.9
AQP9	4.7	RGS18	-6.7
MCEMP1	4.5	B2M*	-5.7
PAAF1	4.0	MTRNR2L1*	-5.3
S100A9	4.0	SH3BGRL2	-5.1

List of the top 10 transcripts with fold change (FC) values in plasma EVs from meningococcal septic shock-, meningococcal meningitis-, and systemic pneumococcal disease patients compared to the healthy control group. Analysis performed by IPA. P-value of overlap comparing fold change values from meningococcal septic shock (n=6) -, meningococcal meningitis (n=6)-, and systemic pneumococcal disease (n=6) patients with healthy control group (n=3). (Filtering criteria: FC ≥| ± 1.5|, p< 0.05). Transcripts may refer to any gene or RNA *: Duplicates -Gene/Protein/Chemical identifiers marked with an asterisk indicate that multiple identifiers in the dataset file map to a single gene/chemical in the Global Molecular Network.

The top ten mRNAs with significantly changed levels in patient groups as compared to the control group (**Table 2**) were found in the meningococcal septic shock patients, followed by the systemic pneumococcal disease group and the meningitis patient group. Notably, again in all three patient groups, S100A12 and S100A9 were identified. Also, the AQP9 mRNA levels were affected in all patient groups. Among the top transcripts with lower levels in the meningococcal and systemic pneumococcal disease patients was MT-CYB (Mitochondrially Encoded Cytochrome B), a protein that is located in mitochondria and involved in electron transport processes and possibly involved in cardiomyopathy (Wikipedia).

### “Core analysis” - prediction of biofunctions and canonical pathways

The “core analysis” (**Supplementary Files 2A–C**) for the biofunctions in patients with meningococcal septic shock revealed broad activation across cellular pathways with the most significant Z-scores observed in cellular movement, immune cell trafficking, hematological system development, inflammatory responses, and cell-to-cell signaling and interactions. In contrast, the meningitis group showed a much weaker response in these same biofunctions. In the systemic pneumococcal disease group, the same biofunctions were substantially decreased (inhibited).

**Figure 4:**
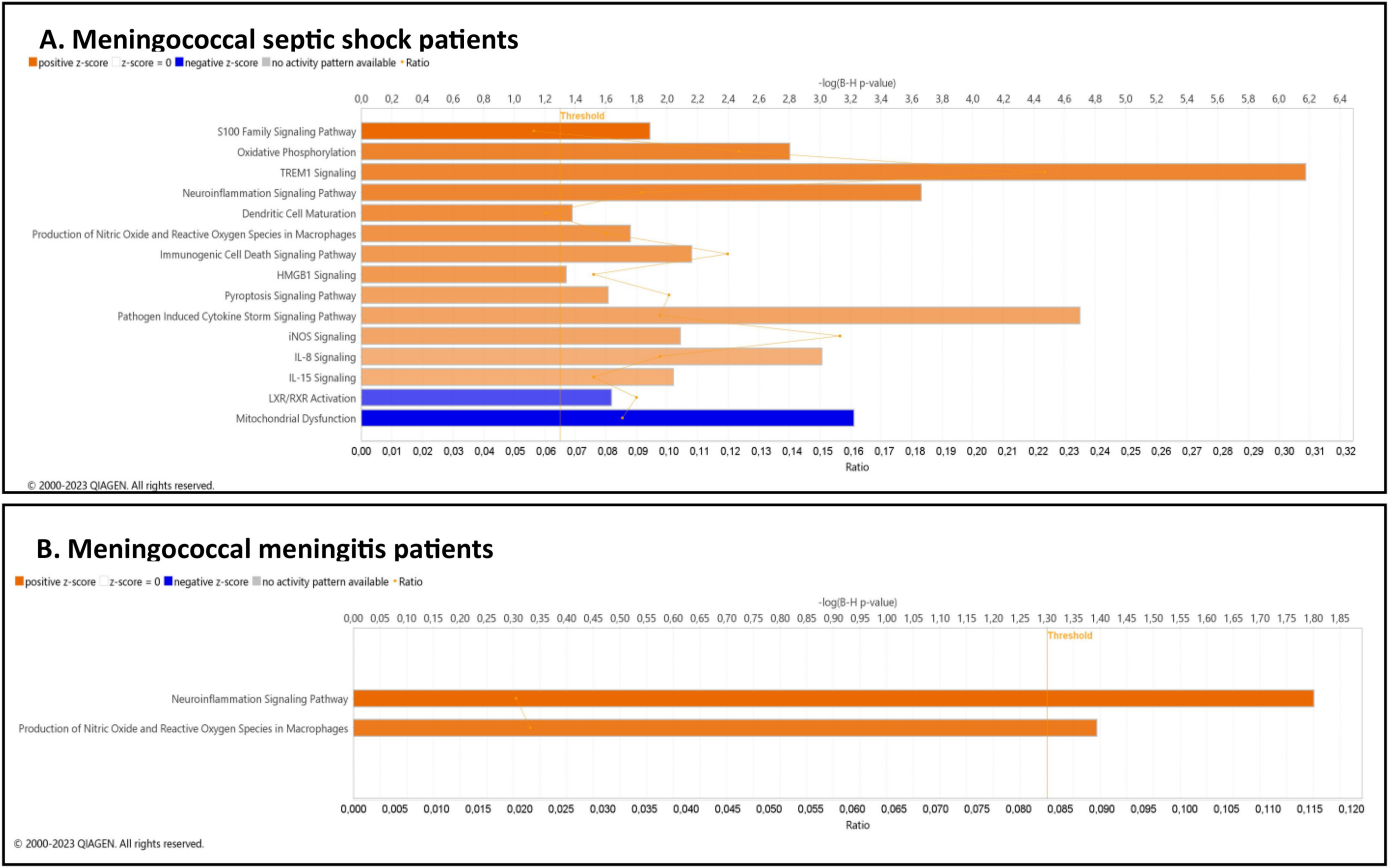
**(A, B)** Predicted effects on canonical pathways based ≪core≫ analysis of the significant difference in EV-transcript patterns in the **(A)** meningococcal septic shock patients compared to healthy controls or **(B)** meningococcal meningitis patients compared to the healthy control group using IPA. Significantly enriched canonical pathways were identified with a right-tailed Fisher’s Exact Test (p < 0.05), after correction for multiple testing using the Benjamini-Hochberg method). Transcripts included in the analysis were restricted to p < 0.05 and FC ≥| ± 1.5|. The Z-score=| ± 2| indicates predicted activation state of canonical pathway. Blue color or lighter shades of blue indicate a negative Z-score and down-regulation of the pathway, and orange or lighter shades of orange indicate a positive Z-score and up-regulation of the pathway. Gray color indicates no activity pattern available. Ratio denotes the number of significantly expressed transcripts compared with the total number of transcripts associated with the canonical pathway. More details for **(A)** are available in **Supplementary File 3**. More details for **(B)** are available in **Supplementary File 6**.

Analysis of the top enriched canonical pathways further highlighted a marked activation in the meningococcal septic shock group. (**Figure 4A**, **Supplementary File 3**). Significantly activated pathways included: S-100 family signaling pathway, oxidative phosphorylation, TREM1 signaling, neuroinflammation signaling, dendritic cell maturation, production of nitric oxygen species in macrophages, immunogenic cell death signaling pathway, HMGB1 signaling, pyroptosis signaling pathway, pathogen induced cytokine storm signaling pathway, iNOS signaling, IL-8 signaling and IL-15 signaling. Conversely, gene transcript levels were significantly down-regulated in pathways related to mitochondrial dysfunction and LXR/RXR activation.

**Figure 5:**
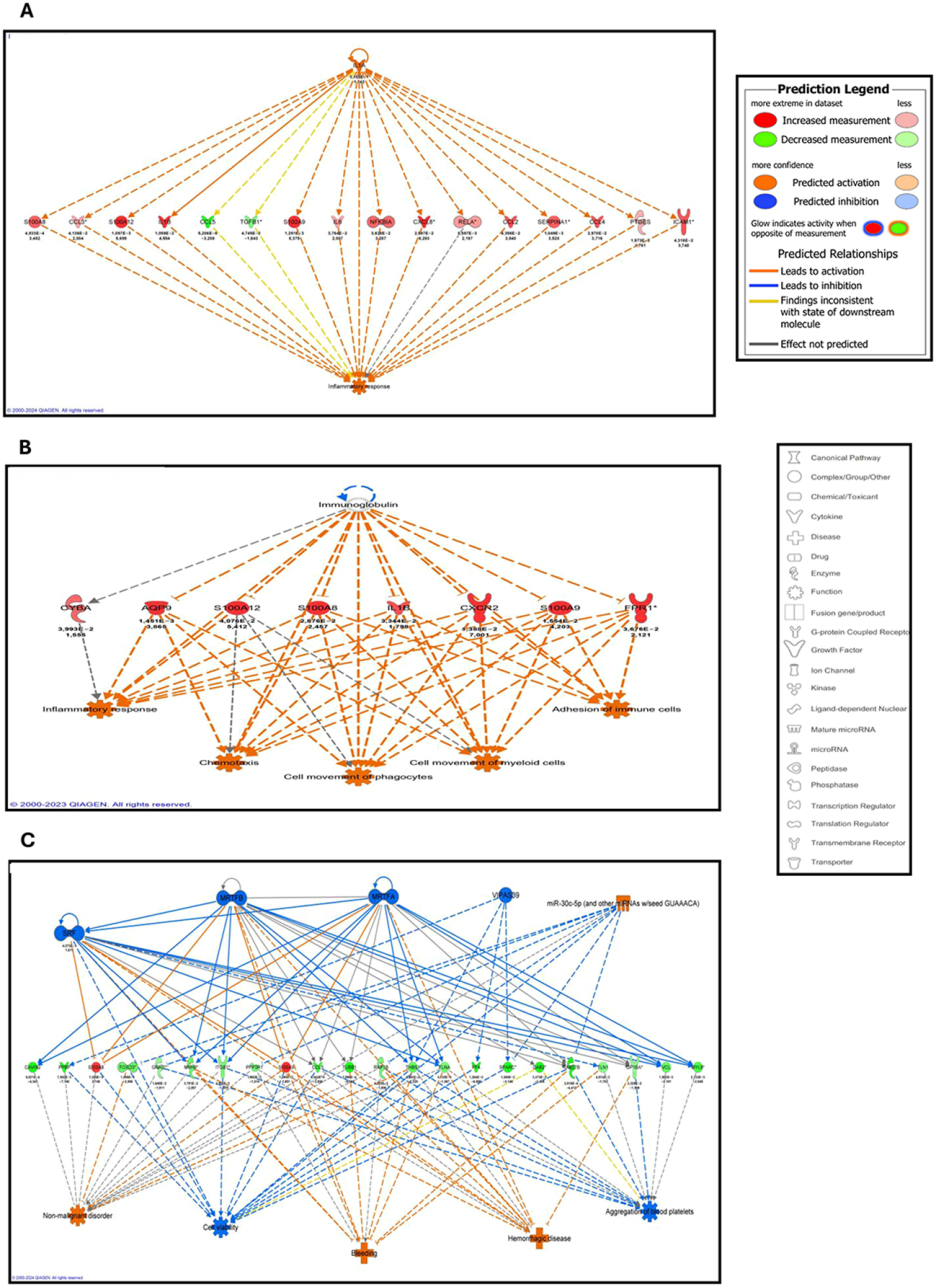
**(A–C)** Regulator effect networks with the highest consistency score of differentially expressed (DE) genes **(A)** between meningococcal septic shock patients and healthy controls or **(B)** between meningococcal meningitis patients and healthy controls or **(C)** between systemic pneumococcal disease patients and healthy controls predicted by Ingenuity Pathway analysis. Upstream regulators are located at the top of the network, target genes are in the middle of network and predicted biofunction in the bottom. P-values and fold changes of DE genes in our data set are showed underneath each gene (red color symbol indicates increased expression and green color decreased expression). Predicted activation have red color symbol and predicted inhibition have blue color symbols. More details for **(A)** are available in **Supplementary Files 4**, **5**. More details for **(B)** are available in **Supplementary Files 7**, **8**. More details for **(C)** are available in **Supplementary Files 10**, **11**.

The regulator network analysis comparing meningococcal septic shock patients to healthy controls, IL1A was identified as the top regulator with a consistency score of 3.75 (**Supplementary File 4**). In line with IL1A`s known proinflammatory role, levels of several downstream target genes were increased in our data set, including CCL2, CCL3, CCL4, CXCL8, ICAM 1, IL1A, IL1B, IL6, NFKB1A, PRGES, RELA, S100A12, S100A8, S100A9, SERPINA1 all of which are associated with an enhanced inflammatory response (**Figure 5A**, **Supplementary File 5**). However, CCL5 and TGFB1 showed decreased expression, which is inconsistent with the expected state of downstream molecules in this regulatory network (**Figure 5A**, **Supplementary File 5**).

In the meningococcal meningitis patients, the most prominent canonical pathways significantly changed were the neuroinflammation signaling, and production of nitric oxide and reactive oxygen species in macrophages pathways (**Figure 4B**, **Supplementary File 6**).

Regulator network analysis identified immunoglobulin as the top regulator with consistency score of 10.61 (**Supplementary File 7**). According to the known function of this upstream regulator, inhibition of immunoglobulins is associated with increased levels of all its target genes in our data set; AQP9, CXCR2, CYBA, FPR1, IL1B, S100A12, S100A8, and S100A9, showed increased levels of RNAs. Increased levels of these RNAs predicted to promote activation of inflammatory responses as well as adhesion of immune cells, cell movement of myeloid cells and cell movement of phagocytes (**Figure 5B**) (**Supplementary File 8**). However, the specific effect of immunoglobulins on the up-regulated target gene CYBA and its predicted role on the inflammatory response are not clear.

**Figure 6:**
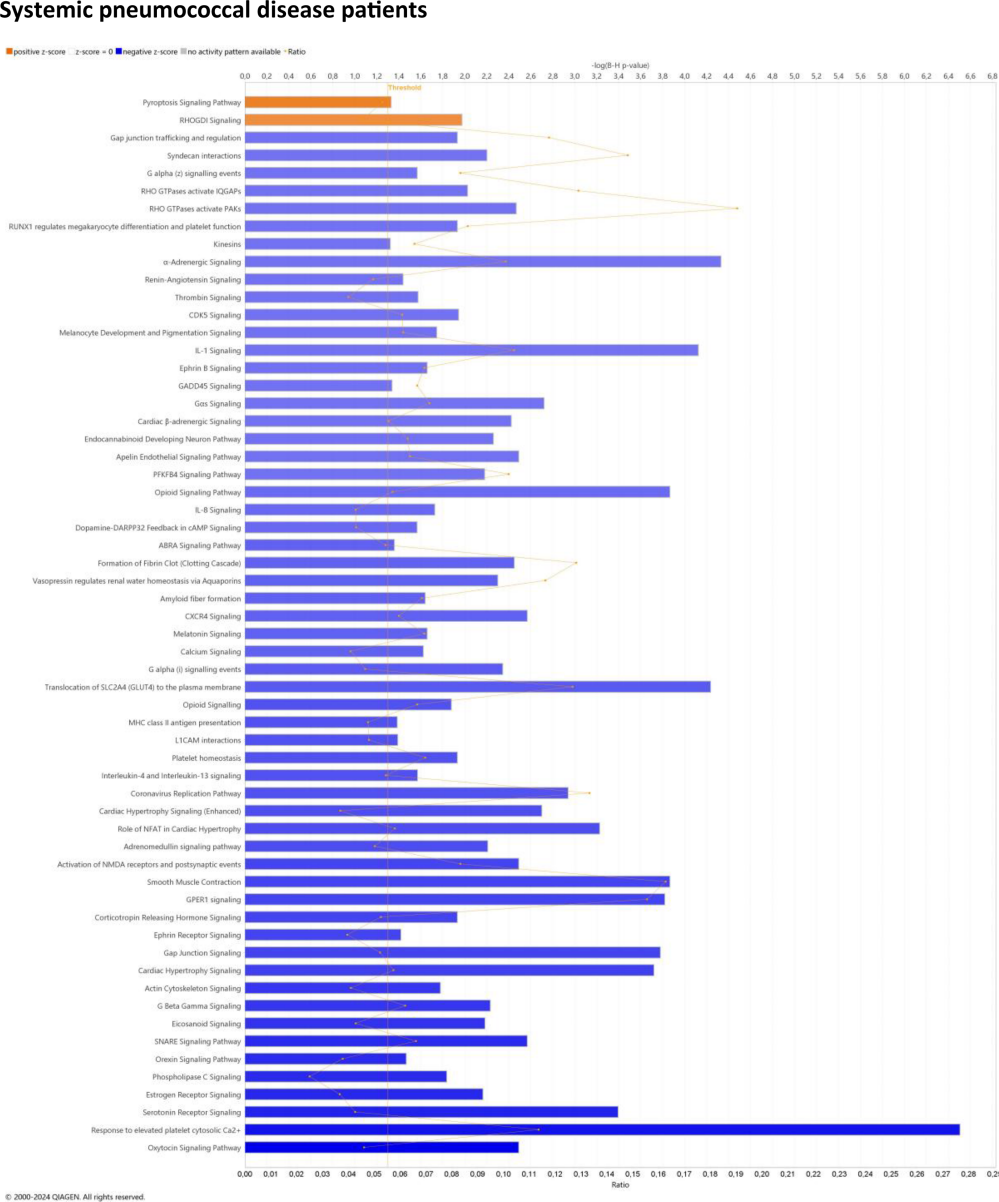
Predicted effects on canonical pathways based ≪core≫ analysis of the significant difference in EV-transcript patterns in the systemic pneumococcal disease patients compared to the healthy control group using IPA. Significantly enriched canonical pathways were identified with a right-tailed Fisher’s Exact Test (p < 0.05), after correction for multiple testing using the Benjamini-Hochberg method). Transcripts included in the analysis were restricted to p < 0.05 and FC ≥| ± 1.5|. The Z-score=| ± 2| indicates predicted activation state of canonical pathway. Blue color or lighter shades of blue indicate a negative Z-score and down-regulation of the pathway, and orange or lighter shades of orange indicate a positive Z-score and up-regulation of the pathway. Ratio denotes the number of significantly expressed transcripts compared with the total number of transcripts associated with the canonical pathway. More details for Figure 6 are available in **Supplementary File 9**.

The canonical pathways “core analysis” for systemic pneumococcal disease patients versus the healthy control group predicted an inhibition (**Figure 6**, **Supplementary File 9**), with highly reduced activation observed in the oxytocin signaling pathway, response to elevated platelet cytosolic Ca2+, serotonin receptor signaling, estrogen receptor signaling, and phospholipase C signaling. The only predicted up-regulated pathways were the pyroptosis signaling pathway and RHOGDA signaling.

Regulator network analysis identified five upstream regulators with consistency score of 13 (**Supplementary File 10**). Activation of these upstream regulators through the activated miR-30c-5p, combined with the inhibition of MRTFA, MRTFB, VIPAS39, and SRF, induce changes in the expression of target genes. Specifically, the expression of CAVIN2, CCL5, DAB2, FLNA, FOXO3, GNAI2, GP1BA, MYH9, MYL9, PF4, PPBP, PPP3R1, RAB27B, RAP1B, SPARC, THBS1, TUBB1, and VCL all showed decreased expression, while S100A8 and S100A9 showed increased expression. Such alterations may lead to decreased cell viability and platelet aggregation and predict an elevated risk of bleeding, hemorrhagic disorders, and non-malignant conditions (**Figure 5C**, **Supplementary File 11**).

### Comparison analysis of biofunctions, canonical pathways and upstream regulators between patient groups

**Figure 7:**
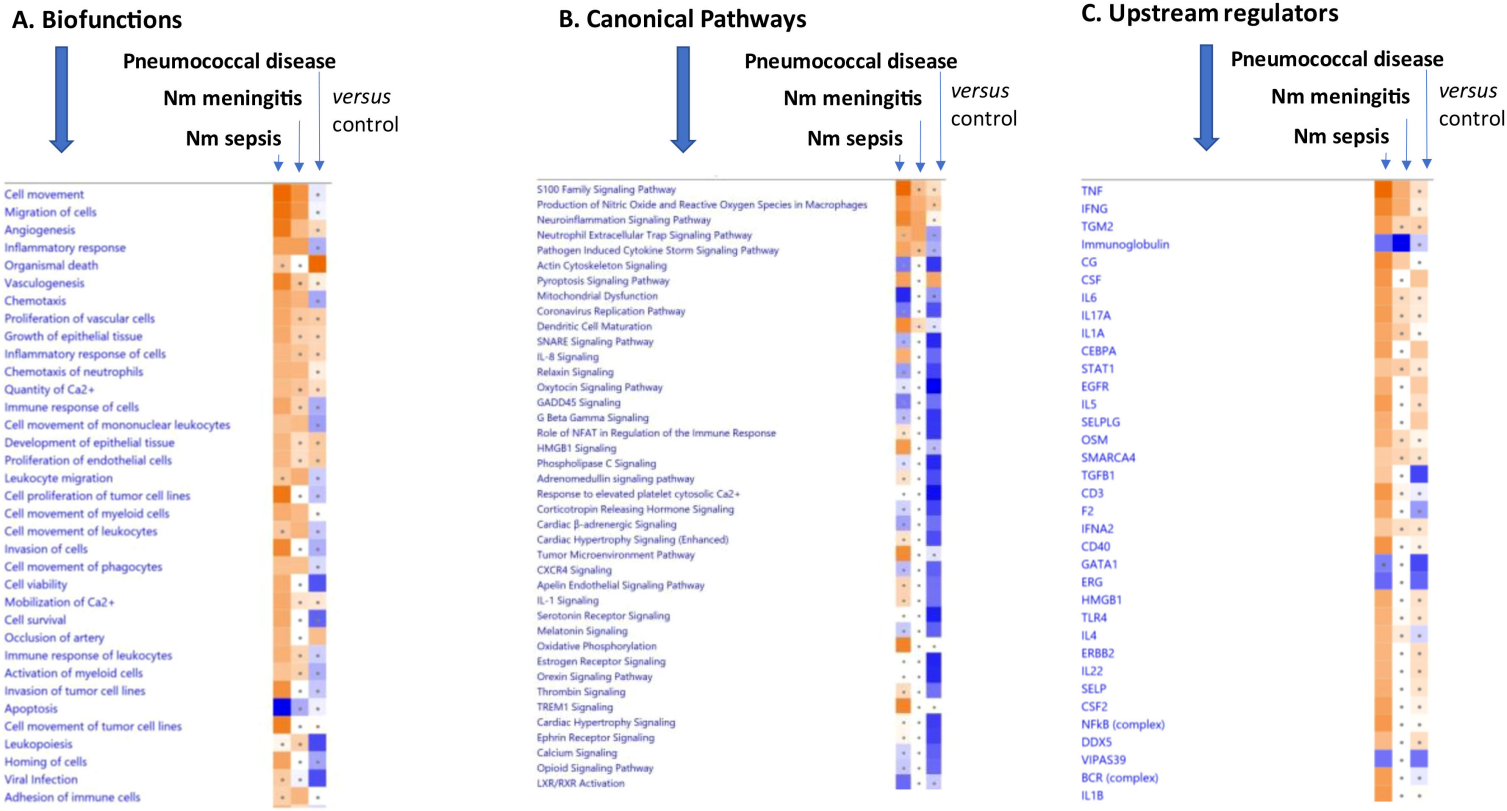
Predicted effects on biofunctions **(A)** canonical pathways **(B)**, and upstream regulators **(C)** based on significant differences in EV-transcript patterns in samples from patients with meningococcal septic shock (denoted Nm sepsis), meningococcal meningitis (denoted Nm meningitis) and systemic pneumococcal disease (denoted pneumococcal disease). ≪Comparison analysis≫ of biofunctions, canonical pathways, and upstream regulators significantly enriched in plasma EV samples from meningococcal septic shock patients vs. controls (healthy controls), meningococcal meningitis vs. controls and systemic pneumococcal disease patients vs. controls. The figure shows the most up-regulated biofunctions **(A)**, canonical pathways **(B)**, and upstream regulators **(C)** ranked according to expression levels in EV plasma samples in Nm Sepsis. The Z-score indicates predicted activation state of the biofunctions, canonical pathways, and upstream regulators. Z-score value >| ± 2| are displayed. A dot represents a Z-score value <| ± 2|. Blue color or lighter shades of blue indicate a negative Z-score and down-regulation of a biofunction, canonical pathways, and upstream regulators. Orange or lighter shades of orange indicates a positive Z-score and up-regulation of a biofunction, canonical pathways, and upstream regulators. White color indicates a Z-score as zero and no activity pattern identified. Note that only the top pathways are shown. (Filtering criteria: FC ≥| ± 1.5|, p< 0.05). More details for **(A)** are available in **Supplementary Files 12**, **15**. More details for **(B)** are available in **Supplementary Files 13**, **16**. More details for **(C)** are available in **Supplementary Files 14**, **17**.

A “comparison analysis” between all the patient groups (**Figures 7A–C**; **Supplementary Files 12**–**14**) show the top 35 biofunctions, top 46 canonical pathways and 35 upstream regulators, respectively. In this analysis a distinct difference in patterns between each patient group was observed. The predicted biofunctions included angiogenesis, vasculogenesis, proliferation of vascular cells and growth of epithelial tissue (**Figure 7A**, **Supplementary File 12**). However, in the meningococcal septic shock and meningococcal meningitis groups, the activated top biofunctions were predicted to be cell movement and migration of cells, with a substantially weaker response in the meningococcal meningitis group. An opposite prediction, inhibition, was observed in the systemic pneumococcal disease group. In all three groups the main biofunction to be inhibited was apoptosis (**Figure 7A**, **Supplementary File 12**).

The top activated canonical pathways in all three patient groups were predicted to be the S100 family signaling pathway, as well as production of nitric oxide and reactive oxygen species in macrophages, and the neuroinflammation signaling pathway (**Figure 7B**, **Supplementary File 13**). The pyroptosis signaling pathway was activated in both the meningococcal septic shock and systemic pneumococcal disease groups. The canonical pathways “actin cytoskeleton signaling”, and “mitochondrial dysfunction” were among the most inhibited in both the meningococcal septic shock and the systemic pneumococcal disease groups (**Figure 7B**, **Supplementary File 13**). The neutrophil extracellular trap signaling pathway, pathogen induced cytokine storm signaling pathway, dendritic cell maturation, IL-8 signaling, role of NFAT in regulation of the immune response, HMBG1 signaling, IL-1 signaling, thrombin signaling, and TREM signaling were predicted to be activated in the meningococcal septic shock and meningococcal meningitis group, however, they were reduced in the systemic pneumococcal disease group (**Figure 7B**, **Supplementary File 13**). Most of the elevated activation observed in the meningococcal septic shock group, showed a weaker response in the meningococcal meningitis group.

Among the transcripts in the predicted top up-regulated biofunctions and canonical pathways S100A12, S100A9 and S100A8 (**Supplementary Files 15**, **16**) again ranked at the top for all patient groups. Also, NCF2 and IL1B were abundant in all patient groups.

High levels of metalloproteinase 8 (MMP8) mRNA were observed in EVs from meningococcal septic shock patients, but not in those with meningococcal meningitis or systemic pneumococcal disease. This level of MMP8 mRNA predicted activation of several biofunctions, canonical pathways, and upstream regulators in meningococcal septic shock patients (**Supplementary Files 15**–**17**).

Actin cytoskeleton signaling and mitochondrial dysfunction pathways were the most inhibited canonical pathways in both meningococcal septic shock and in systemic pneumococcal disease patients (**Figure 7B**, **Supplementary File 13**). The oxytocin signaling pathway was the most inhibited pathway in patients with systemic pneumococcal disease, with a weaker inhibition observed in meningococcal septic shock patients (**Figure 7B**, **Supplementary File 13**). Additionally, in systemic pneumococcal disease patients, pathways such as response to elevated platelets cytosolic Ca2+, serotonin receptor signaling, estrogen receptor signaling, and phospholipase C signaling were also among the most inhibited (**Figure 7B**, **Supplementary File 13**).

In all three patient groups, the top activated upstream regulators were TNF, IFNG and TGM2, while immunoglobulin was the most inhibited (**Figure 7C**, **Supplementary File 14**). Most of the activities predicted by elevated upstream regulators were detected in the meningococcal septic shock group, whereas weaker responses were predicted in the meningococcal meningitis- and in the systemic pneumococcal disease groups. Consistently, the gene heatmaps (**Supplementary File 17**) predicted the top transcripts as the most activated upstream regulators to be S100A12, S100A8, S100A9 and AQP9.

### Validation of RNA profiles with RT-qPCR

The microarray findings were confirmed through RT- qPCR analysis for genes exhibiting significantly elevated levels across the different patient groups. To assess the consistency between the microarray and RT-qPCR results, we calculated the correlation coefficients based on the fold changes in each patient group compared to the healthy control group. The correlations were strong for the following genes: S100A12 (r = 0.984), S100A9 (r = 0.999), S100A8 (r = 0.989), and AQP9 (r = 0.999).

### Calprotectin measurements at admission to hospital

Due to the highly expressed S100A8/A9 transcripts in all three patient groups in our study, the protein calprotectin in plasmas was quantified.

**Figure 8:**
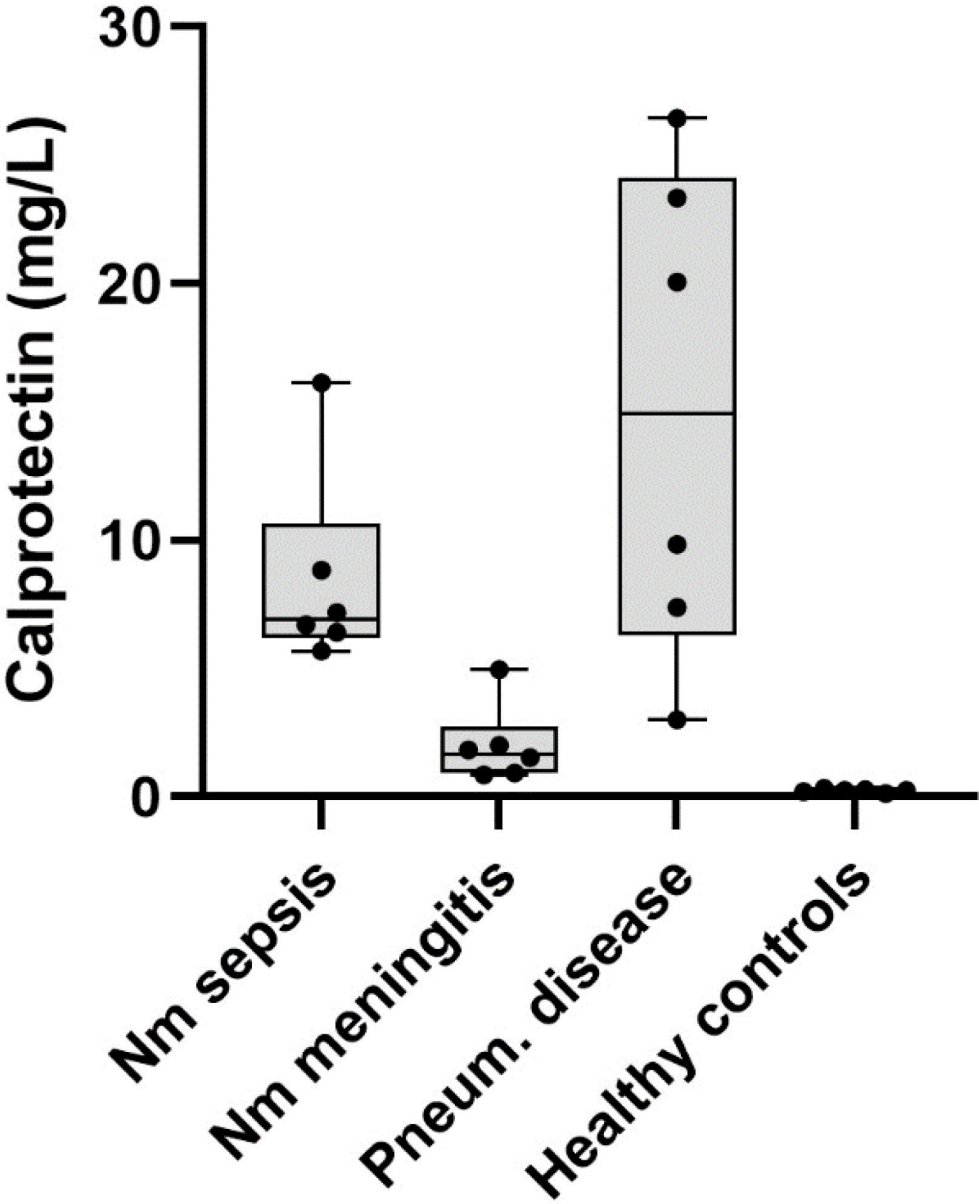
Concentrations (mg/L) of calprotectin in EDTA plasmas from meningococcal septic shock-, meningococcal meningitis-, systemic pneumococcal disease patients and healthy controls. The GraphPad Prism figure shows box and whiskers graphs with median value and individually samples in each group. Nm sepsis = *Neisseria meningitidis (N. meningitidis)* septic shock patients. Nm meningitis = *Neisseria meningitidis (N. meningitidis)* meningitis patients Pneum. disease = *Streptococcus pneumoniae (S. pneumoniae)* Systemic pneumococcal disease patients.

The median concentration of calprotectin in the meningococcal septic shock group was 6.94 mg/L (range 5.69-16.14); in the meningococcal meningitis group 1.69 mg/L (range 0.85-4.95); and in the systemic pneumococcal disease group 14.94 mg/L (range 3.01-26.44). In the healthy control group, the median concentration was 0.26 mg/L (range 0.14-0.28) (**Figure 8**).

### Comparison of RNA profiles in plasma EVs and tissue samples from meningococcal septic shock patients

**Supplementary Files 1A, B** show the top 50 gene transcripts with significantly higher or lower signal levels in plasma EVs from patients with meningococcal septic shock compared to healthy controls. Comparing these results with non-coding RNA profiles in cells from large organs from meningococcal septic shock patients (Table 3 in Brusletto et.al (Brusletto et al., 2020),), the results indicate that non-coding RNAs have the highest level of fold-change-level in both studies.

High levels of piRNAs were found both in plasma EVs (**Supplementary Files 1A, B**) and in tissue samples such as kidneys, liver, and spleen (Brusletto et al., 2020). In contrast, mRNAs and lncRNAs exhibited the highest levels in lung and heart tissues. Among the RNA transcripts with decreased levels, microRNAs, mRNAs, and lncRNAs were the most affected in plasma EVs. Meanwhile, in tissue samples, tRNAs were the most affected in lungs, heart, liver, and spleen tissues, while piRNAs were the most impacted in kidneys.

**Figure 9:**
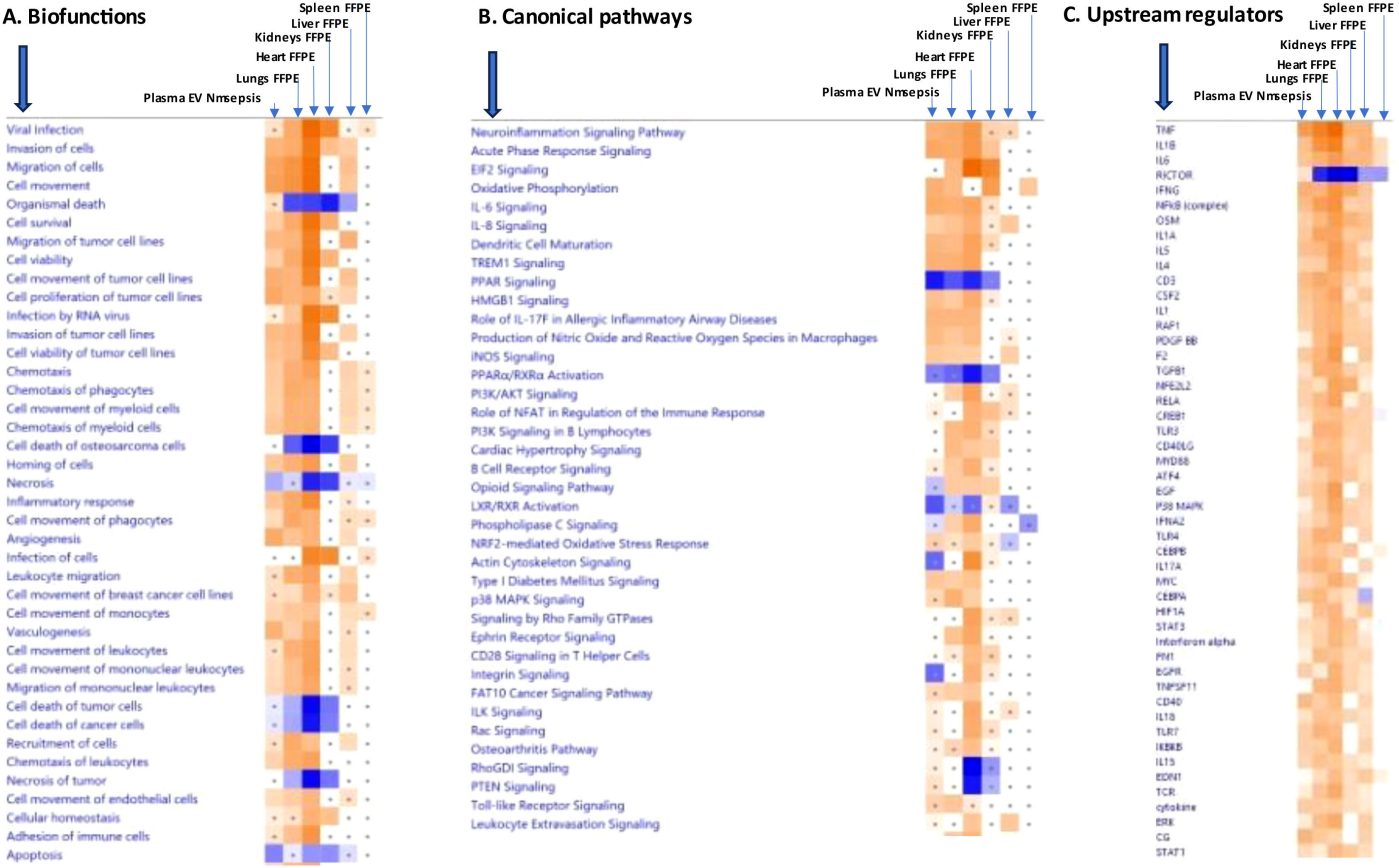
Predicted effects on biofunctions **(A)**, canonical pathways **(B)**, and upstream regulators **(C)** based on EV-RNA patterns in plasma samples from patients with meningococcal septic shock (denoted Plasma EV Nm sepsis) vs. healthy controls, and in Formalin-Fixed Paraffin-Embedded (FFPE) tissue samples such as Lungs, Heart, Kidneys, Liver and Spleen from patients with meningococcal septic shock vs. control patients (acute non-infectious death). ≪Comparison analysis≫ (FC ≥ |1.5| and p-value < 0.05) of biofunctions, canonical pathways, and upstream regulators significantly enriched in plasma EV samples vs. controls (healthy controls and in tissue samples vs. controls. The figure shows the most up-regulated biofunctions **(A)**, canonical pathways **(B)**, and upstream regulators **(C)** ranked according to expression levels in EV plasma samples in Nm. Sepsis. The Z-score indicates predicted activation state of the biofunctions, canonical pathways, and upstream regulators. Z-score value >| ± 2| are displayed. A dot represents a Z-score value <| ± 2|. Blue color or lighter shades of blue indicate a negative Z-score and down-regulation of a biofunction, canonical pathways, and upstream regulators. Orange or lighter shades of orange indicates a positive Z-score and up-regulation of a biofunction, canonical pathways, and upstream regulators. White color indicates a Z-score as zero and no activity pattern identified. Note that only the top pathways are shown. (Filtering criteria: FC ≥| ± 1.5|, p< 0.05). More details for **(B)** are available in **Supplementary File 18**.

A “comparison analysis” of mRNA transcripts by IPA of top biofunctions, canonical pathways and upstream regulators in plasma EVs and FFPE tissue samples from lungs, heart, kidneys, liver, and spleen is shown in **Figures 9A–C**. This analysis shows a high degree of similarity between mRNA transcripts from plasma EVs and in tissue samples from meningococcal septic shock patients.

The IPA “comparison analysis” predicted the top regulated biofunctions in the plasma EVs and in all five organ tissues to be viral infection, invasion of cells, migration of cells, cell movement, cell survival, and cell viability. The predicted biofunctions mirror each other, however, with varying levels (**Figure 9A**). The biofunction organismal death showed opposite pattern with increased regulation in plasma EVs and decreased regulation in the tissue samples.

Also, the “comparison analysis” regarding canonical pathways showed a remarkably similar pattern (**Figure 9B**, **Supplementary File 18**). The top activated canonical pathways in plasma EVs and in the tissue samples from the meningococcal septic shock patients were predicted to be the neuroinflammation signaling pathway, acute phase response signaling, oxidative phosphorylation, IL-6 signaling, IL-8 signaling, dendritic cell maturation, TREM signaling, HMGB1-signaling, role of IL-17 in allergic inflammatory airway diseases, and production of nitric oxide and reactive oxygen species. Several of the same pathways were also predicted to be down-regulated, such as PPAR signaling, PPARα/RXRα activation, and LXR/RXR activation. A different transcription pattern was found for EIF2 signaling, which is not present in the plasma EV. However, EIF2 signaling was predicted highly up-regulated in tissue samples such as lungs, heart and kidneys. Actin cytoskeleton signaling, integrin signaling and mitochondria dysfunction pathways (further down on the list, see **Supplementary File 18**) were all predicted to have a reduced activity in plasma EV based on the RNA pattern.

In the “comparison analysis” of top up-regulated upstream regulators, TNF, IL-1B and IL-6 exhibited an almost identical pattern (**Figure 9C**). In contrast, RICTOR displayed an opposite pattern, showing a slight increase in plasma EV-RNA but a strong down-regulation in all the tissue samples.

## Discussion

Our findings indicate that the RNA-profiles in well characterized EVs isolated from EDTA-plasma upon hospital admission in patients with severe sepsis due to *N. meningitidis* significantly deviate from those of healthy controls. Interestingly, these profiles exhibit both similarities and significant differences when compared to RNA profiles in EVs from patients with severe systemic disease caused by *S. pneumoniae*. We found significantly higher levels of coding and non-coding RNAs in plasma-EVs from meningococcal septic shock patients and systemic pneumococcal patients than from meningococcal meningitis patients. Importantly, the mRNA content observed previously in postmortem examination of cells from large organs of patients with lethal *N. meningitidis* septic shock was reflected in the content of mRNA in plasma-EVs isolated from patients with severe meningococcal septic shock. High levels of EV-mRNAs for the antibacterial protein Calprotectin (S100A8/A9) corresponded with high, but varying, plasma levels of Calprotectin in all three groups of patients.

Due to the large numbers of RNAs showing significant differences, only a selection of those with the highest fold changes and most pronounced differences are presented in majority of figures and tables.

Among a myriad of large differences observed in transcript type and levels between groups, a key finding in our study was the transcripts associated with the S 100 family signaling pathway and the AQP9 gene transcripts which were significantly altered across all patient groups. These results were further validated by RT-qPCR, which demonstrated correlation coefficients between the RT-qPCR and microarray results ranging from 0.90-0.99.

### Distribution of EV-RNA subtypes from patients infected with *N. meningitidis* or *S. pneumoniae*

Various EV-RNA subtypes were observed in plasma from all the patient groups and healthy controls depending on pathogen and infection site (**Figure 3C**). The most abundant subtypes across all groups were mRNA and several non-coding RNAs (piRNA, lncRNA, lincRNA, tRNA, miRNA, YRNA and snoRNA) (**Figure 3A**). These findings support other studies on sepsis that have detected various non-coding RNAs in EVs (O'Brien et al., 2020) (**Figure 3C**). Meningococcal patients diagnosed with septic shock, all of whom had high levels of *N. meningitidis* in their circulation, showed more than a tenfold increase in the number of RNA transcripts compared with the meningitis group (**Figure 3C**). This observation suggests that increasing number of *N. meningitidis* and levels of LPS in plasma trigger activation of an increasing number of both coding and non-coding genes.

Both differences and similarities of the EV-RNA transcript levels were observed when comparing the *N. meningitidis* and *S. pneumoniae* patient groups (**Figure 3C**). In the meningococcal septic shock group 3,911 specific RNA transcripts showed altered RNA levels compared to that of healthy control, while in the meningococcal meningitis group 147 showed altered RNA levels. In contrast, the pneumococcal patients exhibited changes in 416 RNA transcripts. These transcriptomic differences are likely attributable to the distinct pathogenic mechanisms of the two bacteria, particularly the toxins they produce: Lipopolysaccharide (LPS) in *N. meningitidis* (DeVoe, 1982; Brandtzaeg et al., 2001a) and pneumolysin in *S. pneumoniae* (Engholm et al., 2017). The meningococcal patients were separated into meningococcal septic shock or meningococcal meningitis based on their clinical definitions (Brandtzaeg et al., 1989). All patients with systemic pneumococcal infection had confirmed *S. pneumoniae* either in blood culture or cerebrospinal fluid (CSF). Furthermore, *S. pneumoniae* was detected in the plasma of five out of six patients using digital droplet PCR (ddPCR) (**Table 1**), suggesting varying degrees of disease severity. However, the heterogeneity of this group may have reduced signal strength and lead to the exclusion of certain RNA transcripts under the applied criteria, but statistically significant differences were shown compared to the healthy controls.

Among the top 50 RNA subtype transcripts with significantly increased levels (**Supplementary File 1**), similar piRNA transcripts were identified in both the meningococcal septic shock and *S. pneumoniae* patient groups, specifically piRNA-32972 and piRNA-44172. These specific piRNA transcripts have not been reported in previous studies, leaving their function unknown. Recent research has shown that piRNAs play crucial roles in disease progression in human conditions such as cancer, pulmonary/cardiovascular diseases, infectious diseases and reproductive disorder, though their precise roles in these contexts remain largely unclear (Goh et al., 2022; Jiang et al., 2024). Our results confirm that they are involved in serious human infections.

Among the top 50 RNA subtypes transcripts with significantly decreased levels (**Supplementary File 1**) several were similar in both the meningococcal septic shock group and the systemic pneumococcal disease group. LncRNAs, known for their role in regulation of the inflammatory response, may act as “sponges” for different miRNAs or compete with endogenous RNAs, thereby modulating the expression (Bayoumi et al., 2016). In our study, EV- lncRNAs were present with high levels in all patient groups (**Supplementary File 1**). This raises the question: could these expressed EV- lncRNAs indicate a state of immunosuppression, particularly in the systemic pneumococcal disease group (Littmann et al., 2009; Wang et al., 2021) ? Immunosuppression often occurs in the later stage of sepsis, due to suppression of T-cell activation and Interferon gamma (IFN- ϒ) production. LncRNAs have been identified to be significantly involved in the pathophysiology of sepsis by Lu et al (Lu et al., 2021).

Also, several long-intergenic non-coding RNAs (lincRNAs) were detected (**Figure 3C**, **Supplementary File 1**). These lincRNAs are known to be transcriptionally activated through mechanisms similar to mRNAs and exhibit a tissue-specific and stable expression compared to other types of lncRNAs (Cabili et al., 2011; Arunima et al., 2023). We also observed increased levels of Y-RNA in our patient groups; this non-coding RNA has been associated with inflammation and immune suppression (Driedonks and Nolte-'t Hoen, 2018).

### Predicted biofunctions, canonical pathways, upstream regulators and regulatory effects of EV-RNAs from patients infected with *N. meningitidis* or *S. pneumoniae*

A significant number of activated biofunctions were predicted based on the different EV-RNA profiles in our patient groups, as shown in (**Supplementary Files 2A–C**). Pathway analysis demonstrated strong activation of inflammatory and immune signaling in meningococcal septic shock, weaker activation in meningitis, and inhibition of multiple pathways in pneumococcal infection. Many of these biofunctions have been observed in previous studies by our group and others (Iba and Levy, 2018; Brusletto et al., 2020). Some similarities between pathogens were also noted, but the most striking findings were the highly significant differences between groups. The inhibition observed in the systemic pneumococcal group, to the best of our knowledge, has not been previously reported. Further analysis of the data will help clarify the similarities and differences more comprehensively.

The top canonical pathways predicted in the mRNA pool across all three patient groups, included the S100 family signaling pathway, production of nitric oxide and reactive oxygen species in macrophages, and neuroinflammation signaling pathway given elevated levels of S100 A8, S100 A9, and S100 A12 in all patient groups (**Table 2**; **Figures 4**, **6**, **7**; **Supplementary Files 3**, **9**, **13**, **15**–**17**). The heterodimer of S100A8/A9, known as calprotectin, is highly expressed by activated immunocytes such as neutrophils, monocytes, and macrophages during inflammation and bacterial infection, exhibiting anti-bacterial and pro-inflammatory properties (Johne et al., 1997; Vogl et al., 2007; Meijer et al., 2012; Pruenster et al., 2016). Calprotectin has been evaluated as a biomarker for sepsis in several studies (Bartáková et al., 2019; Dubois et al., 2019; Lipcsey et al., 2019; Christensen et al., 2022) though with varying success across different patient groups. Our analysis of calprotectin levels in plasma from our patient groups and controls (**Figure 8**) clearly separated the meningococcal septic shock and meningococcal meningitis groups. Interestingly, the group with systemic pneumococcal disease showed calprotectin levels that were twice as high as those observed in the meningococcal septic shock group consistent with findings from other studies (Christensen et al., 2022).

In all patient groups, the AQP9 transcript (**Table 2**; **Supplementary Files 15**–**17**) was highly expressed. Aquaporins play essential roles in regulating metabolic and redox homeostasis, as well as in mediating cell migration, processes that are critical in inflammatory responses and sepsis (Matsushima et al., 2014; da Silva et al., 2022; Lotsios et al., 2023). Our findings indicate that the EV-RNA profiles of AQP9 detected in plasma reflect a substantial activation of AQP9 during systemic meningococcal and pneumococcal disease. This heightened cellular activation may have influenced the transmembrane water transport and modulated the inflammatory response.

IL1B mRNA was present with high levels in all patient groups’ pathways (**Figure 7**; **Supplementary Files 12**–**17**). This protein plays a central role as released as a product of inflammasome activation, which regulates the inflammatory response to pathogens and stress signals (Martinon et al., 2002; Broz and Dixit, 2016). When activated, the inflammasome facilitates the activation of caspase-1, which leads to cleavage of IL1B, IL18 and gasdermins into mature proteins. These cytokines are important for recruiting immune cells to the site of infection and gasdermins will orchestrate pyroptosis (Broz and Dixit, 2016; Broz, 2019). Another important outcome of inflammasome activation is the release of extracellular vesicles (MacKenzie et al., 2001; Zhang et al., 2017; Budden et al., 2021).

Of great interest is that several canonical pathways were predicted to be activated in the meningococcal septic shock and meningococcal meningitis groups while reduced in the systemic pneumococcal disease group (**Figures 4**, **6**, **7B**; **Supplementary File 13**). The activation of these pathways contributes to a hyper-inflammatory response characterized by a cytokine storm induced by the immune cells reacting to the pathogens. While this cytokine storm may help control infections, they may also overact and cause severe tissue damage and contribute to multiple organ failure (Hotchkiss et al., 2013). Sepsis studies have demonstrated that EVs released into the circulation can transport damage-associated molecular patterns (DAMPs) to distant host cells, thereby initiating inflammatory cascades of cell death, increasing endothelial permeability and NET Formation, generating secretion of pro-inflammatory factors, promoting macrophage proliferation and M1 polarization (Nair et al., 2018; Jiao et al., 2020; Tian et al., 2022). In our study, EVs containing DAMPs such as HMBG1 were detected across all patients’ groups. Several studies are also linked to endothelial cell derived EVs and disseminated intravascular coagulation (DIC) (Delabranche et al., 2013; Alhamdi and Toh, 2016). Worth noting, during sepsis activated endothelial cells enhance their procoagulant activity by producing EVs capable of binding to neutrophils (Ogura et al., 2004). These regulatory effects, which were found affected in our study, are mainly dependent on the cargo of EVs (Gao et al., 2019; Jiang et al., 2019).

The matrix metalloproteinase 8 (MMP8) transcript was highly increased in the meningococcal septic shock patient group, but not in meningococcal meningitis and systemic pneumococcal disease (**Supplementary Files 15**–**17**). EV-associated MMPs have been shown to regulate extracellular matrix (ECM) remodeling and mediating shedding of receptors located either at EV membranes or at the surface of targeted cells. Through these mechanisms, MMPs may contribute to regulate membrane dependent signaling between EVs and recipient cells, thereby altering intercellular communication (Shimoda, 2019). Certain MMPs, such as MMP3, can also be delivered via EV to recipient cells, where they act intracellularly to modulate signaling pathways through transcriptions factors (Okusha et al., 2020). In sepsis, elevated MMP8 levels have been associated with poor prognosis, contributing to the inflammatory response and leukocyte adhesion, which may exacerbate sepsis and disease progression (Fang et al., 2022).

The most inhibited canonical pathways were predicted to be the actin cytoskeleton signaling and the mitochondrial dysfunction in all patient groups (**Figure 4A**, **Supplementary File 3**) (**Figure 6**, **Supplementary File 9**) (**Figure 7B**, **Supplementary File 13**). Disruption of actin cytoskeleton dynamics has been linked to severe pathophysiological conditions observed in sepsis, such as vascular leakage and impaired leukocyte function (Carpenter, 2000). Multiple microorganisms may manipulate the host cytoskeleton by remodeling actin filaments and microtubules to facilitate invasion or to escape host defenses (Wen et al., 2020). The actin cytoskeleton may also be involved in EV biogenesis and release, and alterations in the cytoskeletal dynamics during sepsis can influence EV production and release (Holliday et al., 2019). Similarly, EVs carry signaling molecules that can modulate actin cytoskeleton dynamics in recipient cells, further perpetuating signaling cascades involved in sepsis (Javier-Reyna et al., 2023).

In the context of sepsis, mitochondrial dysfunction has significant consequences, including energy deficiencies and shaping of the inflammatory response through various signaling pathways (Marchi et al., 2023; Hu et al., 2024). Recently, the immunoregulatory effects of mitochondria from EVs were investigated. These studies indicated that the mitochondria contribute to immune regulation and exert immunoregulatory effects upon delivery by EVs (Islam et al., 2012; She et al., 2021). In our study, the transcript encoding MT-CYB (mitochondrially encoded cytochrome b), a component of the mitochondrial respiratory chain, showed the most significant fold-change (FC) reduction (**Table 2**) in EVs from patients with meningococcal septic shock and systemic pneumococcal disease (**Supplementary Files 1B, F**; **Table 2**).

In the meningococcal septic shock and systemic pneumococcal disease groups, our results showed a reduced level of platelet transcripts such as PPBP (pro-platelet basic protein), PF4 (platelet factor 4), and TUBB1 (Tubulin, Beta 1 Class VI) (**Supplementary Files 1B, F**; **Table 2**; **Figure 5C**; **Supplementary File 11**). Platelets are crucial in the body’s immune response, especially during infections by managing inflammation, coagulation, and interacting with pathogens. Thrombocytopenia, which is common in sepsis, worsens prognosis due to its association with bleeding and organ failure (Shannon, 2021). Activated platelets and their derived EVs are involved in inflammation and coagulation (Hellum et al., 2014), with EVs promoting excessive NET formation and procoagulant activity, further complicating sepsis progression (Iba and Ogura, 2018; Jiao et al., 2020; Kerris et al., 2020). Our study indicates that the reduced level of these transcripts may contribute to worsen the disease outcome due to consumption of platelets at the disease stage upon admission to hospital.

Hormonal pathway alterations are common in sepsis patients as the body responds to the intense stress, and play a protective role by acting as part of the body’s anti-inflammatory response (Widmer and Schuetz, 2018). In the patient groups studied, several hormonal pathways were affected (**Figure 6**; **Supplementary File 9**; **Figure 7B**; **Supplementary File 13**). These pathways involve hormones like oxytocin, serotonin, and estrogens, along with enzymes such as phospholipase C, all of which have shown potential in limiting sepsis and preventing organ damage (Walker and Watson, 1993; Pelekanou et al., 2016; Trenti et al., 2018; Wu et al., 2019; Harding and Heaton, 2022; Mehdi et al., 2022). Together, the inhibition of these pathways observed in this study suggests that, at an early stage of sepsis, the body may activate defense mechanisms through hormonal pathways.

Regulatory effects of the RNA profiles in EVs in plasma from the different bacteria, predicted by IPA, showed that the inflammatory response and the S100 family signaling pathway were the far most dominant in patients with systemic meningococcal disease (**Figures 5A, B**; **Supplementary Files 4**, **5**, **7**, **8**). In patients with systemic pneumococcal disease, the regulatory effect analysis (**Figure 5C**; **Supplementary Files 10**, **11**) also showed significant changes in serum response factor (SRF), several nucleus transcription factors (MRTFB, MRTFA), and microRNAs (miR-30c-5p) which all may affect cell viability and bleeding disorders, in addition to activation of the inflammatory response and the S100 family. The significant difference between these two pathogens (**Figure 5**) is probably due to the distinct EV cargo that mainly arises from LPS in the meningococcal group and pneumolysin in pneumococcal group. However, this unique pathophysiology between the bacteria should be further explored.

A highly significant finding when comparing meningococcal and pneumococcal patient groups was the pronounced pro-inflammatory response in plasma EVs from meningococcal septic shock patients, in contrast with an overall immunosuppression observed in patients with systemic pneumococcal disease. Codemo et al. demonstrate that vesicles released by the pneumococci have a cargo enriched of the major cytotoxin pneumolysin (Codemo et al., 2018). During early stages of pneumococcal infection, these vesicles may be internalized in the lower airways and contribute to the symptoms of pneumococcal invasive disease. Additionally, this may be related to the distinct molecules in EVs from *S. pneumoniae* patients that are absent in *N. meningitidis* patients (Engholm et al., 2017; Codemo et al., 2018). This difference might reflect variations in the disease progression at the time of hospital admission between meningococcal and pneumococcal patient groups. The imbalance between inflammatory responses and immunosuppression is crucial in the onset and progression of sepsis (van der Poll et al., 2021). Extracellular vesicles may help regulate this balance. However, there is limited research on the role of EVs in the immunosuppressive mechanisms of sepsis (Tian et al., 2022).

Our group and others have previously studied major cytokines in the circulation of patients infected by *N. meningtidis* and *S. pneumoniae* (Brandtzaeg et al., 2001a; Linder et al., 2020). We found a significant difference in the cytokine profiles of patients infected with these two bacteria (Bjerre et al., 2004). In addition, a collaboration study of cerebrospinal fluid (CSF) from Ethiopian patients with meningitis caused by *N. meningitidis* or *S. pneumoniae* revealed marked differences in the levels of 19 cytokines and other inflammatory mediators detected in CSF, indicating that the immune response varies significantly depending on the invading bacteria (Mihret et al., 2019). Specifically, nine of these inflammatory markers such as IFN -γ, MIP-1α, MMP-9, MCP-1, MIP-1α, IL-4, IL-12 p70, IP-10, RANTES, and TRAIL showed significantly higher levels in CSF in the pneumococcal group compared with the meningococcal meningitis group (Mihret et al., 2019).

### Comparison of EV-RNAs from plasma and cellular RNAs from large organs in patients with meningococcal septic shock

We have in a previous study analyzed the RNA content in post-mortem formalin fixed, paraffin embedded tissue samples from patients with meningococcal septic shock, focusing on heart, lungs, kidneys, liver, and spleen (Brusletto et al., 2020). The transcription profiles revealed a complex pattern of both protein-coding and non-coding RNAs. Comparison of the mRNAs in these post-mortem tissue samples and EV-mRNAs in plasma from patients at hospital admission in the present study, showed that the transcriptomic patterns of the plasma EVs remarkably mirrored those found in the tissues across several predicted biofunctions, canonical pathways, and upstream regulators (**Figure 9**, **Supplementary File 18**). Regarding biofunctions related to cell death, necrosis, and apoptosis, several of them demonstrated significant differences between the post-mortem tissue samples and the EVs. In non-coding RNAs several similarities were found in the post-mortem tissue samples and the EVs. The top non-coding RNAs in both compartments with increased levels were piRNAs and lncRNAs, while the RNAs with largest decreased levels were microRNA and lncRNA only found in EVs from plasma. In contrast, tRNAs were the dominating non-coding RNAs in the tissue samples, showing decreased levels. These findings indicate that *N. meningitidis* activates various defense systems in the body at a very early stage of disease. Our results suggest that the RNA content detected in EVs in plasma, collected from patients with meningococcal septic shock at hospital admission, already reflects the cellular RNA profiles previously detected in tissue samples from large organs in post-mortem examination of *N. meningitidis* shock cases (Brusletto et al., 2020). This clearly shows a role of EVs in communication during sepsis.

### Strengths and limitations

Our study examined the EV-RNA profiles in plasma from patients infected with the *N. meningitidis* or *S pneumoniae*, cursive revealing significant differences between the patient groups and healthy controls. Patients with *N. meningitidis* infection were thoroughly examined and classified into two groups: sepsis or meningitis, based on whether they met the sepsis criteria. The systemic pneumococcal infection patients had all *S. pneumoniae* detected in their blood culture or in their cerebrospinal fluid, though likely with varying disease severity, probably including both meningitis and sepsis. A more detailed classification of the systemic *S. pneumoniae* infections could have provided further insights into the differences between the diseases. However, such information was unavailable from these old samples.

Our results demonstrate successful isolation for EVs from our plasma samples using size exclusion chromatography. However, we were unable to determine the presence of non-EV particles that are likely co-isolated in the EV-enriched fractions – an acknowledged challenge in EV research. Despite this limitation, we clearly confirmed the presence of cup-shaped vesicle through TEM, established EV markers (CD9, Hsc70/Hsp70, syntenin-1, and the negative marker calnexin) in our enriched samples through semi quantitative Western blot analysis.

EVs isolated from the plasma of patients infected with *N. meningitidis* and *S. pneumoniae* may contain bacterial EVs, which fall within the same size range (30–150 nm) as host-derived eukaryotic EVs. Consequently, the isolated RNA pool likely includes a mixture of RNA from both host and bacterial EVs, potentially influencing the transcriptomic data. However, our microarray analysis only included probes for detecting human RNA molecules. An analysis of the V3-V4 hypervariable regions of the 16S ribosomal RNA (rRNA), specific to each bacterium (Yang et al., 2020; Park et al., 2021; Effah et al., 2024), using next-generation sequencing could have provided a more comprehensive and accurate quantification and classification of the RNA profiles in these complex samples.

Proteins derived from selected mRNA transcripts, such as calprotectin, were validated using a turbidimetric method, while the presence of other proteins was predicted through the web-based software IPA, which analyzes and predicts molecular pathways and activities. This provided valuable insights into the regulatory effects of different pathogens on the human host. Analyzing the non-coding RNA pool from high throughput experiments to gain a complete understanding of its regulatory mechanisms and functions is still challenging, largely due to limited availability of software tools for non-coding RNA analysis in comparison to those available for mRNA. Future exploration of our non-coding RNA data is expected to offer a clearer understanding and interpretation of the changes occurring within this RNA pool during sepsis.

## Conclusions

The interaction between invading bacteria and the host’s immune system is complex, with extracellular vesicles (EVs) possibly playing a crucial role in modulating immune responses in different ways through intercellular communication. Our study demonstrates that *N. meningitidis* and *S. pneumoniae*, which typically invade humans by colonizing the nasopharynx, can trigger various host defense mechanisms in different ways, leading to the secretion of EVs with distinct RNA profiles into the bloodstream. Analysis of the plasma EV-RNA from patient groups infected with either *N. meningitidis* or *S. pneumoniae* showed both similarities and notably differences, primarily consisting of small non-coding RNAs. These RNAs are predominantly regulatory and may significantly influence the cellular responses, highlighting the need for further investigation into their roles. Key findings included significant changes in the EV mRNA load between the meningococcal and pneumococcal patient groups, leading to prediction of distinctly opposite activation of several canonical pathways.

Additionally, examining the RNA profiles in plasma EVs from patients with meningococcal meningitis compared to those with the meningococcal septic shock group revealed a dose-dependent response relationship between the load of meningococci/lipopolysaccharides (LPS) and the RNA profiles. This quantitative relationship in circulating EV-RNA has not been previously documented in meningococcal infections.

Notably, the RNA content in plasma EVs from meningococcal patients at hospital admission closely resembled the cellular RNA profiles previously found in tissue samples from patients who died of meningococcal septic shock. This early detection of EV-RNA patterns, which mirrors those observed in the final stages of the disease, suggests a critical role for circulating EVs in intercellular communication during sepsis. The diversity in the underlying pathophysiology of mediators like EVs, which exhibit distinct biological patterns, may pave the way for personalized treatment strategies for sepsis patients in the future.

## Data Availability

The datasets presented in this study can be found in online repositories. Gene Expression Omnibus (GEO) repository https://www.ncbi.nlm.nih.gov/geo/ under the identifier GSE 292448 and GSE141864 in accordance with minimum information about a microarray experiment (MIAME) standard can be found in the article/**Supplementary Material**.
