# Supplementary material for: RNA profiles in extracellular vesicles from severe sepsis and meningitis patients reveal pathogen-specific immune signatures in meningococcal versus pneumococcal infections
Source: Front Cell Infect Microbiol. 2026 Apr 24;16:1730302. doi: 10.3389/fcimb.2026.1730302 (PMC13153109; doi:10.3389/fcimb.2026.1730302)
Supplement: Supplementary file 1 [file DataSheet1.zip › 170426_Supplementary files_proof.pdf]

Supplementary Material

RNA profiles in extracellular vesicles from severe sepsis and meningitis patients reveal pathogen-specific immune signatures in meningococcal versus pneumococcal infections

Berit Sletbakk Brusletto [1*], Kari Bente Foss Haug [1], Iselin Sandnes Olsen [1,2], Mari Kaarbø [3], Ole Kristoffer Olstad [1], Trude Aspelin [1], Erik Koldberg Amundsen [1,2], Petter Brandtzaeg [1, 4, 5], Reidun Øvstebø [1]

[1] Department of Medical Biochemistry, Oslo University Hospital, Oslo, Norway, [2] Department of Life Sciences and Health, Oslo Metropolitan University, Oslo, Norway, [3] Department of Microbiology, Oslo University Hospital, Oslo, Norway, [4] Department of Pediatrics, Oslo University Hospital, Oslo, Norway, [5] Institute of Clinical Medicine, Faculty of Medicine, University of Oslo, Oslo, Norway.

*Corresponding author

Email: berit.brusletto@medisin.uio.no (BSB)

Supplementary file 1 A, B, C, D, E, F Top 50 RNAs with significantly changed levels in plasma EVs from patients with meningococcal septic shock (A, B), meningococcal meningitis (C, D) and systemic pneumococcal disease (E, F) when compared against the healthy control group.

A. Top 50 RNAs with significantly higher signal levels in plasma EVs from patients with meningococcal septic shock vs. healthy ctr.

Transcript ID	Gene symbo	mrna_assignment	p-value(Nm sep vs. Ctr)	Fold-Change(Nm sep vs. Ctr)
TC0Y000084.hg.1		DQ582680 // NONCODE // accn=DQ582680 class=piRNA name=piR-32792 ref=NONCODE v2.0 transc	0,0001	60,9
TC0Y000221.hg.1		DQ576060 // NONCODE // accn=DQ576060 class=piRNA name=piR-44172 ref=NONCODE v2.0 transc	0,0001	60,9
TC15000025.hg.1		DQ599733 // NONCODE // accn=DQ599733 class=piRNA name=piR-37799 ref=NONCODE v2.0 transc	0,0005	45,2
TC15000149.hg.1		DQ597560 // NONCODE // accn=DQ597560 class=piRNA name=piR-35626 ref=NONCODE v2.0 transc	0,0005	45,2
TC15001013.hg.1		DQ600342 // NONCODE // accn=DQ600342 class=piRNA name=piR-38408 ref=NONCODE v2.0 transc	0,0005	45,2
TC15001777.hg.1		DQ574758 // NONCODE // accn=DQ574758 class=piRNA name=piR-42870 ref=NONCODE v2.0 transc	0,0010	40,3
TC0Y000098.hg.1		DQ581594 // NONCODE // accn=DQ581594 class=piRNA name=piR-49706 ref=NONCODE v2.0 transc	0,0006	37,3
TC0Y000205.hg.1		DQ574758 // NONCODE // accn=DQ574758 class=piRNA name=piR-42870 ref=NONCODE v2.0 transc	0,0006	37,3
TC15000807.hg.1		DQ574760 // NONCODE // accn=DQ574760 class=piRNA name=piR-42872 ref=NONCODE v2.0 transc	0,0006	37,3
TC12001491.hg.1		uc021qxt.1 // UCSC Genes // Nucleic acid controling mast cell degranulation. // chr12 /	0,0001	26,7
TC05001283.hg.1		ENST00000458832 // ENSEMBL // ncrna:novel chromosome:GRCh38:5:38081672:38081743:-1 gene	0,0006	26,6
TC01001058.hg.1		DQ579288 // NONCODE // accn=DQ579288 class=piRNA name=piR-47400 ref=NONCODE v2.0 transc	0,0007	26,4
TC01003066.hg.1		DQ590126 // NONCODE // accn=DQ590126 class=piRNA name=piR-57238 ref=NONCODE v2.0 transc	0,0007	26,4
TC10000174.hg.1		ENST00000408159 // ENSEMBL // ncrna:miRNA chromosome:GRCh37:10:25239333:25239409:1 gene	0,0001	25,9
TC10000239.hg.1		ENST00000517245 // ENSEMBL // RNA, U7 small nuclear 77 pseudogene [gene_biotype:snRNA t	0,0007	24,0
TC10001395.hg.1		ENST00000459103 // ENSEMBL // ncrna:novel chromosome:GRCh38:10:133120003:133120074:1 ge	0,0003	23,7
TC04000831.hg.1		uc021xuc.1 // UCSC Genes // A nucleics Acid regulating cell growth. // chr4 // 100 // 1	0,0005	23,3
TC20000132.hg.1		ENST00000401198 // ENSEMBL // ncrna:novel chromosome:GRCh38:20:19590097:19590187:-1 gen	0,0009	22,3
TC04000862.hg.1		uc021xum.1 // UCSC Genes // A nucleics Acid regulating cell growth. // chr4 // 100 // 1	0,0002	22,3
TC01000807.hg.1		uc021ooz.1 // UCSC Genes // A nucleics Acid regulating cell growth. // chr1 // 100 // 1	0,0008	21,4
TC15001479.hg.1		ENST00000410654 // ENSEMBL // ncrna:novel chromosome:GRCh38:15:56966325:56966397:-1 gen	0,0002	21,4
TC13000773.hg.1		ENST00000411127 // ENSEMBL // ncrna:novel chromosome:GRCh38:13:88187859:88187920:-1 gen	0,0005	21,2
TC0X000860.hg.1		ENST00000516302 // ENSEMBL // ncrna:novel chromosome:GRCh38:X:12148318:12148401:-1 gene	0,0008	20,9
TC14000496.hg.1		ENST00000408251 // ENSEMBL // ncrna:novel chromosome:GRCh38:14:78617866:78617975:1 gene	0,0008	20,6
TC10000506.hg.1		DQ590620 // NONCODE // accn=DQ590620 class=piRNA name=piR-57732 ref=NONCODE v2.0 transc	0,0002	20,4
TC04002546.hg.1		TCONS_l2_00021878-XLOC_l2_011118 // Broad TUCP // linc-OCIAD2-2 chr4:-:49328035-4951330	0,0023	19,8
TC15000548.hg.1		DQ580408 // NONCODE // accn=DQ580408 class=piRNA name=piR-48520 ref=NONCODE v2.0 transc	0,0002	19,8
TCUn_gl000221000001.hg.1		DQ592442 // NONCODE // accn=DQ592442 class=piRNA name=piR-59554 ref=NONCODE v2.0 transc	0,0000	19,5
TC20000132.hg.1		ENST00000401198 // ENSEMBL // ncrna:novel chromosome:GRCh38:20:19590097:19590187:-1 gen	0,0009	22,3
TC04000862.hg.1		uc021xum.1 // UCSC Genes // A nucleics Acid regulating cell growth. // chr4 // 100 // 1	0,0002	22,3
TC01000807.hg.1		uc021ooz.1 // UCSC Genes // A nucleics Acid regulating cell growth. // chr1 // 100 // 1	0,0008	21,4
TC15001479.hg.1		ENST00000410654 // ENSEMBL // ncrna:novel chromosome:GRCh38:15:56966325:56966397:-1 gen	0,0002	21,4
TC13000773.hg.1		ENST00000411127 // ENSEMBL // ncrna:novel chromosome:GRCh38:13:88187859:88187920:-1 gen	0,0005	21,2
TC0X000860.hg.1		ENST00000516302 // ENSEMBL // ncrna:novel chromosome:GRCh38:X:12148318:12148401:-1 gene	0,0008	20,9
TC14000496.hg.1		ENST00000408251 // ENSEMBL // ncrna:novel chromosome:GRCh38:14:78617866:78617975:1 gene	0,0008	20,6
TC10000506.hg.1		DQ590620 // NONCODE // accn=DQ590620 class=piRNA name=piR-57732 ref=NONCODE v2.0 transc	0,0002	20,4
TC04002546.hg.1		TCONS_l2_00021878-XLOC_l2_011118 // Broad TUCP // linc-OCIAD2-2 chr4:-:49328035-4951330	0,0023	19,8
TC15000548.hg.1		DQ580408 // NONCODE // accn=DQ580408 class=piRNA name=piR-48520 ref=NONCODE v2.0 transc	0,0002	19,8
TCUn_gl000221000001.hg.1		DQ592442 // NONCODE // accn=DQ592442 class=piRNA name=piR-59554 ref=NONCODE v2.0 transc	0,0000	19,5
TC06002101.hg.1		uc021zff.1 // UCSC Genes // Rfam model RF01061 hit found at contig region Z97352.1/1623	0,0009	19,3
TC10002741.hg.1		OTTHUMT00000049107 // NONCODE // putative novel transcript[gene_biotype:lincRNA transcr	0,0001	19,3
TC07000434.hg.1		DQ596866 // NONCODE // accn=DQ596866 class=piRNA name=piR-34932 ref=NONCODE v2.0 transc	0,0001	19,0
TC16001204.hg.1		ENST00000408862 // ENSEMBL // ncrna:novel chromosome:GRCh38:16:68055779:68055884:-1 gen	0,0010	18,9
TC22000897.hg.1		ENST00000516103 // ENSEMBL // ncrna:novel chromosome:GRCh38:22:48814320:48814404:-1 gen	0,0003	18,6
TC16000276.hg.1		DQ583840 // NONCODE // accn=DQ583840 class=piRNA name=piR-50952 ref=NONCODE v2.0 transc	0,0013	18,4
TC06000129.hg.1		ENST00000517140 // ENSEMBL // ncrna:novel chromosome:GRCh38:6:20422318:20422401:1 gene:	0,0003	18,2
TC10000394.hg.1		ENST00000517203 // ENSEMBL // ncrna:novel chromosome:GRCh38:10:65710060:65710144:1 gene	0,0007	18,0
TC16000152.hg.1	MIR548X	NR_036166 // RefSeq // Homo sapiens microRNA 548x (MIR548X), microRNA. // chr16 // 100	0,0053	17,9
TC07000265.hg.1		ENST00000516499 // ENSEMBL // ncrna:novel chromosome:GRCh38:7:43578888:43578972:1 gene:	0,0003	17,9
TC05000222.hg.1		ENST00000458939 // ENSEMBL // ncrna:novel chromosome:GRCh38:5:53609587:53609658:1 gene:	0,0020	17,8

B. Top 50 RNAs with significantly lower signal levels in plasma EVs from patients with meningococcal septic shock vs. healthy ctr.

Transcript ID	Gene symbol	mrna_assignment	p-value(Nm sep vs. Ct	Fold-Change(Nm s
TC05000673.hg.1	MIR4461	NR_039666 // RefSeq // Homo sapiens microRNA 4461 (MIR4461), microRNA. // chr5 // 100 /	3,1E-05	-34,3
TC0M000006.hg.1		AB017116 // GenBank // Homo sapiens mitochondrial mRNA for AD 1, partial cds. // chrM /	1,2E-04	-32,7
TC01004109.hg.1		CR623081 // NONCODE // accn=CR623081 class=mRNAlike lncRNA name=Human lncRNA ref=Jounra	1,6E-04	-30,4
TC0M000025.hg.1		M28016 // NONCODE // accn=M28016 class=mRNAlike lncRNA name=NULL ref=H-invitational v7.	1,8E-04	-25,4
TC0M000005.hg.1		uc011mfi.1 // NONCODE // accn=NULL class=lncRNA name= ref=UCSCGeneNoncode transcriptId=	6,7E-04	-23,4
TC0M000009.hg.1		uc004cox.3 // NONCODE // accn=NULL class=lncRNA name= ref=UCSCGeneNoncode transcriptId=	3,0E-04	-21,8
TC0M000007.hg.1		BC061915 // GenBank HTC // Homo sapiens cDNA clone IMAGE:3887455, **** WARNING: chimeri	5,8E-04	-20,0
TC01000013.hg.1		M37726 // GenBank // Human mitochondrial Lys-tRNA-aaa. // chr1 // 100 // 100 // 0 // --	1,4E-04	-19,3
TC0M000023.hg.1		uc004cov.3 // NONCODE // accn=NULL class=lncRNA name= ref=UCSCGeneNoncode transcriptId=	3,0E-04	-19,0
TC07000959.hg.1		DQ597485 // NONCODE // accn=DQ597485 class=piRNA name=piR-35551 ref=NONCODE v2.0 transc	3,4E-05	-18,9
TC0M000003.hg.1		S81656 // GenBank // tRNA(Ile) [human, skeletal muscle_progressive external ophtalmople	8,5E-04	-18,3
TC0M000021.hg.1		AB019563 // NONCODE // accn=AB019563 class=mRNAlike lncRNA name=NULL ref=H-invitational	2,4E-05	-17,9
TC0M000010.hg.1		uc022bqw.1 // UCSC Genes // Homo sapiens clone 35w unknown mRNA; mitochondrial. // chrM	7,4E-05	-17,8
TC0M000024.hg.1		uc004cox.3 // NONCODE // accn=NULL class=lncRNA name= ref=UCSCGeneNoncode transcriptId=	3,5E-05	-17,3
TC0M000016.hg.1		uc022bqs.1 // UCSC Genes // Homo sapiens piRNA piR-34804, complete sequence. // chrM //	2,1E-05	-15,4
TC01004110.hg.1		CR598056 // NONCODE // accn=CR598056 class=mRNAlike lncRNA name=Human lncRNA ref=Jounra	8,9E-05	-15,2
TC05000672.hg.1		ENST00000458878 // ENSEMBL // ncrna:miRNA chromosome:GRCh37:5:134259743:134259812:1 gen	4,6E-04	-14,6
TC0M000004.hg.1		BC018860 // NONCODE // accn=BC018860 class=Non-protein coding transcript name=NULL ref=	2,9E-04	-14,1
TC0M000027.hg.1		BC017047 // NONCODE // accn=BC017047 class=mRNAlike lncRNA name=NULL ref=H-invitational	2,0E-03	-13,7
TC0M000022.hg.1		BC013932 // NONCODE // accn=BC013932 class=mRNAlike lncRNA name=NULL ref=H-invitational	5,0E-04	-13,4
TC01002064.hg.1		ENST00000459059 // ENSEMBL // ncrna:miRNA chromosome:GRCh37:1:566187:566265:-1 gene:ENS	4,1E-04	-12,4
TC0M000019.hg.1		uc022bqx.1 // UCSC Genes // Sequence 933 from Patent WO2010139812. // chrM // 100 // 10	3,1E-03	-11,9
TC03002425.hg.1		TCONS_00006915 // NONCODE // accn=NULL class=lncRNA name=Human lincRNA ref=BodyMapLinc	7,8E-04	-11,8
TC0M000002.hg.1		DQ582265 // NONCODE // accn=DQ582265 class=piRNA name=piR-32377 ref=NONCODE v2.0 transc	2,8E-03	-10,8
TC02001953.hg.1		ENST00000502349 // ENSEMBL // [retired] ncrna:lincRNA chromosome:GRCh37:2:70016684:7002	4,2E-05	-10,7
TC04001282.hg.1	PPBP	NM_002704 // RefSeq // Homo sapiens pro-platelet basic protein (chemokine (C-X-C motif)	1,8E-03	-10,6
TC01005191.hg.1		linc_luo_1279 // NONCODE // accn=NULL class=lincRNA name=Human lincRNA ref=Scripture Re	3,4E-06	-10,3
TC06000695.hg.1		ENST00000544932 // ENSEMBL // ensembl:known chromosome:GRCh38:6:61574103:61574629:1 gen	6,7E-04	-10,0
TC01004111.hg.1		CR614989 // NONCODE // accn=CR614989 class=mRNAlike lncRNA name=Human lncRNA ref=Jounra	2,0E-06	-9,7
TC0M000026.hg.1		uc004coz.1 // NONCODE // accn=NULL class=lncRNA name= ref=UCSCGeneNoncode transcriptId=	1,4E-05	-9,7
TC02000850.hg.1		uc021vpo.1 // UCSC Genes // transfer RNA pseudogene (anticodon TTG) // chr2 // 100 // 1	2,3E-03	-9,0
TC04001281.hg.1	PF4	NM_002619 // RefSeq // Homo sapiens platelet factor 4 (PF4), mRNA. // chr4 // 100 // 10	1,7E-03	-8,7
TC01004112.hg.1		uc001abc.2 // NONCODE // accn=NULL class=lncRNA name= ref=UCSCGeneNoncode transcriptId=	3,4E-05	-8,7
TC11001411.hg.1	MTRNR2L8	NM_001190702 // RefSeq // Homo sapiens MT-RNR2-like 8 (MTRNR2L8), mRNA. // chr11 // 100	6,0E-05	-8,7
TC0M000017.hg.1		uc022bqt.1 // UCSC Genes // Homo sapiens piRNA piR-31490, complete sequence. // chrM //	2,5E-07	-8,2
TC17001269.hg.1		uc021tsy.1 // UCSC Genes // Rfam model RF00005 hit found at contig region AC131055.10/1	3,4E-04	-8,0
TC20000468.hg.1	TUBB1	NM_030773 // RefSeq // Homo sapiens tubulin, beta 1 class VI (TUBB1), mRNA. // chr20 //	1,0E-05	-8,0
TC0M000011.hg.1		AF079515 // GenBank // Homo sapiens IDL RNA, complete sequence; mitochondrial. // chrM	9,5E-05	-7,8
TC05001540.hg.1	MTRNR2L2	NM_001190470 // RefSeq // Homo sapiens MT-RNR2-like 2 (MTRNR2L2), mRNA. // chr5 // 100	2,8E-05	-7,6
TC10002540.hg.1		TCONS_00018467 // NONCODE // accn=NULL class=lncRNA name=Human lincRNA ref=BodyMapLinc	7,7E-04	-7,6
TC01001619.hg.1	RGS18	NM_130782 // RefSeq // Homo sapiens regulator of G-protein signaling 18 (RGS18), mRNA.	1,6E-04	-7,6
TC01004011.hg.1		uc021plb.1 // UCSC Genes // transfer RNA pseudogene (anticodon TAA) // chr1 // 100 // 1	1,2E-04	-7,4
TC05003245.hg.1		CR623832 // NONCODE // accn=CR623832 class=mRNAlike lncRNA name=NULL ref=H-invitational	1,5E-05	-7,3
TC0M000012.hg.1		DQ582201 // NONCODE // accn=DQ582201 class=piRNA name=piR-32313 ref=NONCODE v2.0 transc	8,0E-03	-6,4
TC06000758.hg.1	SH3BGR	NM_031469 // RefSeq // Homo sapiens SH3 domain binding glutamate-rich protein like 2 (S	6,5E-06	-6,1
TC07000373.hg.1		uc022aex.1 // UCSC Genes // Rfam model RF00005 hit found at contig region AC115220.1/10	1,3E-03	-6,0
TC11003052.hg.1		CR599987 // NONCODE // accn=CR599987 class=mRNAlike lncRNA name=Human lncRNA ref=Jounra	4,6E-07	-6,0
TC11001143.hg.1		NM_001126181 // RefSeq // Homo sapiens neurogranin (protein kinase C substrate, RC3) (N	3,1E-04	-5,9
TC15002179.hg.1		X07621 // NONCODE // accn=X07621 class=mRNAlike lncRNA name=NULL ref=H-invitational v7.	4,0E-04	-5,8
TC17001268.hg.1		uc021tsx.1 // UCSC Genes // Rfam model RF00005 hit found at contig region AC131055.10/1	1,9E-04	-5,6

C. Top 50 RNAs with significantly higher signal levels in plasma EVs from patients with meningococcal meningitis vs. healthy ctr.

Transcript ID	Gene Symbol	mrna_assignment	p-value(Nm men vs. Ctr)	Fold-Change(Nm men vs. Ctr)
TC02003832.hg.1		AB032733 // NONCODE // accn=AB032733 class=mRNAlike lncRNA name=NULL ref=H-invitational	0,014	7,0
TC01003260.hg.1	S100A12	NM_005621 // RefSeq // Homo sapiens S100 calcium binding protein A12 (S100A12), mRNA. /	0,041	5,4
TC07000434.hg.1		DQ596866 // NONCODE // accn=DQ596866 class=piRNA name=piR-34932 ref=NONCODE v2.0 transc	0,038	4,8
TC07001523.hg.1		DQ596866 // NONCODE // accn=DQ596866 class=piRNA name=piR-34932 ref=NONCODE v2.0 transc	0,038	4,8
TC01001254.hg.1	S100A9	NM_002965 // RefSeq // Homo sapiens S100 calcium binding protein A9 (S100A9), mRNA. //	0,016	4,2
TC11002483.hg.1	IFITM2	CR541874 // NONCODE // accn=CR541874 class=mRNAlike lncRNA name=NULL ref=H-invitational	0,030	3,9
TC15000441.hg.1	AQP9	NM_020980 // RefSeq // Homo sapiens aquaporin 9 (AQP9), mRNA. // chr15 // 100 // 100 //	0,001	3,9
TC07002222.hg.1	CREB5	AK057151 // NONCODE // accn=AK057151 class=mRNAlike lncRNA name=Human lncRNA ref=Jounra	0,029	3,8
TC01001346.hg.1	MNDA	NM_002432 // RefSeq // Homo sapiens myeloid cell nuclear differentiation antigen (MNDA)	0,028	3,6
TC21001069.hg.1	SAMSN1	NM_022136 // RefSeq // Homo sapiens SAM domain, SH3 domain and nuclear localization sig	0,015	3,5
TC01001624.hg.1	RGS2	NM_002923 // RefSeq // Homo sapiens regulator of G-protein signaling 2 (RGS2), mRNA. //	0,024	3,0
TC14001581.hg.1	ECRP	NR_033909 // NONCODE // accn=NR_033909 class=lncRNA name= ref=RefGeneNoncode transcript	0,033	2,8
TC06003855.hg.1	VNN2	D89974 // NONCODE // accn=D89974 class=mRNAlike lncRNA name=Human lncRNA ref=JounralRNA	0,023	2,7
TC11000010.hg.1	IFITM2	NM_006435 // RefSeq // Homo sapiens interferon induced transmembrane protein 2 (IFITM2)	0,039	2,7
TC14002137.hg.1	DICER1	AY845867 // NONCODE // accn=AY845867 class=mRNAlike lncRNA name=NULL ref=H-invitational	0,008	2,7
TC10001182.hg.1		ENST00000543053 // ENSEMBL // ensembl:known chromosome:GRCh38:10:36522819:36524237:-1 g	0,012	2,5
TC06000294.hg.1		uc021ysn.1 // UCSC Genes // transfer RNA Ala (anticodon CGC) // chr6 // 100 // 100 // 0	0,008	2,5
TC01003261.hg.1	S100A8	NM_002964 // RefSeq // Homo sapiens S100 calcium binding protein A8 (S100A8), mRNA. //	0,029	2,5
TC05002420.hg.1		TCONS_l2_00023407-XLOC_l2_011619 // Broad TUCP // linc-SERF1A-3 chr5:+:68902925-6891987	0,007	2,4
TC03001888.hg.1	TM4SF1	NM_014220 // RefSeq // Homo sapiens transmembrane 4 L six family member 1 (TM4SF1), mRN	0,023	2,4
TC10002542.hg.1	ZNF438	NR_026560 // NONCODE // accn=NR_026560 class=lncRNA name= ref=RefGeneNoncode transcript	0,025	2,4
TC02004970.hg.1	MXD1	NM_001202513 // RefSeq // Homo sapiens MAX dimerization protein 1 (MXD1), transcript va	0,027	2,4
TC06001488.hg.1	IFITM4P	NR_001590 // RefSeq // Homo sapiens interferon induced transmembrane protein 4 pseudoge	0,017	2,3
TC6_mcf_hap500	IFITM4P	NR_001590 // RefSeq // Homo sapiens interferon induced transmembrane protein 4 pseudoge	0,017	2,3
TC02003238.hg.1	SPTBN1	BC032061 // NONCODE // accn=BC032061 class=mRNAlike lncRNA name=Human lncRNA ref=Jounra	0,036	2,3
TC03001550.hg.1	PROK2	NM_001126128 // RefSeq // Homo sapiens prokineticin 2 (PROK2), transcript variant 1, mR	0,019	2,3
TC14002287.hg.1	ECRP	NR_033909 // RefSeq // Homo sapiens ribonuclease, RNase A family, 2 (liver, eosinophil-	0,016	2,2
TC06001470.hg.1		uc021ytd.1 // UCSC Genes // transfer RNA Ala (anticodon AGC) // chr6 // 100 // 100 // 0	0,012	2,2
TC06001472.hg.1		uc021yte.1 // UCSC Genes // transfer RNA Ala (anticodon AGC) // chr6 // 100 // 100 // 0	0,012	2,2
TC11000504.hg.1	MS4A3	NM_001031666 // RefSeq // Homo sapiens membrane-spanning 4-domains, subfamily A, member	0,036	2,2
TC06003570.hg.1		TCONS_l2_00025363-XLOC_l2_012943 // Broad TUCP // linc-ZFP57-2 chr6:-:29718584-29718925	0,013	2,1
TC19001787.hg.1	FPR1	NM_001193306 // RefSeq // Homo sapiens formyl peptide receptor 1 (FPR1), transcript var	0,037	2,1
TC01004862.hg.1		TCONS_00001210 // NONCODE // accn=NULL class=lncRNA name=Human lincRNA ref=BodyMapLinc	0,011	2,1
TC06000292.hg.1		uc021ysk.1 // UCSC Genes // transfer RNA Ala (anticodon AGC) // chr6 // 100 // 100 // 0	0,017	2,1
TC01003616.hg.1	NCF2	NM_000433 // RefSeq // Homo sapiens neutrophil cytosolic factor 2 (NCF2), transcript va	0,022	2,1
TC01002287.hg.1	PADI2	NM_007365 // RefSeq // Homo sapiens peptidyl arginine deiminase, type II (PADI2), mRNA.	0,033	2,0
TC6_mann_hap4000180.hg.1		TCONS_00030002 // NONCODE // accn=NULL class=lncRNA name=Human lincRNA ref=BodyMapLinc	0,046	2,0
TC6_qbl_hap6000205.hg.1		TCONS_00029991-XLOC_014476 // Rinn lincRNA // linc-ZIC1-6 chr6_qbl_hap6:-:1019444-10197	0,046	2,0
TC6_cox_hap200(	IFITM4P	NR_001590 // RefSeq // Homo sapiens interferon induced transmembrane protein 4 pseudoge	0,025	2,0
TC12001178.hg.1	CLEC4E	NM_014358 // RefSeq // Homo sapiens C-type lectin domain family 4, member E (CLEC4E), m	0,041	2,0
TC6_dbb_hap3000205.hg.1		TCONS_00029967 // NONCODE // accn=NULL class=lncRNA name=Human lincRNA ref=BodyMapLinc	0,034	2,0
TC19002498.hg.1		TCONS_l2_00013127-XLOC_l2_007062 // Broad TUCP // linc-PSG9-2 chr19:-:43877099-43877707	0,042	2,0
TC6_dbb_hap300	IFITM4P	NR_001590 // RefSeq // Homo sapiens interferon induced transmembrane protein 4 pseudoge	0,025	2,0
TC6_mann_hap4(	IFITM4P	NR_001590 // RefSeq // Homo sapiens interferon induced transmembrane protein 4 pseudoge	0,025	2,0
TC6_qbl_hap600(	IFITM4P	NR_001590 // RefSeq // Homo sapiens interferon induced transmembrane protein 4 pseudoge	0,025	2,0
TC14001030.hg.1	BAZ1A	NM_013448 // RefSeq // Homo sapiens bromodomain adjacent to zinc finger domain, 1A (BAZ	0,047	2,0
TC12003096.hg.1	TBX3	AY034105 // NONCODE // accn=AY034105 class=mRNAlike lncRNA name=NULL ref=H-invitational	0,033	2,0
TC01005397.hg.1		CR597056 // NONCODE // accn=CR597056 class=mRNAlike lncRNA name=NULL ref=H-invitational	0,031	2,0
TC6_mcf_hap500	FLOT1	NM_005803 // RefSeq // Homo sapiens flotillin 1 (FLOT1), mRNA. // chr6_mcf_hap5 // 100	0,018	1,9
TC01003283.hg.1	RAB13	NM_002870 // RefSeq // Homo sapiens RAB13, member RAS oncogene family (RAB13), transcri	0,038	1,9

D. Top 50 RNAs with significantly lower signal levels in plasma EVs from patients with meningococcal meningitis vs. healthy ctr.

Transcript ID	Gene Symbol	mrna_assignment	p-value(Nm men vs. Ctr)	Fold-Change(Nm men vs. Ctr)
TC06001999.hg.1		ENST00000459439 // ENSEMBL // ncrna:snoRNA chromosome:GRCh37:6:109612458:109612561:-1 g	0,027	-2,7
TC06002911.hg.1		linc_luo_466 // NONCODE // accn=NULL class=lincRNA name=Human lincRNA ref=Scripture Rec	0,021	-2,6
TC17001566.hg.1		DQ592711 // NONCODE // accn=DQ592711 class=piRNA name=piR-59823 ref=NONCODE v2.0 transc	0,029	-2,4
TC0X002231.hg.1	MBNL3	CR627122 // NONCODE // accn=CR627122 class=mRNAlike lncRNA name=Human lncRNA ref=Jounra	0,048	-2,2
TC01001475.hg.1	BLZF1	NM_003666 // RefSeq // Homo sapiens basic leucine zipper nuclear factor 1 (BLZF1), mRNA	0,027	-2,1
TC0X001543.hg.1	GAB3	NM_001081573 // RefSeq // Homo sapiens GRB2-associated binding protein 3 (GAB3), transc	0,011	-2,1
TC19002293.hg.1	AES	X73357 // NONCODE // accn=X73357 class=mRNAlike lncRNA name=NULL ref=H-invitational v7.	0,048	-2,0
TC0X000599.hg.1		ENST00000408290 // ENSEMBL // ncrna:novel chromosome:GRCh38:X:121868867:121868956:1 gen	0,046	-2,0
TC16001776.hg.1	GSPT1	BC008391 // NONCODE // accn=BC008391 class=mRNAlike lncRNA name=NULL ref=H-invitational	0,006	-1,9
TC0X001370.hg.1	MBNL3	NM_001170701 // RefSeq // Homo sapiens muscleblind-like splicing regulator 3 (MBNL3), t	0,034	-1,9
TC06000497.hg.1		DQ579683 // NONCODE // accn=DQ579683 class=piRNA name=piR-47795 ref=NONCODE v2.0 transc	0,016	-1,8
TC03001317.hg.1		ENST00000390843 // ENSEMBL // U8 small nucleolar RNA [gene_biotype:snoRNA transcript_bi	0,048	-1,8
TC04002908.hg.1		TCONS_00008726 // NONCODE // accn=NULL class=lncRNA name=Human lincRNA ref=BodyMapLinc	0,002	-1,8
TC15000538.hg.1		DQ578824 // NONCODE // accn=DQ578824 class=piRNA name=piR-46936 ref=NONCODE v2.0 transc	0,029	-1,8
TC17002037.hg.1	MPDU1	NR_024603 // NONCODE // accn=NR_024603 class=lncRNA name= ref=RefGeneNoncode transcript	0,031	-1,8
TC15002708.hg.1		BC043379 // NONCODE // accn=BC043379 class=mRNAlike lncRNA name=Human lncRNA ref=Jounra	0,036	-1,8
TC03002164.hg.1	LINC01063	ENST00000441644 // ENSEMBL // long intergenic non-protein coding RNA 1063 [gene_biotype	0,002	-1,7
TC01005480.hg.1		OTTHUMT00000022441 // NONCODE // putative novel transcript (FLJ46006)[gene_biotype:linc	0,043	-1,7
TC19001041.hg.1	AES	NM_001130 // RefSeq // Homo sapiens amino-terminal enhancer of split (AES), transcript	0,024	-1,7
TC19001981.hg.1	PRKCSH	Z36798 // NONCODE // accn=Z36798 class=mRNAlike lncRNA name=NULL ref=H-invitational v7.	0,007	-1,7
TC19002285.hg.1	TCF3	M24404 // NONCODE // accn=M24404 class=mRNAlike lncRNA name=NULL ref=H-invitational v7.	0,036	-1,7
TC14002084.hg.1		AK124866 // NONCODE // accn=AK124866 class=mRNAlike lncRNA name=Human lncRNA ref=Jounra	0,028	-1,7
TC19001322.hg.1		DQ583759 // NONCODE // accn=DQ583759 class=piRNA name=piR-50871 ref=NONCODE v2.0 transc	0,048	-1,7
TC07001526.hg.1		ENST00000365308 // ENSEMBL // ncrna:misc_RNA chromosome:GRCh37:7:74955146:74955247:-1 g	0,001	-1,7
TC07000493.hg.1		ENST00000365015 // ENSEMBL // Y RNA [gene_biotype:misc_RNA transcript_biotype:misc_RNA]	0,001	-1,7
TC07001524.hg.1		ENST00000362771 // ENSEMBL // ncrna:misc_RNA chromosome:GRCh37:7:74927121:74927222:-1 g	0,001	-1,7
TC07000461.hg.1		ENST00000459002 // ENSEMBL // Y RNA [gene_biotype:misc_RNA transcript_biotype:misc_RNA]	0,001	-1,7
TC07000494.hg.1		ENST00000365089 // ENSEMBL // Y RNA [gene_biotype:misc_RNA transcript_biotype:misc_RNA]	0,001	-1,7
TC07001528.hg.1		ENST00000363000 // ENSEMBL // Y RNA [gene_biotype:misc_RNA transcript_biotype:misc_RNA]	0,001	-1,7
TC07000433.hg.1		ENST00000364412 // ENSEMBL // Y RNA [gene_biotype:misc_RNA transcript_biotype:misc_RNA]	0,001	-1,7
TC07000436.hg.1		ENST00000363043 // ENSEMBL // Y RNA [gene_biotype:misc_RNA transcript_biotype:misc_RNA]	0,001	-1,7
TC07001519.hg.1		ENST00000365151 // ENSEMBL // Y RNA [gene_biotype:misc_RNA transcript_biotype:misc_RNA]	0,001	-1,7
TC11002716.hg.1		TCONS_00019144 // NONCODE // accn=NULL class=lncRNA name=Human lincRNA ref=BodyMapLinc	0,024	-1,7
TC07001667.hg.1		ENST00000365043 // ENSEMBL // Y RNA [gene_biotype:misc_RNA transcript_biotype:misc_RNA]	0,003	-1,6
TC02004602.hg.1	CCNT2-AS1	NR_036549 // NONCODE // accn=NR_036549 class=lncRNA name= ref=RefGeneNoncode transcript	0,004	-1,6
TC09001652.hg.1		ENST00000440413 // ENSEMBL // PRRX2 antisense RNA 1 [gene_biotype:antisense transcript_	0,036	-1,6
TC16000566.hg.1		ENST00000363294 // ENSEMBL // Y RNA [gene_biotype:misc_RNA transcript_biotype:misc_RNA]	0,011	-1,6
TC0X001027.hg.1	GPKOW	NM_015698 // RefSeq // Homo sapiens G patch domain and KOW motifs (GPKOW), mRNA. // chr	0,026	-1,6
TC05002085.hg.1		OTTHUMT00000371946 // Havana transcript // TEC[gene_biotype:TEC transcript_biotype:TEC]	0,030	-1,6
TC17_ctg5_hap100	KANSL1-AS1	NR_034172 // RefSeq // Homo sapiens KANSL1 antisense RNA 1 (KANSL1-AS1), long non-codin	0,035	-1,6
TC17_ctg5_hap100	KANSL1-AS1	FJ009034 // GenBank // Homo sapiens non-coding RNA 230388, complete sequence. // chr17_	0,007	-1,6
TC11001060.hg.1	CD3E	NM_000733 // RefSeq // Homo sapiens CD3e molecule, epsilon (CD3-TCR complex) (CD3E), mR	0,035	-1,6
TC0X001875.hg.1		U31735 // NONCODE // accn=U31735 class=mRNAlike lncRNA name=NULL ref=H-invitational v7.	0,042	-1,6
TC0Y000156.hg.1		ENST00000516480 // ENSEMBL // RNA, 5S ribosomal pseudogene 519 [gene_biotype:rRNA trans	0,045	-1,6
TC07002634.hg.1	IL23A	M97713 // NONCODE // accn=M97713 class=mRNAlike lncRNA name=NULL ref=H-invitational v7.	0,002	-1,6
TC05001806.hg.1		ENST00000458850 // ENSEMBL // ncrna:novel chromosome:GRCh38:5:135713119:135713216:-1 ge	0,025	-1,6
TC17000142.hg.1		ENST00000516554 // ENSEMBL // RNA, U7 small nuclear 43 pseudogene [gene_biotype:snRNA t	0,011	-1,6
TC01001062.hg.1		DQ592442 // NONCODE // accn=DQ592442 class=piRNA name=piR-59554 ref=NONCODE v2.0 transc	0,017	-1,6
TC02001209.hg.1		ENST00000384656 // ENSEMBL // Y RNA [gene_biotype:misc_RNA transcript_biotype:misc_RNA]	0,031	-1,5
TC19002578.hg.1		TCONS_00026857 // NONCODE // accn=NULL class=lncRNA name=Human lincRNA ref=BodyMapLinc	0,007	-1,5

E. Top 50 RNAs with significantly higher signal levels in plasma EVs from patients with systemic pneumococcal disease vs. healthy ctr.

Transcript ID	Gene Symbol	mrna_assignment	p-value(Pneum vs. Ctr)	Fold-Change(Pneum vs. Ctr)
TC15001779.hg.1		DQ576060 // NONCODE // accn=DQ576060 class=piRNA name=piR-44172 ref=NONCODE v2.0 transc	0,015	34,6
TC15000788.hg.1		DQ582680 // NONCODE // accn=DQ582680 class=piRNA name=piR-32792 ref=NONCODE v2.0 transc	0,015	34,6
TC15001114.hg.1		DQ600342 // NONCODE // accn=DQ600342 class=piRNA name=piR-38408 ref=NONCODE v2.0 transc	0,045	21,4
TC15000025.hg.1		DQ599733 // NONCODE // accn=DQ599733 class=piRNA name=piR-37799 ref=NONCODE v2.0 transc	0,045	21,4
TC15001156.hg.1		DQ600342 // NONCODE // accn=DQ600342 class=piRNA name=piR-38408 ref=NONCODE v2.0 transc	0,048	19,2
TC15001753.hg.1		DQ574758 // NONCODE // accn=DQ574758 class=piRNA name=piR-42870 ref=NONCODE v2.0 transc	0,008	18,4
TC15000826.hg.1		DQ574760 // NONCODE // accn=DQ574760 class=piRNA name=piR-42872 ref=NONCODE v2.0 transc	0,008	18,4
TC15000811.hg.1		DQ581594 // NONCODE // accn=DQ581594 class=piRNA name=piR-49706 ref=NONCODE v2.0 transc	0,008	18,4
TC15001777.hg.1		DQ574758 // NONCODE // accn=DQ574758 class=piRNA name=piR-42870 ref=NONCODE v2.0 transc	0,012	17,7
TC0Y000220.hg.1		DQ574758 // NONCODE // accn=DQ574758 class=piRNA name=piR-42870 ref=NONCODE v2.0 transc	0,028	14,2
TC0Y000085.hg.1		DQ581594 // NONCODE // accn=DQ581594 class=piRNA name=piR-49706 ref=NONCODE v2.0 transc	0,028	14,2
TC0X001200.hg.1		DQ580189 // NONCODE // accn=DQ580189 class=piRNA name=piR-48301 ref=NONCODE v2.0 transc	0,010	12,6
TC15002039.hg.1		DQ575740 // NONCODE // accn=DQ575740 class=piRNA name=piR-43852 ref=NONCODE v2.0 transc	0,003	12,2
TC04002546.hg.1		TCONS_l2_00021878-XLOC_l2_011118 // Broad TUCP // linc-OCIAD2-2 chr4:-:49328035-4951330	0,040	11,6
TC05001283.hg.1		ENST00000458832 // ENSEMBL // ncrna:novel chromosome:GRCh38:5:38081672:38081743:-1 gene	0,046	11,5
TC12001491.hg.1		uc021qxt.1 // UCSC Genes // Nucleic acid controling mast cell degranulation. // chr12 /	0,047	11,2
TC17001400.hg.1		DQ571391 // NONCODE // accn=DQ571391 class=piRNA name=piR-31503 ref=NONCODE v2.0 transc	0,009	10,5
TC17000212.hg.1		DQ585853 // NONCODE // accn=DQ585853 class=piRNA name=piR-52965 ref=NONCODE v2.0 transc	0,009	10,5
TC01000807.hg.1		uc021ooz.1 // UCSC Genes // A nucleics Acid regulating cell growth. // chr1 // 100 // 1	0,026	10,3
TC10000506.hg.1		DQ590620 // NONCODE // accn=DQ590620 class=piRNA name=piR-57732 ref=NONCODE v2.0 transc	0,044	9,5
TC01002740.hg.1		ENST00000458856 // ENSEMBL // RNA, U7 small nuclear 62 pseudogene [gene_biotype:snRNA t	0,016	9,4
TC20000392.hg.1		ENST00000516314 // ENSEMBL // ncrna:snRNA chromosome:GRCh37:20:48444529:48444590:1 gene	0,048	8,8
TC06000716.hg.1		ENST00000458875 // ENSEMBL // RNA, U7 small nuclear 48 pseudogene [gene_biotype:snRNA t	0,034	8,7
TC06003702.hg.1		TCONS_00012197 // NONCODE // accn=NULL class=lncRNA name=Human lincRNA ref=BodyMapLinc	0,046	8,7
TC10000286.hg.1		ENST00000459472 // ENSEMBL // ncrna:novel chromosome:GRCh38:10:44034540:44034611:1 gene	0,026	8,4
TC06002038.hg.1		ENST00000408672 // ENSEMBL // ncrna:novel chromosome:GRCh38:6:114277385:114277478:-1 ge	0,043	8,3
TC16000970.hg.1		ENST00000458867 // ENSEMBL // RNA, U7 small nuclear 24 pseudogene [gene_biotype:snRNA t	0,042	8,0
TC02000832.hg.1		DQ600793 // NONCODE // accn=DQ600793 class=piRNA name=piR-38859 ref=NONCODE v2.0 transc	0,021	7,9
TC02002338.hg.1		DQ589348 // NONCODE // accn=DQ589348 class=piRNA name=piR-56460 ref=NONCODE v2.0 transc	0,021	7,9
TC06000568.hg.1		ENST00000458852 // ENSEMBL // ncrna:miRNA chromosome:GRCh37:6:41323187:41323246:1 gene:	0,004	7,9
TC15001780.hg.1		DQ601279 // NONCODE // accn=DQ601279 class=piRNA name=piR-39345 ref=NONCODE v2.0 transc	0,014	7,8
TC13000746.hg.1		DQ588544 // NONCODE // accn=DQ588544 class=piRNA name=piR-55656 ref=NONCODE v2.0 transc	0,007	7,6
TC06001431.hg.1		ENST00000458980 // ENSEMBL // RNA, U7 small nuclear 26 pseudogene [gene_biotype:snRNA t	0,035	7,5
TC01002356.hg.1		DQ588542 // NONCODE // accn=DQ588542 class=piRNA name=piR-55654 ref=NONCODE v2.0 transc	0,017	7,5
TC04000285.hg.1		ENST00000408488 // ENSEMBL // ncrna:novel chromosome:GRCh38:4:49198207:49198289:1 gene:	0,036	7,5
TC21000269.hg.1		ENST00000458847 // ENSEMBL // ncrna:novel chromosome:GRCh38:21:9098863:9098944:-1 gene:	0,039	7,3
TC01001275.hg.1		ENST00000459540 // ENSEMBL // RNA, U7 small nuclear 57 pseudogene [gene_biotype:snRNA t	0,037	7,3
TC15002025.hg.1		DQ582666 // NONCODE // accn=DQ582666 class=piRNA name=piR-32778 ref=NONCODE v2.0 transc	0,007	7,3
TC05001224.hg.1		uc021xxa.1 // UCSC Genes // Rfam model RF00998 hit found at contig region AC008768.8/25	0,031	7,3
TC03001231.hg.1		ENST00000408732 // ENSEMBL // ncrna:novel chromosome:GRCh38:3:21138263:21138341:-1 gene	0,048	7,2
TC02001877.hg.1		ENST00000515929 // ENSEMBL // ncrna:novel chromosome:GRCh38:2:58062581:58062665:-1 gene	0,030	7,1
TC10000916.hg.1		ENST00000401153 // ENSEMBL // ncrna:novel chromosome:GRCh38:10:125842944:125843029:1 ge	0,038	7,1
TC0X000630.hg.1		uc022cea.1 // UCSC Genes // Rfam model RF01061 hit found at contig region Z86064.2/6306	0,023	6,9
TC07002627.hg.1		X64982 // NONCODE // accn=X64982 class=mRNAlike lncRNA name=Human lncRNA ref=JounralRNA	0,038	6,9
TC09001627.hg.1		DQ575742 // NONCODE // accn=DQ575742 class=piRNA name=piR-43854 ref=NONCODE v2.0 transc	0,045	6,9
TC15000958.hg.1		DQ575742 // NONCODE // accn=DQ575742 class=piRNA name=piR-43854 ref=NONCODE v2.0 transc	0,008	6,8
TC15002021.hg.1		DQ593630 // NONCODE // accn=DQ593630 class=piRNA name=piR-33742 ref=NONCODE v2.0 transc	0,008	6,8
TCUn_gl000228000014.hg.1		HQ266764 // GenBank // Homo sapiens clone 225-1 DUX4 mRNA, 3 UTR, exons 2, 6, and 7, al	0,014	6,8
TC15001522.hg.1		DQ573033 // NONCODE // accn=DQ573033 class=piRNA name=piR-41145 ref=NONCODE v2.0 transc	0,038	6,8
TC15000159.hg.1		DQ575741 // NONCODE // accn=DQ575741 class=piRNA name=piR-43853 ref=NONCODE v2.0 transc	0,042	6,8

F. Top 50 RNAs with significantly lower signal levels in plasma EVs from patients with systemic pneumococcal disease vs. healthy ctr.

Transcript ID	Gene Symbol	mrna_assignment	p-value(Pneum vs. Ctr)	Fold-Change(Pneum vs. Ctr
TC01004109.hg.1		CR623081 // NONCODE // accn=CR623081 class=mRNAlike lncRNA name=Human lncRNA ref=Jounra	5,8E-05	-66,1
TC05000673.hg.1	MIR4461	NR_039666 // RefSeq // Homo sapiens microRNA 4461 (MIR4461), microRNA. // chr5 // 100 /	2,6E-08	-56,0
TC0M000006.hg.1		AB017116 // GenBank // Homo sapiens mitochondrial mRNA for AD 1, partial cds. // chrM /	2,6E-04	-47,0
TC0M000005.hg.1		uc011mfi.1 // NONCODE // accn=NULL class=lncRNA name= ref=UCSCGeneNoncode transcriptId=	7,8E-04	-46,0
TC0M000007.hg.1		BC061915 // GenBank HTC // Homo sapiens cDNA clone IMAGE:3887455, **** WARNING: chimeri	2,0E-04	-39,3
TC0M000023.hg.1		uc004cov.3 // NONCODE // accn=NULL class=lncRNA name= ref=UCSCGeneNoncode transcriptId=	1,1E-04	-37,6
TC0M000003.hg.1		S81656 // GenBank // tRNA(Ile) [human, skeletal muscle_progressive external ophtalmople	1,5E-03	-37,6
TC0M000025.hg.1		M28016 // NONCODE // accn=M28016 class=mRNAlike lncRNA name=NULL ref=H-invitational v7.	9,3E-05	-35,9
TC0M000010.hg.1		uc022bqw.1 // UCSC Genes // Homo sapiens clone 35w unknown mRNA; mitochondrial. // chrM	3,2E-06	-34,1
TC0M000009.hg.1		uc004cox.3 // NONCODE // accn=NULL class=lncRNA name= ref=UCSCGeneNoncode transcriptId=	1,5E-05	-32,7
TC0M000027.hg.1		BC017047 // NONCODE // accn=BC017047 class=mRNAlike lncRNA name=NULL ref=H-invitational	7,1E-04	-28,9
TC0M000004.hg.1		BC018860 // NONCODE // accn=BC018860 class=Non-protein coding transcript name=NULL ref=	1,8E-04	-28,3
TC01000013.hg.1		M37726 // GenBank // Human mitochondrial Lys-tRNA-aaa. // chr1 // 100 // 100 // 0 // --	3,4E-04	-25,1
TC0M000022.hg.1		BC013932 // NONCODE // accn=BC013932 class=mRNAlike lncRNA name=NULL ref=H-invitational	8,1E-04	-24,2
TC07000959.hg.1		DQ597485 // NONCODE // accn=DQ597485 class=piRNA name=piR-35551 ref=NONCODE v2.0 transc	2,8E-05	-23,4
TC01004110.hg.1		CR598056 // NONCODE // accn=CR598056 class=mRNAlike lncRNA name=Human lncRNA ref=Jounra	3,8E-05	-22,8
TC0M000024.hg.1		uc004cox.3 // NONCODE // accn=NULL class=lncRNA name= ref=UCSCGeneNoncode transcriptId=	5,1E-05	-20,7
TC01002064.hg.1		ENST00000459059 // ENSEMBL // ncrna:miRNA chromosome:GRCh37:1:566187:566265:-1 gene:ENS	4,5E-04	-18,2
TC11001412.hg.1		DQ582265 // NONCODE // accn=DQ582265 class=piRNA name=piR-32377 ref=NONCODE v2.0 transc	2,3E-03	-18,1
TC05000672.hg.1		ENST00000458878 // ENSEMBL // ncrna:miRNA chromosome:GRCh37:5:134259743:134259812:1 gen	3,8E-04	-16,4
TC0M000021.hg.1		AB019563 // NONCODE // accn=AB019563 class=mRNAlike lncRNA name=NULL ref=H-invitational	7,1E-05	-16,2
TC0M000019.hg.1		uc022bqx.1 // UCSC Genes // Sequence 933 from Patent WO2010139812. // chrM // 100 // 10	5,8E-03	-16,2
TC06000695.hg.1		ENST00000544932 // ENSEMBL // ensembl:known chromosome:GRCh38:6:61574103:61574629:1 gen	7,9E-04	-16,0
TC03002425.hg.1		TCONS_00006915 // NONCODE // accn=NULL class=lncRNA name=Human lincRNA ref=BodyMapLinc	1,8E-03	-15,3
TC0M000016.hg.1		uc022bqs.1 // UCSC Genes // Homo sapiens piRNA piR-34804, complete sequence. // chrM //	1,5E-04	-13,8
TC11001411.hg.1	MTRNR2L8	NM_001190702 // RefSeq // Homo sapiens MT-RNR2-like 8 (MTRNR2L8), mRNA. // chr11 // 100	5,0E-04	-12,9
TC02000850.hg.1		uc021vpo.1 // UCSC Genes // transfer RNA pseudogene (anticodon TTG) // chr2 // 100 // 1	3,0E-04	-10,9
TC17001269.hg.1		uc021tsy.1 // UCSC Genes // Rfam model RF00005 hit found at contig region AC131055.10/1	7,0E-04	-10,7
TC05001540.hg.1	MTRNR2L2	NM_001190470 // RefSeq // Homo sapiens MT-RNR2-like 2 (MTRNR2L2), mRNA. // chr5 // 100	3,1E-04	-10,2
TC0M000012.hg.1		DQ582201 // NONCODE // accn=DQ582201 class=piRNA name=piR-32313 ref=NONCODE v2.0 transc	5,4E-03	-9,9
TC02000740.hg.1		DQ571524 // NONCODE // accn=DQ571524 class=piRNA name=piR-31636 ref=NONCODE v2.0 transc	4,2E-02	-9,3
TC07001932.hg.1		DQ571874 // NONCODE // accn=DQ571874 class=piRNA name=piR-31986 ref=NONCODE v2.0 transc	4,2E-02	-9,3
TC01004112.hg.1		uc001abc.2 // NONCODE // accn=NULL class=lncRNA name= ref=UCSCGeneNoncode transcriptId=	1,1E-04	-9,2
TC01005191.hg.1		linc_luo_1279 // NONCODE // accn=NULL class=lincRNA name=Human lincRNA ref=Scripture Re	9,1E-06	-9,1
TC05003245.hg.1		CR623832 // NONCODE // accn=CR623832 class=mRNAlike lncRNA name=NULL ref=H-invitational	8,5E-05	-8,0
TC0M000020.hg.1		BC000845 // NONCODE // accn=BC000845 class=mRNAlike lncRNA name=NULL ref=H-invitational	2,1E-02	-8,0
TC07000373.hg.1		uc022aex.1 // UCSC Genes // Rfam model RF00005 hit found at contig region AC115220.1/10	1,6E-04	-8,0
TC01004011.hg.1		uc021plb.1 // UCSC Genes // transfer RNA pseudogene (anticodon TAA) // chr1 // 100 // 1	2,1E-05	-8,0
TC02001953.hg.1		ENST00000502349 // ENSEMBL // [retired] ncrna:lincRNA chromosome:GRCh37:2:70016684:7002	6,4E-03	-7,9
TC01004111.hg.1		CR614989 // NONCODE // accn=CR614989 class=mRNAlike lncRNA name=Human lncRNA ref=Jounra	6,8E-05	-7,8
TC03002998.hg.1		CR593869 // NONCODE // accn=CR593869 class=mRNAlike lncRNA name=NULL ref=H-invitational	2,9E-03	-7,6
TC05003112.hg.1		CR592393 // NONCODE // accn=CR592393 class=mRNAlike lncRNA name=NULL ref=H-invitational	2,1E-03	-7,5
TC0M000017.hg.1		uc022bqt.1 // UCSC Genes // Homo sapiens piRNA piR-31490, complete sequence. // chrM //	1,0E-04	-7,4
TC04001282.hg.1	PPBP	NM_002704 // RefSeq // Homo sapiens pro-platelet basic protein (chemokine (C-X-C motif)	8,0E-03	-7,1
TC0M000026.hg.1		uc004coz.1 // NONCODE // accn=NULL class=lncRNA name= ref=UCSCGeneNoncode transcriptId=	8,9E-05	-7,1
TC20000468.hg.1	TUBB1	NM_030773 // RefSeq // Homo sapiens tubulin, beta 1 class VI (TUBB1), mRNA. // chr20 //	2,6E-04	-7,0
TC04001281.hg.1	PF4	NM_002619 // RefSeq // Homo sapiens platelet factor 4 (PF4), mRNA. // chr4 // 100 // 10	1,3E-03	-6,9
TC01001619.hg.1	RGS18	NM_130782 // RefSeq // Homo sapiens regulator of G-protein signaling 18 (RGS18), mRNA.	7,5E-04	-6,7
TC10002540.hg.1		TCONS_00018467 // NONCODE // accn=NULL class=lncRNA name=Human lincRNA ref=BodyMapLinc	2,1E-03	-6,5
TC0M000011.hg.1		AF079515 // GenBank // Homo sapiens IDL RNA, complete sequence; mitochondrial. // chrM	1,3E-04	-6,2

Supplementary file 1 A, B, C, D, E, F. Top 50 RNAs with significantly changed levels in plasma EVs from patients with meningococcal septic shock (A, B), meningococcal meningitis (C, D) and systemic pneumococcal disease (E, F) when compared against the healthy control group.

RNAs with maximal signal values of < 6 (log2) across all arrays were removed to filter for low and non-expressed transcripts. The tables display transcript ID (affymetrix ID), gene symbol, mrna assignment, p-value, and Fold Change (FC) value. Lists are sorted by increased and decreased FC- values.

Nm sepsis = *Neisseria meningitidis (N. meningitidis)* septic shock patients

Nm meningitis= = *Neisseria meningitidis (N. meningitidis)* meningitis patients

Pneum = *Streptococcus pneumoniae (S. pneumoniae)* Systemic pneumococcal disease patients

Supplementary file 2 A, B, C. Heatmaps of the classification of biofunctions.

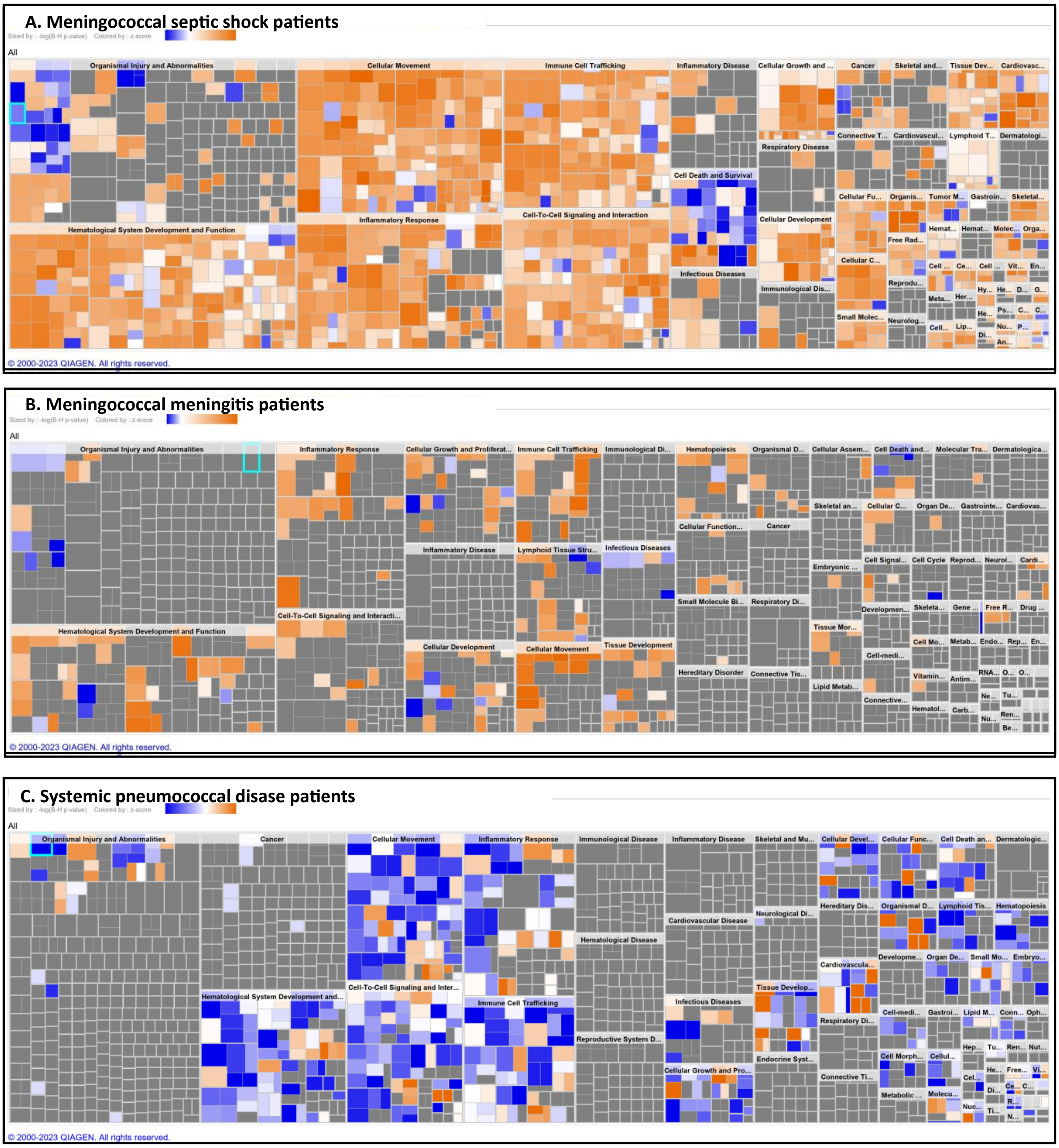

Supplementary file 2 A, B, C. Heatmaps of the classification of biofunctions predicted by Ingenuity Pathway Analysis (IPA) in EVs from plasma samples from **(A)** meningococcal septic shock -, **(B)** meningococcal meningitis-, and **(C)** systemic pneumococcal disease patients compared to the healthy control group. A ≪core analysis≫ was performed separately for each patient group The visualization is a hierarchical heat map of the functional categories in which the major boxes represent a category of related functions. Within each box, each individual rectangle is a sub-function linked to the biological function of a group of transcripts. Significantly enriched biofunctions were identified with a

right-tailed Fisher's Exact Test ($p < 0.05$, after correction for multiple testing using the Benjamini-Hochberg method. The color scheme shown is based on z-scores. Blue color or lighter shades of blue indicate a negative Z-score and down-regulation (inhibition) of the biofunction, and orange or lighter shades of orange indicate a positive Z-score and up-regulation (activation) of the biofunction. Gray color indicates no activity pattern available. White color indicates a Z-score as zero and no activity pattern identified.

Supplementary file 3. Meningococcal septic shock patients versus healthy controls.

Ingenuity Canonical Pathways	-log(B-H p-value)	Ratio	z-score
S100 Family Signaling Pathway	1.89E00	5.61E−02	3.70
Oxidative Phosphorylation	2.8E00	1.23E−01	3.05
TREM1 Signaling	6.18E00	2.22E−01	3.00
Neuroinflammation Signaling Pathway	3.66E00	9.12E−02	3.00
Dendritic Cell Maturation	1.38E00	5.97E−02	2.83
Production of Nitric Oxide and Reactive Oxygen Species in Macrophages	1.76E00	7.98E−02	2.67
Immunogenic Cell Death Signaling Pathway	2.16E00	1.19E−01	2.53
HMGB1 Signaling	1.34E00	7.55E−02	2.53
Pyroptosis Signaling Pathway	1.61E00	1E−01	2.33
Pathogen Induced Cytokine Storm Signaling Pathway	4.7E00	9.71E−02	2.26
iNOS Signaling	2.09E00	1.56E−01	2.24
IL-8 Signaling	3.01E00	9.71E−02	2.00
IL-15 Signaling	2.04E00	7.55E−02	2.00
LXR/RXR Activation	1.64E+00	8.94E−02	−2.11
Mitochondrial Dysfunction	3.22E+00	8.48E−02	−3.02

Supplementary file 3. Predicted effects on canonical pathways based on the significant difference in EV-transcript patterns in the meningococcal septic shock patients compared to healthy controls using IPA. Significantly enriched canonical pathways were identified with a right-tailed Fisher's Exact Test ($p < 0.05$), after correction for multiple testing using the Benjamini-Hochberg method. Transcripts included in the analysis were restricted to $p < 0.05$ and FC ≥|±1.5| and a Z-score =|±2|. Ratio denotes the number of significantly expressed transcripts compared with the total number of transcripts associated with the canonical pathway.

Supplementary file 4. Regulator effects in Meningococcal septic shock patients vs. healthy controls.

Consistency Score	Regulators	Target Molecules in Dataset	Diseases & Functions	Known Regulator-Disease/Function Relationship
3.750	IL1A	CCL2, CCL3, CCL4, CCL5, CXCL8, ICAM1, IL1B, IL6, NFKBIA, PTGES, RELA, S100A12, S100A8, S100A9, SERPINA1, TGFB1	Inflammatory response	100% (1/1)
3.606	IL1B	BDKRB1, CCL2, CCL3, CCL3L1, CCL4, CCL5, CCL7, CXCL8, FPR2, GNA15, IL6, S100A9, TGFB1	Quantity of Ca2	100% (1/1)
3.464	CAMP	CCL2, CCL3, CCL4, CCL5, CCL7, CXCL8, FPR2, H BEGF, ICAM1, IL1B, IL6, TGFB1	Chemotaxis	100% (1/1)
3.464	CAMP	CCL2, CCL3, CCL4, CCL5, CCL7, CXCL8, FPR2, HBEGF, ICAM1, IL1B, IL6, TGFB1	Homing of cells	100% (1/1)
3.464	IL17A	CCL2, CCL7, CXCL8, HBEGF, ICAM1, IL1B, IL6, MMP8, S100A12, S100A8, S100A9, STAT3	Angiogenesis	100% (1/1)
3.464	NFKB (complex)	CCL2, CCL3, CCL3L1, CCL4, CCL5, CCL7, CXCL8, FPR2, IL1B, IL6, PLAUR, TGFB1	Chemotaxis of myeloid cells	0% (0/1)
3.333	IL1B	ANXA1, CCL2, CCL3, CCL3L1, CCL4, CCL5, CCL7, CXCL8, FPR2	Attraction of mononuclear leukocytes	100% (1/1)
3.333	RELA	CCL2, CCL3, CCL5, CXCL8, HIF1A, ICAM1, IL1B, I L6, TGFB1	Adhesion of mononuclear leukocytes	100% (1/1)
3.328	CAMP	CCL2, CCL3, CCL4, CCL5, CCL7, CXCL8, FPR2, HBEGF, ICAM1, IL1B, IL6, TGFB1, TNFAIP3	Cell movement	100% (1/1)
3.328	CAMP	CCL2, CCL3, CCL4, CCL5, CCL7, CXCL8, FPR2, HBEGF, ICAM1, IL1B, IL6, TGFB1, TNFAIP3	Migration of cells	100% (1/1)
3.328	TLR4	CCL2, CCL3, CCL4, CCL5, CXCL8, HK2, ICAM1, IL1B, IL6, NFKBIA, RELA, SOD2, XBP1	Cell movement	100% (1/1)

3.328	TLR4	CCL2, CCL3, CCL4, CCL5, CXCL8, HK2, ICAM1, IL1B, IL6, NFKBIA, RELA, SOD2, XBP1	Migration of cells	
3.317	CAMP	CCL2, CCL3, CCL4, CCL5, CCL7, CXCL8, FPR2, ICAM1, IL1B, IL6, TGFB1	Cell movement of mononuclear leukocytes	100% (1/1)
3.250	JNK (family)	CCL2, CCL3, CCL4, CCL5, CXCL8, DUSP1, FOSB, ICAM1, IL1B, IL6, NFE2L2, PLAUR, PTEN, S100A12, SOD2, TGM2	Cell movement	100% (1/1)
3.182	IL1B	CCL2, CCL3L1, CCL5, CXCL8, HIF1A, ICAM1, RELA, TGFB1	Adhesion of monocytes	100% (1/1)
3.182	MAPK14	CCL2, CXCL8, IL1B, IL6, PLAUR, S100A8, S100A9, TGFB1	Chemotaxis of granulocytes	100% (1/1)
3.182	RELA	CCL2, CCL3, CCL5, CXCL8, HIF1A, ICAM1, IL1B, TGFB1	Binding of monocytes	100% (1/1)
3.175	CAMP	CCL2, CCL3, CCL4, CCL5, CCL7, CXCL8, FPR2, ICAM1, IL1B, IL6, TGFB1, TNFAIP3	Cell movement of blood cells	100% (1/1)
3.175	CAMP	CCL2, CCL3, CCL4, CCL5, CCL7, CXCL8, FPR2, ICAM1, IL1B, IL6, TGFB1, TNFAIP3	Cell movement of myeloid cells	100% (1/1)
3.175	CAMP	CCL2, CCL3, CCL4, CCL5, CCL7, CXCL8, FPR2, ICAM1, IL1B, IL6, TGFB1, TNFAIP3	Cell movement of phagocytes	100% (1/1)
3.175	IFNG	CCL2, CCL3, CCL4, CCL5, CCL7, CXCL16, CXCL8, FCGR1A, FCGR2A, FPR2, PTAFR, SELL	Mobilization of Ca2	100% (1/1)
3.162	CAMP	CCL2, CCL3, CCL4, CCL5, CCL7, CXCL8, ICAM1, IL1B, IL6, TGFB1	Migration of mononuclear leukocytes	100% (1/1)
3.162	ERK1/2 (family)	CCL2. CCL3. CCL4. CCL5. CCL7. CXCL8. ICAM1. IL1B. IL6. PLAUR	Migration of mononuclear leukocytes	100% (1/1)
3.162	JNK (family)	CCL2. CCL3. CCL4. CCL5. CXCL8. ICAM1. IL1B. IL6. PLAUR. TGM2	Migration of mononuclear leukocytes	100% (1/1)
3.098	TNF	BDKRB1. CCL2. CCL3. CCL4. CCL5. CCL7. CXCL8. GNA15. IL1B. IL6. PLAUR. PROK2. S100A8. S100A9. TGFB1	Quantity of Ca2	100% (1/1)

3.051	JNK (family)	CCL2. CCL3. CCL4. CCL5. D2. TGM2	Cell movement of tumor cell lines	100% (1/1)
3.024	CCL5	CCL2. CCL3. CCL4. CXCL8. IL1B. IL6. STAT3	Proinflammatory response	100% (1/1)
3.024	MAPK14	CCL2. CXCL8. IL1B. IL6. S100A8. S100A9. TGFB1	Chemotaxis of neutrophils	100% (1/1)

Supplementary file 4. Regulator effects in Meningococcal septic shock patients vs. healthy controls. The Regulator Effects algorithm connects upstream regulators. dataset molecules and downstream functions affected in the dataset to generate a hypothesis that can explain how the activation or inhibition of an upstream regulator affects the downstream target molecule expression and the impact of the molecular expression on functions. The algorithm goes through one or more iterations to merge upstream and downstream results from the Upstream Regulator. The networks are merged only if the overlap of targets has possible statistical significance (Fisher's Exact Test p-value of <0.05). For each network, a Consistency Score is calculated that rewards for paths from regulator->target->disease or function that are consistent. Higher scoring hypotheses are those with more consistent causal paths represented by a high Consistency Score.

Definition of column names:

Consistency Score: is a measure of how causally consistent and densely connected a Regulator Effects network is.

Regulators: list of regulators participating in the network.

Targets: list of the targets in the network.

Diseases and Functions: number of functions and diseases in the network.

Known Regulator-Disease-Function Relationship: Percent of possible regulator to disease/function relationships that are already known.

Reference for the explanation of the regulator effects algorithm in the text of supplementary file 4 text: Qiagen White paper Regulator Effects in IPA®

Supplementary file 5.

Symbol	Expr p-value	Expr Fold Change	Location	Family
CCL2	0.044	3.84	Extracellular Space	cytokine
CCL3	0.041	2.00	Extracellular Space	cytokine
CCL4	0.030	3.72	Extracellular Space	cytokine
CCL5	0.000	-3.21	Extracellular Space	cytokine
CXCL8	0.026	6.28	Extracellular Space	cytokine
ICAM1	0.043	3.75	Plasma Membrane	transmembrane receptor
IL1A	0.310	1.74	Extracellular Space	cytokine
IL1B	0.011	4.55	Extracellular Space	cytokine
IL6	0.038	2.01	Extracellular Space	cytokine
Inflammatory response			Other	function
NFKBIA	0.036	3.29	Cytoplasm	transcription regulator
PTGES	0.002	1.76	Cytoplasm	enzyme
RELA	0.009	2.20	Nucleus	transcription regulator
S100A12	0.001	9.70	Cytoplasm	other
S100A8	0.000	3.48	Cytoplasm	other
S100A9	0.001	8.38	Cytoplasm	other
SERPINA1	0.001	3.52	Extracellular Space	other
TGFB1	0.047	-1.84	Extracellular Space	growth factor

Supplementary file 5. Data obtained from a "core-analysis" in IPA (Figure 5A). Transcripts, upstream regulators and biofunctions involved in regulator effect network analysis in meningococcal septic shock patients versus healthy controls for the network with the highest consistency score (see Supplementary file 4).

Supplementary file 6. Meningococcal meningitis patients versus healthy controls.

Ingenuity Canonical Pathways	-log(B-H p-value)	Ratio	z-score
Neuroinflammation Signaling Pathway	1.80E+00	1.95E−02	2.2
Production of Nitric Oxide and Reactive Oxygen Species in Macrophages	1.39E+00	2.13E−02	2

Supplementary file 6. Predicted effects on canonical pathways based on the significant difference in EV-transcript patterns in the meningococcal meningitis patients compared to healthy controls using IPA. Significantly enriched canonical pathways were identified with a right-tailed Fisher's Exact Test ($p < 0.05$), after correction for multiple testing using the Benjamini-Hochberg method. Transcripts included in the analysis were restricted to $p < 0.05$ and FC ≥|±1.5| and a Z-score =|±2|. Ratio denotes the number of significantly expressed transcripts compared with the total number of transcripts associated with the canonical pathway.

Supplementary file 7. Regulator effects in Meningococcal meningitis patients vs. healthy control.

Consistency score	Regulator	Target molecule in dataset	Diseases and function	Known regulator
10.607	Immunglobulin	AQP9. CXCR2. CYBA. FPR1. IL1B. S100A12. S100A8. S100A9	Adhesion of immune cells. Cell movement	100% (5/5)
-2.214	TNF	AQP9. CLEC4E. CXCR2. CYBA. IL1B. LY96.S100A12. S100A8. S100A9. TREM1	Inflammatory response	100% (1/1)

Supplementary file 7. Regulator effects in Meningococcal meningitis patients vs. healthy control.
Table text see Supplementary file 4.

Supplementary file 8.

Symbol	Expr p-value	Expr Fold Change	Location	Family
Adhesion of immune cells			Other	function
AQP9	0.001	3.87	Plasma Membrane	transporter
Cell movement of myeloid cells			Other	function
Cell movement of phagocytes			Other	function
Chemotaxis			Other	function
CXCR2	0.014	7.00	Plasma Membrane	G-protein coupled receptor
CYBA	0.040	1.56	Cytoplasm	enzyme
FPR1	0.037	2.12	Plasma Membrane	G-protein coupled receptor
IL1B	0.033	1.80	Extracellular Space	cytokine
Immunoglobulin			Extracellular Space	complex
Inflammatory response			Other	function
S100A12	0.041	5.41	Cytoplasm	other
S100A8	0.029	2.46	Cytoplasm	other
S100A9	0.016	4.20	Cytoplasm	other

Supplementary file 8. Data obtained from a “core-analysis” in IPA (Figure 5B). Transcripts, upstream regulators and biofunctions involved in regulator effect network analysis in meningococcal meningitis patients versus healthy controls for the network with the highest consistency score (Supplementary file 7).

Supplementary file 9. Systemic pneumococcal disease patients versus healthy controls.

Ingenuity Canonical Pathways	-log (B-H p-value)	Ratio	z-score
Pyroptosis Signaling Pathway	1.33E00	5.32E−02	2.24
RHOGDI Signaling	1.97E00	4.55E−02	2
Gap junction trafficking and regulation	1.93E00	1.18E−01	−2.00
Syndecan interactions	2.2E00	1.48E−01	−2.00
G alpha (s) signalling events	5.44E−01	2.76E−02	−2.00
RHO GTPases activate IQGAPs	2.03E00	1.29E−01	−2.00
RHO GTPases activate PAKs	2.47E00	1.9E−01	−2.00
RUNX1 regulates megakaryocyte differentiation and platelet function	1.93E00	8.62E−02	−2.00
Kinesins	1.32E00	6.56E−02	−2.00
α-Adrenergic Signaling	4.33E00	1.01E−01	−2.00
Renin-Angiotensin Signaling	1.44E00	4.96E−02	−2.00
Thrombin Signaling	1.58E00	4E−02	−2.00
CDK5 Signaling	1.94E00	6.09E−02	−2.00
Melanocyte Development and Pigmentation Signaling	1.75E00	6.12E−02	−2.00
IL-1 Signaling	4.13E00	1.04E−01	−2.00
Ephrin B Signaling	1.66E00	6.94E−02	−2.00
GADD45 Signaling	1.34E00	6.67E−02	−2.00
Gαs Signaling	2.72E00	7.14E−02	−2.00
Cardiac β-adrenergic Signaling	2.42E00	5.56E−02	−2.00
Endocannabinoid Developing Neuron Pathway	2.26E00	6.3E−02	−2.00
Apelin Endothelial Signaling Pathway	2.49E00	6.38E−02	−2.00
PFKFB4 Signaling Pathway	2.18E00	1.02E−01	−2.00
Opioid Signaling Pathway	3.87E00	5.71E−02	−2.11
IL-8 Signaling	1.73E00	4.29E−02	−2.12
Dopamine-DARPP32 Feedback in cAMP Signaling	1.57E00	4.3E−02	−2.12
ABRA Signaling Pathway	1.36E00	5.43E−02	−2.24
Formation of Fibrin Clot (Clotting Cascade)	2.45E00	1.28E−01	−2.24
Vasopressin regulates renal water homeostasis via Aquaporins	2.3E00	1.16E−01	−2.24
Amyloid fiber formation	1.64E00	6.85E−02	−2.24
CXCR4 Signaling	2.57E00	5.95E−02	−2.24
Melatonin Signaling	1.66E00	6.94E−02	−2.24
Calcium Signaling	1.62E00	4.09E−02	−2.24
G alpha (i) signalling events	2.35E00	4.65E−02	−2.31
Translocation of SLC2A4 (GLUT4) to the plasma membrane	4.24E00	1.27E−01	−2.33
Opioid Signalling	1.88E00	6.67E−02	−2.45
MHC class II antigen presentation	1.39E00	4.76E−02	−2.45
L1CAM interactions	1.39E00	4.8E−02	−2.45
Platelet homeostasis	1.93E00	6.98E−02	−2.45
Interleukin-4 and Interleukin-13 signaling	1.57E00	5.45E−02	−2.45
Coronavirus Replication Pathway	2.94E00	1.33E−01	−2.45
Cardiac Hypertrophy Signaling (Enhanced)	2.7E00	3.69E−02	−2.50
Role of NFAT in Cardiac Hypertrophy	3.23E00	5.8E−02	−2.53
Adrenomedullin signaling pathway	2.21E00	5.03E−02	−2.53
Activation of NMDA receptors and postsynaptic events	2.49E00	8.33E−02	−2.65
Smooth Muscle Contraction	3.87E00	1.63E−01	−2.65
GPER1 signaling	3.82E00	1.56E−01	−2.65
Corticotropin Releasing Hormone Signaling	1.93E00	5.26E−02	−2.65
Ephrin Receptor Signaling	1.42E00	3.96E−02	−2.65
Gap Junction Signaling	3.78E00	5.23E−02	−2.67
Cardiac Hypertrophy Signaling	3.72E00	5.75E−02	−2.71
Actin Cytoskeleton Signaling	1.78E00	4.1E−02	−2.83
G Beta Gamma Signaling	2.23E00	6.2E−02	−2.83
Eicosanoid Signaling	2.18E00	4.29E−02	−2.89
SNARE Signaling Pathway	2.57E00	6.62E−02	−3.00
Orexin Signaling Pathway	1.47E00	3.78E−02	−3.00
Phospholipase C Signaling	1.84E00	2.5E−02	−3.00
Estrogen Receptor Signaling	2.17E00	3.67E−02	−3.05
Serotonin Receptor Signaling	3.4E00	4.26E−02	−3.13
Response to elevated platelet cytosolic Ca2+	6.51E00	1.14E−01	−3.36
Oxytocin Signaling Pathway	2.49E00	4.61E−02	−3.61

Supplementary file 9. Predicted effects on canonical pathways based on the significant difference in EV-transcript patterns in the systemic pneumococcal disease patients compared to healthy controls using Ingenuity Pathway analysis. Significantly enriched canonical pathways were identified with a right-tailed Fisher's Exact Test ($p < 0.05$), after correction for multiple testing using the Benjamini-Hochberg method. Transcripts included in the analysis were restricted to $p < 0.05$ and FC $\geq|\pm1.5|$ and a Z-score $=|\pm2|$. Ratio denotes the number of transcripts significantly expressed compared with the total number of transcripts associated with the canonical pathway.

Supplementary file 10. Regulator effects in pneumococcal sepsis patients vs. healthy control.

Consistency Score	Regulators	Target Molecules in Dataset	Diseases & Functions	Known Regulator-Disease/Function Relationship
13.005	miR-30c-5p (and other miRNAs w/seed GUAAACA). MRTFA. MRTFB. SRF. VIPAS39	CAVIN2. CCL5. DAB2. FLNA. FOXO3. GNAI2. GP1BA. ITGB1. MYH9. MYL9. PF4. PPBP. PPP3R1. RAB27B. RAP1B. S100A8. S100A9. SPARC. THBS1. TLN1. TUBB1. VCL	Aggregation of blood platelets. Bleeding. Cell viability. Hemorrhagic disease. Non-malignant disorder	32% (8/25)
8.660	AR. FN1. GATA1. MYO6	CCL5. ELMO1. FLNA. GNAS. GP1BA. GUCY1B1. IL1B. ITGB3. PF4. TGFB1. THBS1. TLN1	Activation of blood platelets. Cell movement of natural killer cells. Chemotaxis of mononuclear leukocytes. Disassembly of focal adhesions. Homing of lymphocytes	15% (3/20)
8.000	NFATC1. PRKG1. TGF beta	CCL5. CLU. DAB2. GUCY1A1. GUCY1B1. IL1B. ITGB1. ITGB3. MAX. MXI1. PPP3R1. SPARC. TBX21. TCF3. TGFB1. THBS1	Cell death of kidney cells. Cell spreading. Inflammation of lung. Organismal death	33% (4/12)
6.325	IZUMO1R. MRGPRX3. SKIC2. TYK2	B2M. CD3E. CLEC4E. CSF3R. ENO1. IL1B. IL23A. MTHFD2. S100A8. S100A9	Flux of Ca2. Inflammation of body cavity. Inflammation of gastrointestinal tract. Inflammation of organ. Occlusion of artery. Sepsis	21% (5/24)
4.536	AR	CCL5. ELMO1. GUCY1A1. PTGS1. TGFB1. THBS1. VCL	Aggregation of cells. Chemotaxis of mononuclear leukocytes. Homing of lymphocytes	0% (0/3)
3.015	GATA1	CCL5. GNAS. GP1BA. GUCY1B1. HBA1/HBA2. HBB. ITGB3. PF4. SNCA. TGM2. TUBB1	Activation of cells. Anemia. Bleeding time. Chemotaxis of natural killer cells	50% (2/4)
2.425	CEBPA. ERG.TGF beta.	CCL3. CCL5. CSF3R. EOMES. F13A1. FLNA. ITGB1. ITGB3. MYH9. PTAFR. PTGS1. S100A9. SERPINA1. SPARC. TFPI. THBS1. YWHAZ	Cell spreading of endothelial cells.	0% (0/8)

	TGFB1		Hemorrhagic disease.	
1.633	NFATC1	CCR9. IL1B. ITGB3. PPP3R1. TBX21. THBS1	Inflammation of respiratory system component. Viral Infection	50% (1/2)
1.134	CHROMR. CSF	BCL2A1. GCA. IFITM1. IFITM3. IL1B. IL1R2. TGM2	Sepsis	0% (0/2)
1.061	CCN2. SELPLG	BCL2A1. CAVIN2. CCL3. ENO1. IL1B. IL1R2. SPARC. TGFB1	Inflammation of organ	50% (1/2)
−2.598	MRTFB	CCL5. DAB2. FLNA. ITGB1. MYH9. MYL9. RAB27B. S100A8. S100A9. THBS1. TUBB1. VCL	Organization of cytoskeleton	100% (1/1)
−3.402	FN1	CCL5. CXCL5. IL1B. ITGB1. ITGB3. TGFB1. TLN1	Leukopoiesis	100% (1/1)
−4.899	GATA1	BMP6. FECH. GNAS. HBA1/HBA2. ITGB3. TGM2	Proliferation of neuronal cells	0% (0/1)
−6.124	TGFB1	B2M. FOXO3. IL1B. PTGS1. TBX21. TGM2	Septic shock	100% (1/1)
−7.000	NLRX1	DAB2. GNA12. IL1B. ITGB1	Aggregation of cells	0% (0/1)
−22.500	TGF beta	CCL3. CCL5. TBX21. TCF3	Production of cells	100% (1/1)

Supplementary file 10. Regulator effects in pneumococcal sepsis patients vs. healthy control. Table text see Supplementary file 4.

Supplementary file 11.

Symbol	Expr p-value	Expr Fold Change	Location	Family
Aggregation of blood platelets			Other	function
Bleeding			Other	disease
CAVIN2	0.001	-4.34	Plasma Membrane	other
CCL5	0.000	-2.81	Extracellular Space	cytokine
Cell viability			Other	function
DAB2	0.034	-3.44	Plasma Membrane	other
FLNA	0.003	-1.90	Cytoplasm	other
FOXO3	0.002	-2.09	Nucleus	transcription regulator
GNAI2	0.016	-1.51	Plasma Membrane	enzyme
GP1BA	0.033	-1.60	Plasma Membrane	transmembrane receptor
Hemorrhagic disease			Other	disease
ITGB1	0.003	-1.88	Plasma Membrane	transmembrane receptor
miR-30c-5p (and other miRNAs w/seed GUAAACA)			Cytoplasm	mature microRNA
MRTFA			Nucleus	transcription regulator
MRTFB			Nucleus	transcription regulator
MYH9	0.038	-2.06	Cytoplasm	enzyme
MYL9	0.002	-2.54	Cytoplasm	other
Non-malignant disorder			Other	function
PF4	0.001	-6.90	Extracellular Space	cytokine
PPBP	0.008	-7.14	Extracellular Space	cytokine
PPP3R1	0.017	-1.91	Cytoplasm	phosphatase
RAB27B	0.000	-4.41	Cytoplasm	enzyme
RAP1B	0.043	-1.90	Cytoplasm	enzyme
S100A8	0.001	2.75	Cytoplasm	other
S100A9	0.013	3.95	Cytoplasm	other
SPARC	0.014	-3.15	Extracellular Space	other
SRF	0.407	1.07	Nucleus	transcription regulator
THBS1	0.004	-2.23	Extracellular Space	other
TLN1	0.046	-1.70	Plasma Membrane	other
TUBB1	0.000	-6.97	Cytoplasm	other
VCL	0.002	-2.18	Plasma Membrane	other
VIPAS39			Plasma Membrane	other

Supplementary file 11. Data obtained from a "core-analysis" in IPA (Figure 5C). Transcripts, upstream regulators and biofunctions involved in regulator effect network analysis in systemic pneumococcal disease patients versus healthy controls for the network with the highest consistency score (see Supplementary file 10).

Supplementary file 12. Predicted effects on biofunctions.

	Z-score	Z-score	Z-score
**Diseases and Bio Functions**	Nm sep vs. Ctr	Nm men vs. Ctr	Pneum sep vs. Ctr
Cell movement	4,77	3,51	-0,30
Migration of cells	4,49	3,36	-0,18
Angiogenesis	4,29	2,25	1,31
Inflammatory response	3,09	3,07	-0,96
Organismal death	1,94	N/A	4,78
Vasculogenesis	3,92	1,96	0,69
Chemotaxis	2,91	2,47	-1,06
Proliferation of vascular cells	2,84	1,89	1,68
Growth of epithelial tissue	2,80	1,57	1,42
Inflammatory response of cells	2,35	1,94	1,41
Chemotaxis of neutrophils	2,45	2,39	0,66
Quantity of Ca2+	2,27	1,98	1,20
Immune response of cells	2,87	1,56	-1,00
Cell movement of mononuclear leukocytes	2,03	2,22	-1,15
Development of epithelial tissue	2,41	1,17	1,82
Proliferation of endothelial cells	2,35	1,17	1,80
Leukocyte migration	1,92	2,61	-0,62
Cell proliferation of tumor cell lines	4,36	N/A	-0,76
Cell movement of myeloid cells	2,67	2,28	0,16
Cell movement of leukocytes	1,84	2,41	-0,68
Invasion of cells	3,84	N/A	-0,97
Cell movement of phagocytes	2,10	2,14	-0,48
Cell viability	2,68	N/A	-2,03
Mobilization of Ca2+	2,80	1,02	0,85
Cell survival	2,79	N/A	-1,85
Occlusion of artery	2,45	N/A	2,17
Immune response of leukocytes	2,56	1,46	-0,60
Activation of myeloid cells	2,02	1,55	-0,97
Invasion of tumor cell lines	3,57	N/A	-0,71
Apoptosis	-3,02	-1,02	-0,23
Cell movement of tumor cell lines	4,08	N/A	0,15
Leukopoiesis	0,42	1,65	-2,14
Homing of cells	2,99	N/A	-1,10
Viral Infection	1,81	-0,17	-2,08
Adhesion of immune cells	1,61	2,41	0,01

Supplementary file 12. Predicted effects on biofunctions based on EV-RNA patterns (Figure 7 A) in plasma samples from patients with meningococcal septic shock (denoted Nm sep), meningococcal meningitis (denoted Nm men), and systemic pneumococcal disease patients (denoted Pneum sep) versus healthy controls. The table is based on the highest z-score values in the Nm sepsis group versus control group.

Supplementary file 13. Predicted effects on canonical pathways.

	Z-score	Z-score	Z-score
**Canonical Pathways**	Nm sep vs. Ctr	Nm men vs. Ctr	Pneum sep vs. Ctr
S100 Family Signaling Pathway	3,70	1,67	0,89
Production of Nitric Oxide and Reactive Oxygen Species in Macrophages	2,67	2,00	1,34
Neuroinflammation Signaling Pathway	3,00	2,24	0,38
Neutrophil Extracellular Trap Signaling Pathway	1,92	2,24	-1,39
Pathogen Induced Cytokine Storm Signaling Pathway	2,26	1,63	-1,16
Actin Cytoskeleton Signaling	-1,89	N/A	-2,83
Pyroptosis Signaling Pathway	2,33	N/A	2,24
Mitochondrial Dysfunction	-3,02	N/A	-1,51
Coronavirus Replication Pathway	-1,89	N/A	-2,45
Dendritic Cell Maturation	2,83	1,00	-0,45
SNARE Signaling Pathway	-1,16	N/A	-3,00
IL-8 Signaling	2,00	N/A	-2,12
Relaxin Signaling	-1,41	N/A	-2,65
Oxytocin Signaling Pathway	-0,45	N/A	-3,61
GADD45 Signaling	-1,89	N/A	-2,00
G Beta Gamma Signaling	-1,00	N/A	-2,83
Role of NFAT in Regulation of the Immune Response	0,78	N/A	-2,83
HMGB1 Signaling	2,53	N/A	-1,00
Phospholipase C Signaling	-0,50	N/A	-3,00
Adrenomedullin signaling pathway	0,83	N/A	-2,53
Response to elevated platelet cytosolic Ca2+	N/A	N/A	-3,36
Corticotropin Releasing Hormone Signaling	-0,71	N/A	-2,65
Cardiac β-adrenergic Signaling	-1,34	N/A	-2,00
Cardiac Hypertrophy Signaling (Enhanced)	0,82	N/A	-2,50
Tumor Microenvironment Pathway	2,84	N/A	-0,45
CXCR4 Signaling	-1,00	N/A	-2,24
Apelin Endothelial Signaling Pathway	1,13	N/A	-2,00
IL-1 Signaling	1,13	N/A	-2,00
Serotonin Receptor Signaling	N/A	N/A	-3,13
Colorectal Cancer Metastasis Signaling	0,63	N/A	-2,45
Melatonin Signaling	-0,82	N/A	-2,24
Oxidative Phosphorylation	3,05	N/A	N/A
Estrogen Receptor Signaling	0,00	N/A	-3,05
Orexin Signaling Pathway	N/A	N/A	-3,00
Thrombin Signaling	1,00	N/A	-2,00
TREM1 Signaling	3,00	N/A	N/A
Cardiac Hypertrophy Signaling	0,26	N/A	-2,71
Ephrin Receptor Signaling	0,30	N/A	-2,65
Calcium Signaling	-0,71	N/A	-2,24
Opioid Signaling Pathway	-0,83	N/A	-2,11
LXR/RXR Activation	-2,11	N/A	-0,82
Eicosanoid Signaling	N/A	N/A	-2,89
Role of IL-17F in Allergic Inflammatory Airway Diseases	2,83	N/A	N/A
Gαs Signaling	-0,82	N/A	-2,00
CDK5 Signaling	-0,82	N/A	-2,00
RHOGDI Signaling	0,82	N/A	2,00

Supplementary file 13. Predicted effects on canonical pathways based on EV-RNA patterns (Figure 7 B) in plasma samples from patients with meningococcal septic shock (denoted Nm sep), meningococcal meningitis (denoted Nm men), and systemic pneumococcal disease patients (denoted Pneum sep) versus healthy controls. The table is based on the highest z-score values in the Nm sepsis group versus control group.

Supplementary file 14. Predicted effects on upstream regulators.

Upstream Regulators	Z-score Nm sep vs. Ctr	Z-score Nm men vs. Ctr	Z-score Pneum sep vs. Ctr
TNF	5,45	3,04	1,27
IFNG	4,46	3,06	0,91
TGM2	3,64	1,63	1,81
Immunoglobulin	-2,06	-3,60	-0,80
CG	4,21	2,00	N/A
CSF	3,85	N/A	2,24
IL6	3,50	1,47	0,99
IL17A	3,61	1,44	0,86
IL1A	3,29	2,00	0,32
CEBPA	3,45	N/A	2,11
STAT1	2,28	1,96	0,98
EGFR	3,01	N/A	2,00
IL5	3,64	N/A	1,36
SELPLG	3,00	N/A	2,00
OSM	2,91	1,46	0,56
SMARCA4	2,01	1,66	1,18
TGFB1	2,14	N/A	-2,63
CD3	3,87	0,56	-0,29
F2	3,21	N/A	-1,47
IFNA2	2,10	1,29	1,15
CD40	3,91	N/A	0,59
GATA1	-1,80	N/A	-2,57
ERG	-2,16	N/A	-2,20
HMGB1	3,10	N/A	1,21
TLR4	2,89	0,34	1,01
IL4	2,72	0,89	-0,63
ERBB2	2,80	N/A	1,41
IL22	2,76	N/A	1,39
SELP	3,00	N/A	1,15
CSF2	3,48	N/A	0,63
NFkB (complex)	3,77	N/A	0,25
DDX5	2,61	N/A	1,40
VIPAS39	-2,00	N/A	-2,00
BCR (complex)	3,58	N/A	-0,38
IL1B	3,73	N/A	0,22

Supplementary file 14. Predicted effects on upstream regulators based on EV-RNA patterns (Figure 7 C) in plasma samples from patients with meningococcal septic shock (denoted Nm sep), meningococcal meningitis (denoted Nm men), and systemic pneumococcal disease patients (denoted Pneum sep) versus healthy controls. The table is based on the highest z-score values in the Nm sepsis group versus control group.

Supplementary file 15. Predicted effects on gene signaling pathways from the top up-regulated biofunctions based on EV-RNA patterns in plasma samples from patients with meningococcal septic shock vs. healthy controls, meningococcal meningitis vs. healthy controls, and systemic pneumococcal disease vs. healthy controls.

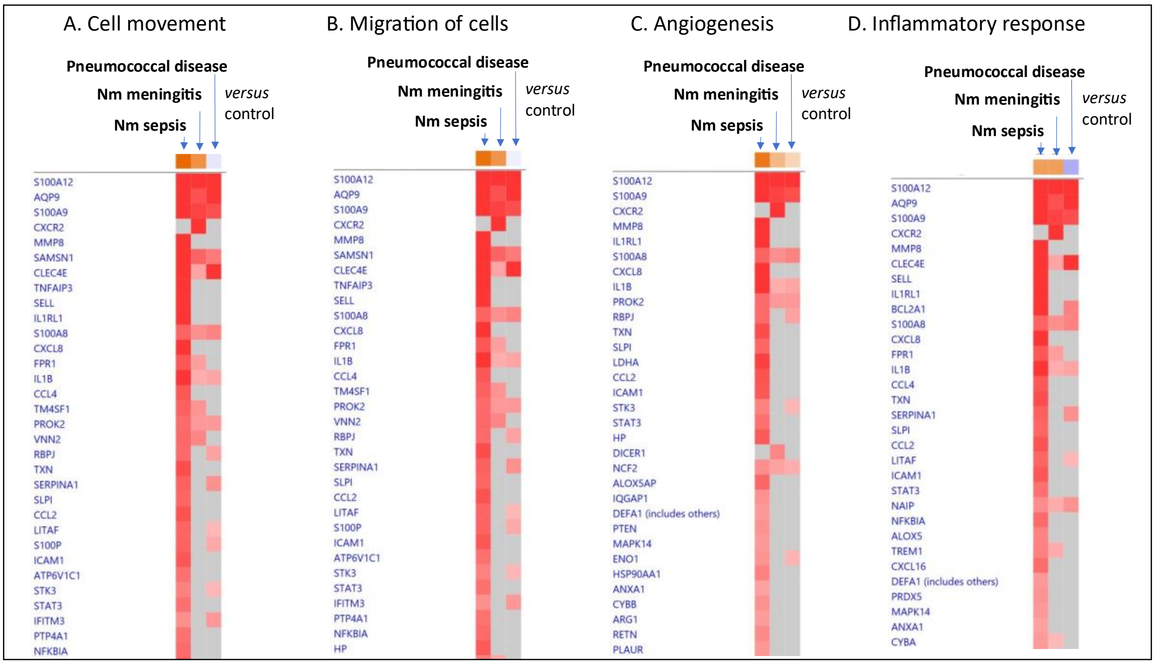

Supplementary file 15. Predicted effects on gene signaling pathways from the top up-regulated biofunctions based on EV-RNA patterns in plasma samples from patients with meningococcal septic shock vs. healthy controls, meningococcal meningitis vs. healthy controls, and systemic pneumococcal disease vs. healthy controls. The Z-score indicates predicted activation state of canonical pathways. Orange or lighter shades of orange indicate a positive Z-score and up-regulation of the pathway. Blue color or lighter shades of blue indicate a negative Z-score and down-regulation of the pathway. The transcripts in the gene signaling pathway are expressed as Fold Change (FC) values. Red or lighter shades of red indicates positive FC-values and up-regulation of transcripts. Green color or lighter shades of green indicates negative FC- values and down-regulation of transcripts. Color gray indicates that a predicted activation state of a gene/transcript in the canonical pathway signaling network is not affected. Note that only the top biofunctions are shown.

Supplementary file 16. Predicted effects on gene signaling pathways from the top up-regulated canonical pathways based on EV-RNA patterns in plasma samples from patients with meningococcal septic shock vs. controls, meningococcal meningitis vs. controls, and systemic pneumococcal disease vs. controls.

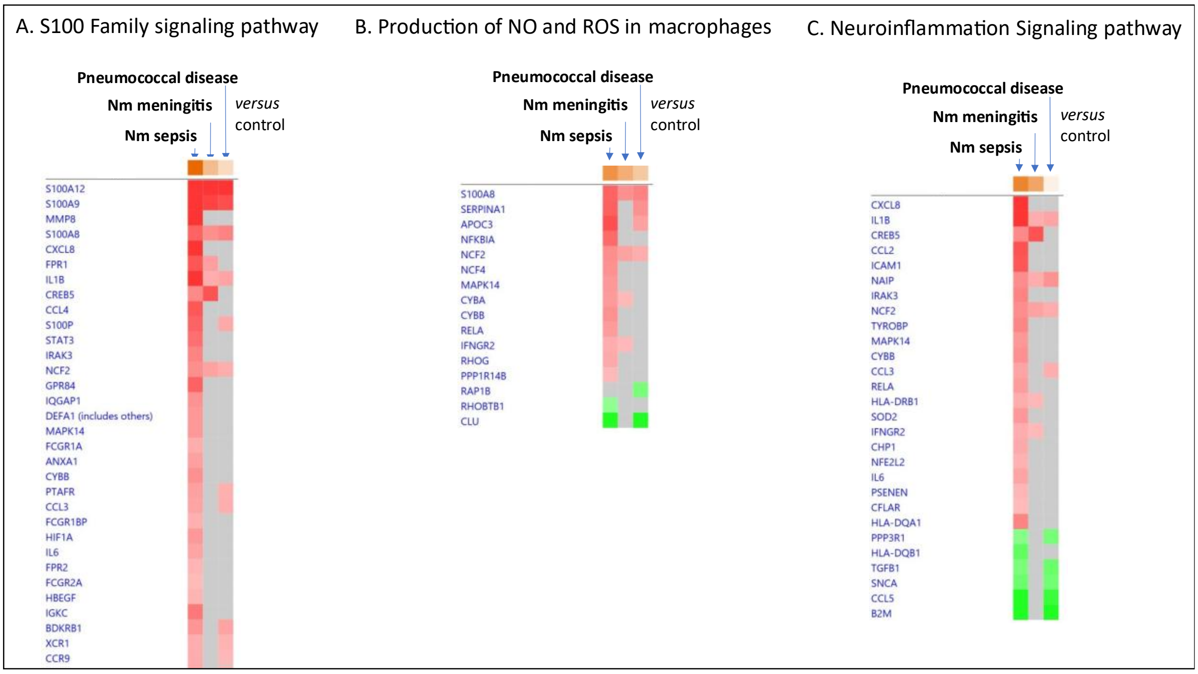

Supplementary file 16. Predicted effects on gene signaling pathways from the top up-regulated canonical pathways based on EV-RNA patterns in plasma samples from patients with meningococcal septic shock vs. controls, meningococcal meningitis vs. controls, and systemic pneumococcal disease vs. controls. The Z-score indicates predicted activation state of canonical pathways. Orange or lighter shades of orange indicate a positive Z-score and up-regulation of the pathway. Blue color or lighter shades of blue indicate a negative Z-score and down-regulation of the pathway. The transcripts in the gene signaling pathway are expressed as Fold Change (FC) values. Red or lighter shades of red indicates positive FC-values and up-regulation of transcripts. Green color or lighter shades of green indicates negative FC- values and down-regulation of transcripts. Color gray indicates that a predicted activation state of a gene/transcript in the canonical pathway signaling network is not affected. Note that only the top pathways are shown.

Supplementary file 17. Predicted effects on gene signaling pathways from the top up-regulated and down-regulated Upstream regulators based on EV-RNA patterns in plasma samples from patients with meningococcal septic shock vs. healthy controls, meningococcal meningitis vs. healthy controls, and systemic pneumococcal disease vs. healthy controls.

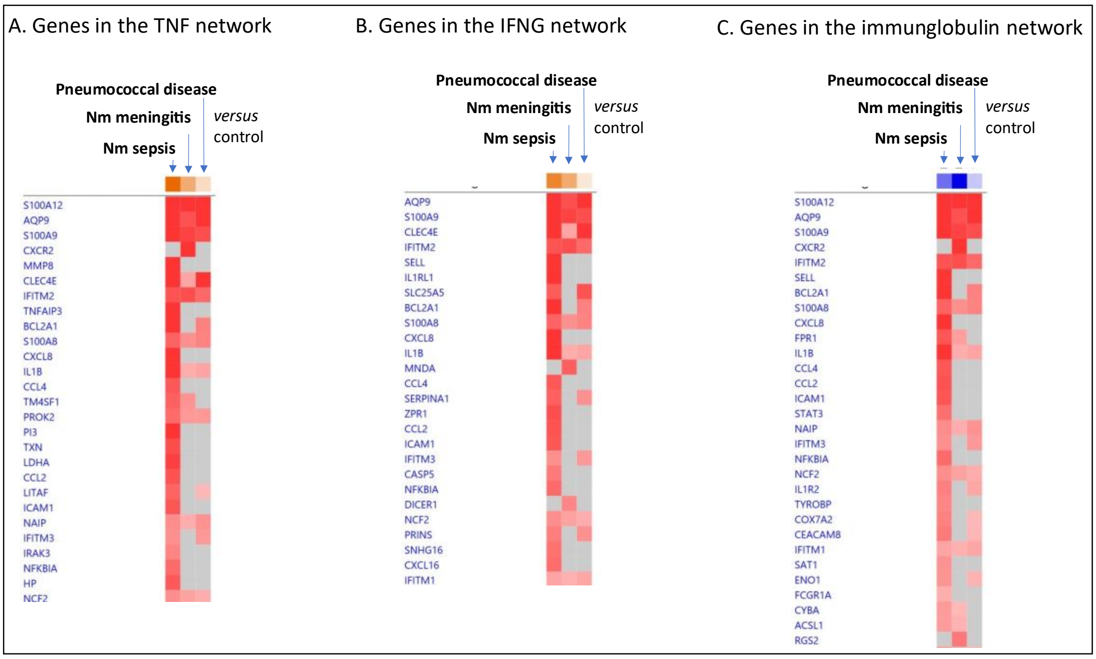

Supplementary file 17. Predicted effects on gene signaling pathways from the top up-regulated and down-regulated Upstream regulators based on EV-RNA patterns in plasma samples from patients with meningococcal septic shock vs. healthy controls, meningococcal meningitis vs. healthy controls, and systemic pneumococcal disease vs. healthy controls. The Z-score indicates predicted activation state of canonical pathways. Orange or lighter shades of orange indicate a positive Z-score and up-regulation of the pathway. Blue color or lighter shades of blue indicate a negative Z-score and down-regulation of the pathway. The transcripts in the gene signaling pathway are expressed as Fold Change (FC) values. Red or lighter shades of red indicates positive FC-values and up-regulation of transcripts. Green color or lighter shades of green indicates negative FC- values and down-regulation of transcripts. Color gray indicates that a predicted activation state of a gene/transcript in the canonical pathway signaling network is not affected. Note that only the top pathways are shown.

Supplementary file 18. Predicted effects on canonical pathways based on EV-RNA patterns (Figure 9 B) in plasma samples from patients with meningococcal septic shock versus healthy controls, and in Formalin-Fixed Paraffin-Embedded (FFPE) tissue samples with meningococcal septic shock vs. control patients.

Canonical Pathways	Z-score Plasma EV Nmsepsis	Z-score Lungs FFPE	Z-score Heart FFPE	Z-score Kidneys FFPE	Z-score Liver FFPE	Z-score Spleen FFPE
Neuroinflammation Signaling Pathway	3,00	3,21	3,87	1,90	1,67	N/A
Acute Phase Response Signaling	3,32	3,16	3,87	2,33	N/A	N/A
EIF2 Signaling	N/A	2,71	5,52	4,36	N/A	N/A
Oxidative Phosphorylation	3,05	2,65	N/A	3,74	N/A	2,24
IL-6 Signaling	3,16	3,00	3,36	1,41	N/A	N/A
IL-8 Signaling	2,00	2,45	3,00	1,00	2,24	0,00
Dendritic Cell Maturation	2,83	2,65	3,46	1,63	N/A	N/A
TREM1 Signaling	3,00	3,00	3,46	N/A	N/A	N/A
PPAR Signaling	-2,83	-2,12	-2,67	-1,63	N/A	N/A
HMGB1 Signaling	2,53	2,12	3,16	1,00	N/A	N/A
Role of IL-17F in Allergic Inflammatory Airway Diseases	2,83	2,83	2,83	N/A	N/A	N/A
Production of Nitric Oxide and Reactive Oxygen Species in Macrophages	2,67	2,24	2,65	0,00	0,82	N/A
iNOS Signaling	2,24	2,00	2,00	N/A	2,00	N/A
PPARα/RXRα Activation	-1,60	-1,89	-2,89	-1,63	N/A	N/A
PI3K/AKT Signaling	0,38	2,00	3,50	0,45	1,34	N/A
Role of NFAT in Regulation of the Immune Response	0,78	N/A	3,00	2,53	1,34	N/A
PI3K Signaling in B Lymphocytes	0,33	2,65	3,00	1,63	N/A	N/A
Cardiac Hypertrophy Signaling	0,26	2,65	2,24	2,00	N/A	N/A
B Cell Receptor Signaling	0,91	2,12	3,05	1,00	N/A	N/A
Opioid Signaling Pathway	-0,83	2,12	2,12	2,00	N/A	N/A
LXR/RXR Activation	-2,11	-0,71	-1,89	1,00	-1,34	N/A
Phospholipase C Signaling	-0,50	2,00	3,21	0,00	N/A	-1,34
NRF2-mediated Oxidative Stress Response	1,89	1,34	2,12	0,82	-0,82	N/A
Actin Cytoskeleton Signaling	-1,89	N/A	4,15	0,82	N/A	N/A
Type I Diabetes Mellitus Signaling	2,24	2,00	2,45	N/A	N/A	N/A
p38 MAPK Signaling	1,67	3,00	2,00	N/A	N/A	N/A
Signaling by Rho Family GTPases	0,28	N/A	3,50	1,13	1,63	N/A
Ephrin Receptor Signaling	0,30	2,45	3,32	0,45	N/A	N/A
CD28 Signaling in T Helper Cells	0,38	1,00	2,65	1,89	N/A	N/A
Integrin Signaling	-1,90	N/A	3,00	1,00	N/A	N/A
FAT10 Cancer Signaling Pathway	1,63	2,00	2,24	N/A	N/A	N/A
ILK Signaling	0,83	N/A	3,21	0,38	1,34	N/A
Rac Signaling	1,00	N/A	3,16	1,34	N/A	N/A
Osteoarthritis Pathway	0,78	1,73	2,50	0,45	N/A	N/A
RhoGDI Signaling	0,82	N/A	-3,21	-1,34	N/A	N/A
PTEN Signaling	1,34	N/A	-2,89	-1,00	N/A	N/A
Toll-like Receptor Signaling	1,89	2,33	1,00	N/A	N/A	N/A
Leukocyte Extravasation Signaling	0,63	N/A	2,32	N/A	2,24	N/A
Tec Kinase Signaling	0,00	2,24	2,89	N/A	N/A	N/A
Renin-Angiotensin Signaling	0,38	2,00	2,65	N/A	N/A	N/A
Th17 Activation Pathway	1,51	2,45	1,00	N/A	N/A	N/A
fMLP Signaling in Neutrophils	0,30	1,00	2,12	1,34	N/A	N/A
Apelin Endothelial Signaling Pathway	1,13	N/A	2,45	1,00	N/A	N/A
IL-17A Signaling in Airway Cells	0,82	1,63	2,12	N/A	N/A	N/A
iCOS-iCOSL Signaling in T Helper Cells	0,00	N/A	2,65	1,89	N/A	N/A
GNRH Signaling	0,00	1,34	1,13	2,00	N/A	N/A
Cdc42 Signaling	0,00	N/A	3,00	1,34	N/A	N/A
STAT3 Pathway	0,45	1,63	2,24	N/A	N/A	N/A
Fcγ Receptor-mediated Phagocytosis in Macrophages and Monocytes	1,63	N/A	2,53	N/A	N/A	N/A
Pancreatic Adenocarcinoma Signaling	2,00	N/A	2,12	N/A	N/A	N/A
RhoA Signaling	-0,45	N/A	2,53	1,00	N/A	N/A
Chemokine Signaling	0,71	2,24	N/A	1,00	N/A	N/A
Dopamine-DARPP32 Feedback in cAMP Signaling	-0,58	2,00	N/A	1,34	N/A	N/A
14-3-3-mediated Signaling	N/A	N/A	2,24	N/A	1,63	N/A
SPINK1 General Cancer Pathway	0,00	-2,53	-1,27	N/A	N/A	N/A
S100 Family Signaling Pathway	3,70	N/A	N/A	N/A	N/A	N/A
IL-1 Signaling	1,13	2,24	N/A	N/A	N/A	N/A
Regulation of Actin-based Motility by Rho	-0,82	N/A	2,53	N/A	N/A	N/A
Cell Cycle: G2/M DNA Damage Checkpoint Regulation	-2,00	N/A	1,34	N/A	N/A	N/A
Hypoxia Signaling in the Cardiovascular System	1,34	N/A	2,00	N/A	N/A	N/A
JAK/Stat Signaling	N/A	0,00	2,33	N/A	1,00	N/A
Mitochondrial Dysfunction	-3,02	N/A	N/A	N/A	N/A	N/A

Supplementary file 18. Predicted effects on canonical pathways based on EV-RNA patterns (Figure 9 B) in plasma samples from patients with meningococcal septic shock (denoted Plasma EV Nm sepsis) versus healthy controls, and in Formalin-Fixed Paraffin-Embedded (FFPE) tissue samples such as Lungs, Heart, Kidneys, Liver and Spleen from patients with meningococcal septic shock vs. control patients (acute non-infectious death). The table is based on the highest z-score values in the Plasma EV Nm sepsis group versus control group. Note that only the top pathways are shown.
